# A Method to Constrain Genome-Scale Models with ^13^C Labeling Data

**DOI:** 10.1371/journal.pcbi.1004363

**Published:** 2015-09-17

**Authors:** Héctor García Martín, Vinay Satish Kumar, Daniel Weaver, Amit Ghosh, Victor Chubukov, Aindrila Mukhopadhyay, Adam Arkin, Jay D. Keasling

**Affiliations:** 1 Physical Biosciences Division, Lawrence Berkeley National Laboratory, Berkeley, United States of America; 2 Joint BioEnergy Institute, Emeryville, United States of America; 3 Department of Bioengineering, University of California, Berkeley, Berkely, United States of America; 4 Department of Chemical Engineering, University of California, Berkeley, Berkeley, United States of America; The Pennsylvania State University, UNITED STATES

## Abstract

Current limitations in quantitatively predicting biological behavior hinder our efforts to engineer biological systems to produce biofuels and other desired chemicals. Here, we present a new method for calculating metabolic fluxes, key targets in metabolic engineering, that incorporates data from ^13^C labeling experiments and genome-scale models. The data from ^13^C labeling experiments provide strong flux constraints that eliminate the need to assume an evolutionary optimization principle such as the growth rate optimization assumption used in Flux Balance Analysis (FBA). This effective constraining is achieved by making the simple but biologically relevant assumption that flux flows from core to peripheral metabolism and does not flow back. The new method is significantly more robust than FBA with respect to errors in genome-scale model reconstruction. Furthermore, it can provide a comprehensive picture of metabolite balancing and predictions for unmeasured extracellular fluxes as constrained by ^13^C labeling data. A comparison shows that the results of this new method are similar to those found through ^13^C Metabolic Flux Analysis (^13^C MFA) for central carbon metabolism but, additionally, it provides flux estimates for peripheral metabolism. The extra validation gained by matching 48 relative labeling measurements is used to identify where and why several existing COnstraint Based Reconstruction and Analysis (COBRA) flux prediction algorithms fail. We demonstrate how to use this knowledge to refine these methods and improve their predictive capabilities. This method provides a reliable base upon which to improve the design of biological systems.

## Author Summary

While metabolic fluxes constitute the most direct window into a cell’s metabolism, their accurate measurement is non trivial. The gold standard for flux measurement involves providing a labeled feed where some of the carbon atoms have been substituted by isotopes with higher atomic mass (^13^C instead of ^12^C). The ensuing labeling found in intracellular metabolites is then used to computationally infer the metabolic fluxes that produced the observed pattern. However, this procedure is typically performed with small metabolic models encompassing only central carbon metabolism. The genomic revolution has afforded us easily available genomes and, with them, comprehensive genome-scale models of cellular metabolism. It would be desirable to use the ^13^C labeling experimental data to constrain genome-scale models: these data constrain fluxes very effectively and provide in the labeling data fit an obvious proof that the underlying model correctly explains measured quantities. Here, we introduce a rigorous, self-consistent method that uses the full amount of information contained in ^13^C labeling data to constrain fluxes for a genome-scale model where underlying assumptions are explicitly stated.

## Introduction

Systems biology aims to understand and predict how a cell’s behavior emerges from the interaction of its molecular parts [1–3]. Determination of metabolic fluxes (i.e., the number of metabolites traversing each biochemical reaction per unit time [4, 5]) is crucial to this effort because they map how carbon and electrons flow through metabolism to enable cell function [2, 5].

Metabolic fluxes typically cannot be measured directly, but must be inferred from experimental data through computational algorithms [4]. Among the most popular methods for studying metabolic fluxes are Metabolic Flux Analysis (MFA, [6, 7]), Flux Balance Analysis (FBA, [8]) and ^13^C Metabolic Flux Analysis (^13^C MFA, [4, 5, 9]). MFA calculates fluxes by using a stoichiometric model for the major intracellular reactions and assuming no metabolite accumulation [6]. The inputs are extracellular fluxes obtained through measurements of external concentrations of metabolites such as glucose or lactate as a function of time. If more flux measurements are available than degrees of freedom, the system is said to be overdetermined and a unique solution can be obtained. In the opposite case, the system is underdetermined and several flux profiles are compatible with the experimental data. MFA has been used to study fluxes in (e.g.) chinese hamster ovary [10], *S. cerevisiae* [11] and hybridoma cells [12].

Present-day FBA enhances MFA by expanding the network to include all reactions in metabolism, or at least as many as can be inferred from the genome through a metabolic reconstruction that yields a genome-scale stoichiometric model [13]. Since the degrees of freedom for such a model are usually over a hundred and measured fluxes are usually an order of magnitude less, the system is grossly underdetermined. Hence, fluxes are determined through linear programming (LP) by assuming that metabolism is tuned, due to evolutionary pressure, to maximize growth rate (typically; but see Schuetz *et al.* [14] for other suggested alternatives). The use of this objective function to interpret stoichiometric models is a key feature of FBA, even when stoichiometric models were not genome-scale. FBA forms the basis of a family of flux analysis methods named COnstraint-Based Reconstruction and Analysis (COBRA) methods [15], some of which can be used to produce flux predictions which are often used in bioengineering. These predictions can be full (when fluxes are determined without data from the actual experiment [16]) or partial (when some data from the experiment, like glucose consumption, is used for the prediction [17]), qualitative (e.g., prediction of whether an organism will grow or not under given conditions [18]) or quantitative (e.g., the value of the flux is predicted [19]). Full predictions are particularly useful for bioengineering purposes since they enable quick testing of the consequences of engineering approaches [20]. These methods have been used to facilitate the large-scale industrial production of 1,4-butanediol, a commodity chemical used to manufacture over 2.5 million tons annually of high-value polymers [21]. Recently, this rationally developed strain was used for a 5 million pound commercial production [22], and BASF has licensed this strain for future production of renewable 1,4-butanediol [23]. FBA has applications beyond bioengineering, including the prediction of ratios of microbial species in a simple microbial community [24] and providing valuable insights into tumor cell metabolism [25, 26].

^13^C MFA improves on MFA by using data obtained from ^13^C substrate labeling experiments together with a limited reaction stoichiometry and measured extracellular fluxes to measure intracellular fluxes. In these experiments, the organism under study is grown on ^13^C labeled substrate and the labeling pattern (i.e., the fraction of molecules with 0,1,2,… ^13^C atoms incorporated or Mass Distribution Vector, MDV [27]) is measured for a set of metabolites. Since the labeling pattern is highly dependent on the flux profile, it is possible to back-calculate the fluxes that best explain the measured labeling pattern if we know the fate of each carbon atom (carbon transitions, see [9]) for all reactions in the model. This involves a nonlinear fitting problem where the fluxes are the parameters. The usual approach is to consider only a small subset of all metabolism assumed to be comprehensive enough to explain the labeling of the measured metabolites, typically central carbon metabolism [28, 29]. Hence the model exhibits relatively few degrees of freedom that can be fully constrained by the labeling data. ^13^C MFA has found applications in metabolic engineering [30], biotechnology [31] and biomedicine [32].

Each of these methods involves its own advantages and disadvantages. Unlike ^13^C MFA, FBA uses the comprehensive description of metabolism contained in a genome-scale model and explicitly takes into account the system-wide balances of metabolites that can be crucial for host engineering [33]. Because of the exhaustive description of metabolism incorporated in genome-scale models, they often point towards completely unexpected regions of metabolism involved in the studied processes. For example, these models have been used to show that biosynthesis and degradation of heme compensates for the lack of a functional TCA cycles in cancer cells [26]. Furthermore, FBA can be used in combination with COBRA methods to make full predictions. ^13^C MFA, on the other hand, is a descriptive method for determining the metabolic fluxes compatible with the accrued experimental data but does not postulate general principles that can be used to make predictions for experiments that have not been performed. However, ^13^C MFA does not rely on maximum growth assumptions, the general applicability of which has been questioned [14, 34, 35] and shown to be inaccurate for engineered strains that are not under long-term evolutionary pressure [17]. Moreover, the comparison of measured and fit labeling patterns provides a degree of validation and falsifiability that FBA does not possess: an inadequate fit to the experimental data indicates that the underlying model assumptions are wrong. In contrast, FBA produces a solution for almost any input.

Several attempts to combine the complementary virtues of ^13^C MFA and FBA have been reported. For example, they have often been combined to test new FBA-based methods, since ^13^C MFA is considered to be the most authoritative determination of fluxes. Both Segrè *et al* [16] and Yizhak *et al* [19] used ^13^C MFA to validate MOMA and IOMA, respectively. More recently, Schuetz *et al* [14], used ^13^C MFA-derived fluxes to compare predictions from an MFA model containing ∼ 100 reactions and using different objectives, and also to demonstrate the applicability of pareto optimality to predict fluxes [35]. Choi *et al* [36] combined both methods by using flux ratios obtained from ^13^C MFA to constrain FBA for genome-scale models through the use of artificial metabolites. Although genome-scale models were not used, Suthers *et al* [27] presented ^13^C MFA for a large scale model containing 350 reactions. The OPENflux open source software by Quek *et al* [37], allows for certain reactions in ^13^C MFA to be used only for stoichiometric modeling purposes. More recently, Chen *et al* [38] for the first time modeled the same *E. coli* strain under the same conditions using FBA and ^13^C MFA, using some of the information of the latter to constrain the former. In a similar vein, Kuepfer *et al* [39] constrained a *S. cerevisiae* genome-scale model with fluxes obtained from ^13^C MFA and determined the flux distribution by using the minimization of the overall intracellular flux as the objective function.

However, to date there has been no attempt to use the data from ^13^C labeling experiments to constrain fluxes for a genome-scale model without assuming that metabolism is evolutionarily tuned to optimize an objective function (such as the growth rate optimization typically used in FBA). The traditional underlying assumption in the field of ^13^C MFA is that the degrees of freedom of the model must carefully match the amount of information obtained from the labeling patterns. Indeed, if the mathematical formulation of ^13^C MFA were that of a linear programming problem (as for FBA) it would be pointless to try to constrain over a hundred degrees of freedom with the approximately 50 measurements of labeling used in this study, for example. However, ^13^C MFA is a *nonlinear* fitting problem (with fluxes being the parameters) and these problems behave very differently for the underdetermined case, a special case of what is known as “sloppy” models in statistical mechanics [40–43]. These underdetermined nonlinear fits exhibit some degrees of freedom which are highly constrained (the parameters cannot be changed without noticeable measurable effects) and other degrees of freedom barely constrained at all (parameters can be changed freely, see section 3.3 in Brown *et al* [40] for a concrete example). Even if all degrees of freedom are not fully determined, the model can still be used to test hypotheses effectively [44]. These characteristics have been shown to be of general nature and apply to a variety of nonlinear problems appearing in systems biology [44], insect flight and variational quantum wave functions [45], interatomic potentials [46] and a model of the next-generation international linear collider [42]. In this paper, we present a systematic and rigorous framework to take full advantage of the nonlinear nature of the flux fitting problem and find all fluxes compatible with the ^13^C labeling data for a genome-scale model. In order to constrain fluxes effectively, we make the biologically relevant assumption that metabolic flux flows from core metabolism (defined below) to peripheral metabolism and does not flow back.

**Fig 1:**
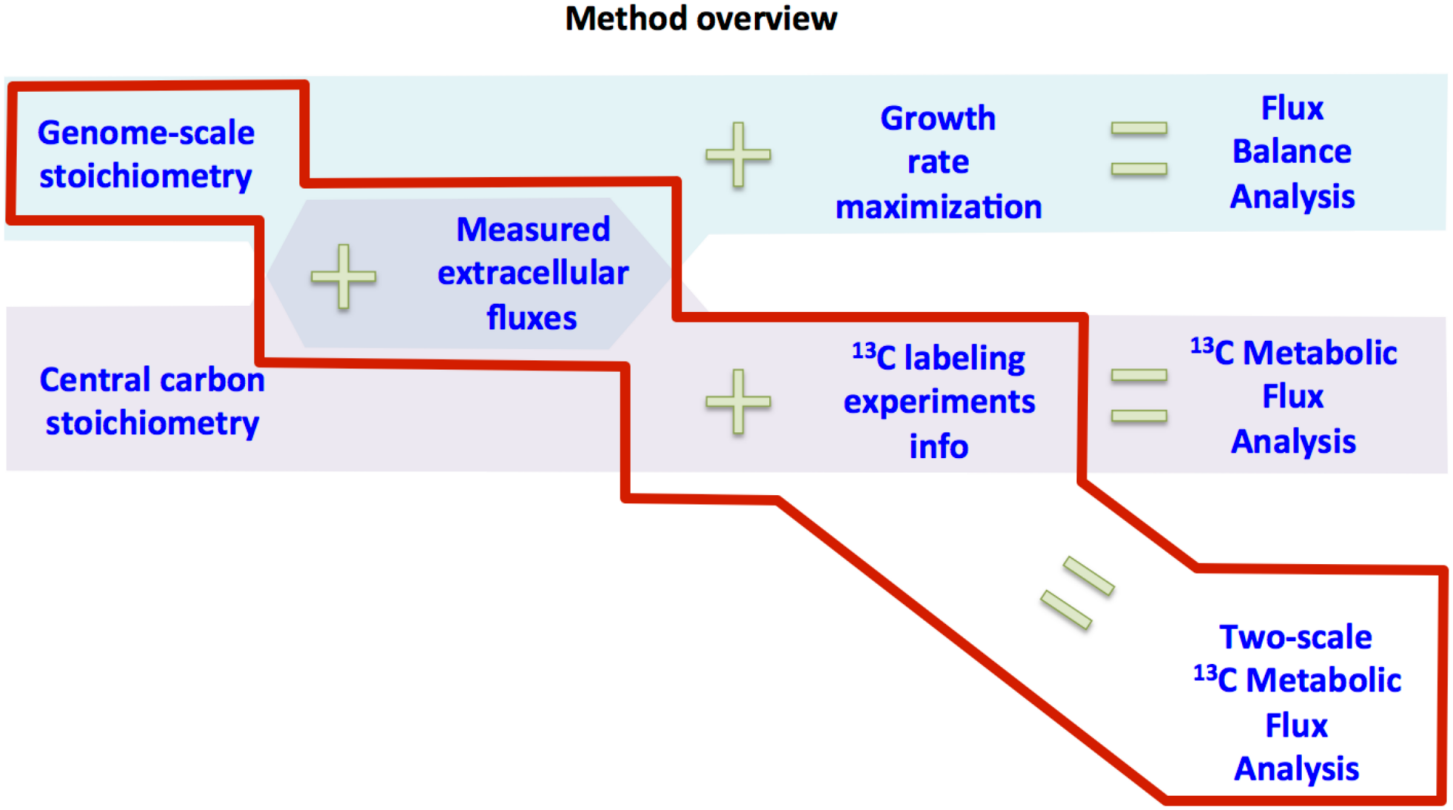
Method overview. FBA uses genome-scale stoichiometry and measured extracellular fluxes to constrain fluxes, which are finally determined by assuming growth rate maximization. ^13^C MFA measures fluxes by using the highly informative ^13^C labeling experimental data along with central carbon stoichiometry and measured extracellular fluxes. 2S-^13^C MFA calculates fluxes by using the ^13^C labeling experimental data and the measured extracellular fluxes to constrain fluxes for a genome-scale model.

The simultaneous use of ^13^C labeling data and genome-scale models produces a new computational approach combining the advantages of both FBA and ^13^C MFA: two-scale ^13^C Metabolic Flux Analysis (2S-^13^C MFA, see Fig 1). 2S-^13^C MFA determines fluxes for a full genome-scale model, taking into account the system-wide balances of metabolites. However, 2S-^13^C MFA does not rely on maximum growth assumptions: instead it uses the data obtained from ^13^C labeling experiments to constrain feasible fluxes. The use of this data is shown to constrain glycolytic and pentose phosphate pathway fluxes 8–50 fold more effectively than using only measured extracellular fluxes. We use this new method to compare different predictive methods and show where and why they fail. Based on that information, we develop a new predictive method that is able to produce a full quantitative prediction of 48 labeling measurements, going beyond the usual qualitative (e.g. grow/no grow) predictions.

## Results and Discussion

### Constraining genome-scale models with ^13^C labeling data

In ^13^C MFA, most reactions in the cell’s enormous metabolic network have a very limited contribution to the ^13^C labeling of the observed metabolites (regardless of whether these are free metabolites [47] or proteogenic amino acids [4, 9]). Thus, since the complexity of the problem of flux measurement from labeling information scales nonlinearly with the number of reactions, the metabolic network in ^13^C MFA consists of a minimal set of reactions (core set) that most influence labeling patterns (typically central carbon metabolism but may include other extra reactions, such as those describing protein turnover [48]), stemming from the literature or the researcher’s experience. This approach is able to convincingly explain labeling patterns for amino acids and intracellular metabolites for model organisms (e.g. *E. coli* [49, 50] and *S. cerevisiae* [51, 52]) under well-studied conditions (e.g. glucose feed). The good fits to experimental data support, to a good approximation, the underlying assumptions that carbon precursors flow from a central core metabolism to peripheral metabolism and do not flow back. However, it would be desirable to generate the minimal core network systematically through a computational method, so the technique can be generally applied to non-standard cases: e.g. non-model organisms, bioengineered strains, alternative non-standard carbon sources, human cells or microbial communities. Most crucially, such a method would explicitly test the assumption that reactions not included in the minimal network do not significantly influence labeling. As an added benefit, it would take advantage of the significant community efforts that have been put into developing and improving genome-scale metabolic models [53].

Another important benefit of integrating ^13^C MFA with genome-scale metabolic models is to check for consistency of the inferred core fluxes with peripheral metabolism. The central metabolic network needs to produce not only carbon precursors for peripheral metabolism, but also ATP and reducing equivalents. Genome scale networks offer the possibility to track every reaction consuming and producing energy or reducing equivalents, and therefore can be used effectively to study the interplay between peripheral and core metabolism. On one hand, since core metabolism involves the reactions with largest fluxes in metabolism, once these are set by the ^13^C labeling data, peripheral metabolism is expected to be highly constrained. On the other hand, changes in the usage of core intermediates in peripheral metabolism will also have significant effects on core fluxes since they need to provide these carbon intermediates, on top of ATP and reducing equivalents. While this has been already predicted in terms of small changes in biomass composition having significant effects on central carbon fluxes [54], the effect is an order of magnitude more important in bioengineered strains [55]. In such cells, peripheral metabolism can be vastly altered, and it is crucial to have a method that can take the diverse changes into account in a systematic manner.

**Fig 2:**
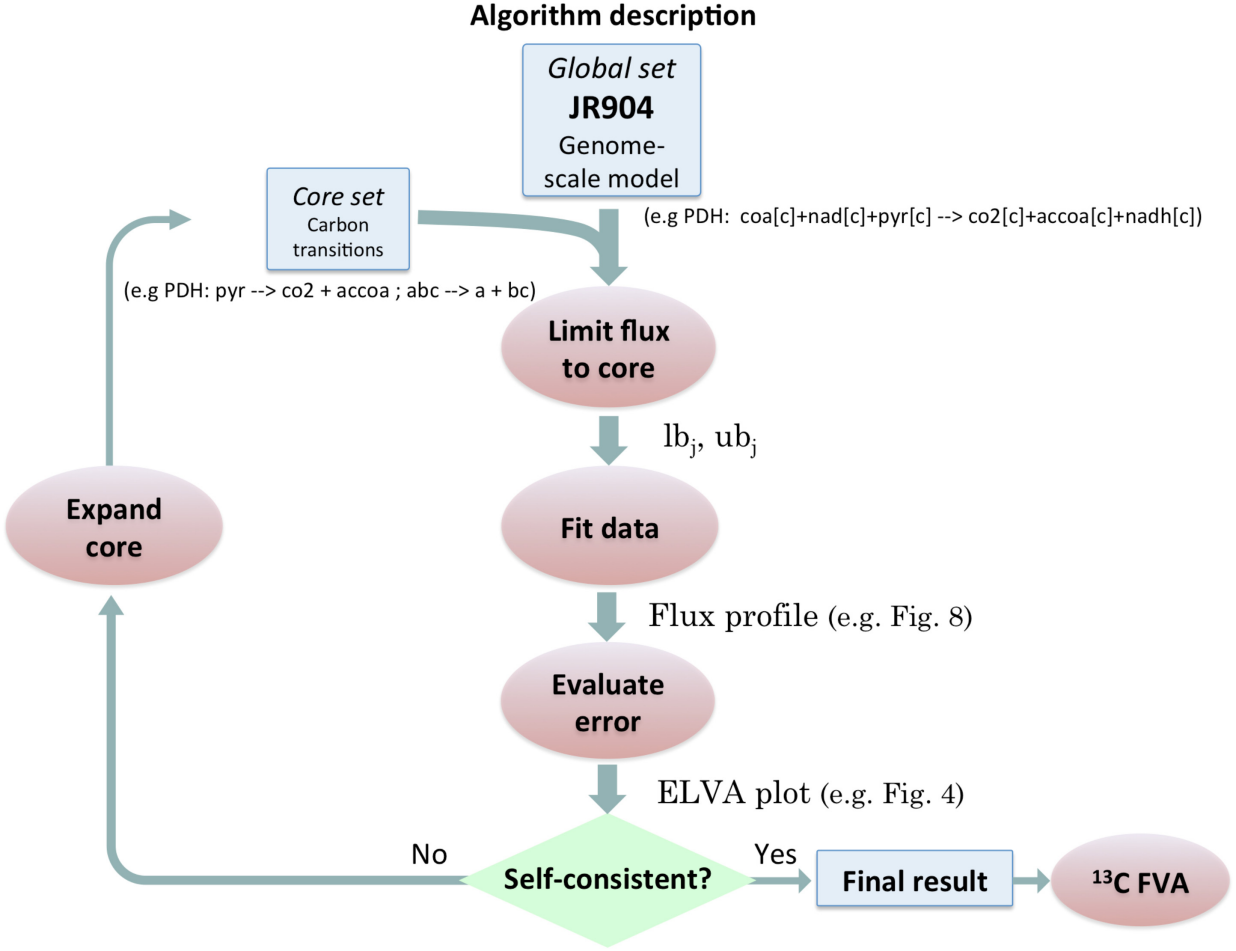
Algorithm description. Algorithm flow diagram for 2S-^13^C MFA showing a recursive procedure to achieve self-consistent results. The full model consists of a genome-scale model (iJR904 in this case) to which information on carbon transitions for the core sets of reactions is added (blue box on the left). The genome-scale model carries the measured extracellular fluxes information as upper and lower bounds (*ub*_*j*_ and *lb*_*j*_). Carbon transitions (example line below the blue box) indicate the fate of each carbon atom in the reaction. The first step in the algorithm involves limiting the amount of flux that flows into the core set of metabolites and reactions, so as to enforce the two-scale approximation (i.e. that non-core contributions to labeling are negligible). The second step involves finding the set of fluxes that best fit the experimentally observed data, ignoring the non-core contributions. The final step tests that the error incurred by ignoring non-core reactions is negligible through External Labeling Variability Analysis (ELVA). If the ELVA does not indicate that the non-core contributions are negligible, the core set and the EMU model are expanded and the procedure repeated. When a self-consistent result is found, flux ranges compatible with the experimental data are obtained through ^13^C Flux Variability Analysis.

**Fig 3:**
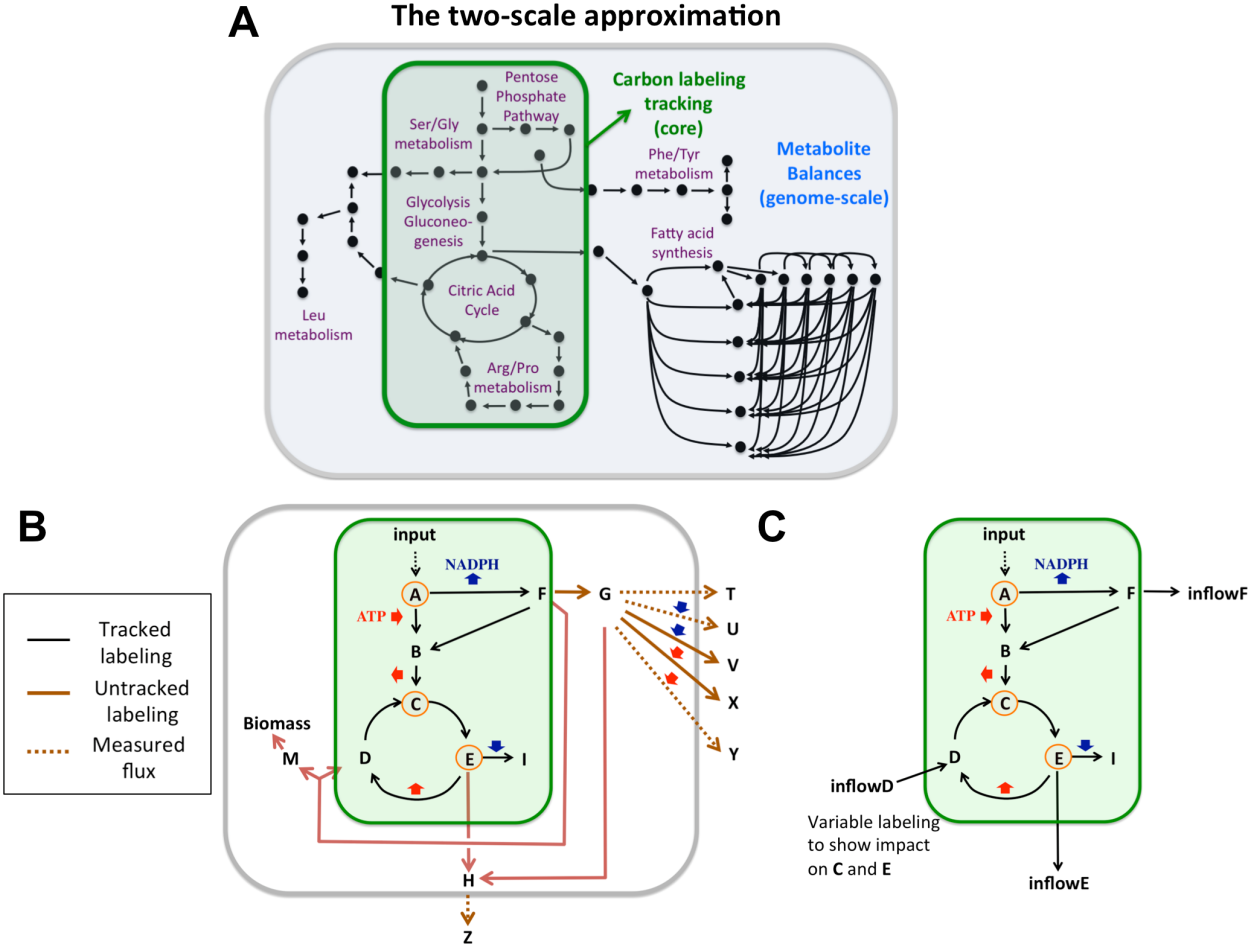
The two-scale approximation. **A)** 2S-^13^C MFA models microbial metabolism at two different scales of resolution, hence minimizing the computational effort to explain the experimental data. While stoichiometric balances are taken into account for the full genome-scale model (iJR904 in this case), metabolite labeling originating from the ^13^C feed in the labeling experiments is only tracked for the core set of reactions responsible for the main fraction of metabolite labeling (green box). The two-scale approximation assumes that non-core metabolites do not directly affect core metabolite labeling. The core set is expandable through the recursive procedure shown in Fig 2. **B)** Exemplary network of 20 reactions that illustrates the two-scale approximation and the approach. Measured data involves the MDV for metabolites A, C and E and extracellular fluxes for reactions producing metabolites T, U, Y and Z. The initial core set involves reactions and metabolites in the green box. The fit involves finding fluxes which best match the measured labeling and the values of the measured extracellular fluxes, where only the contribution of reactions inside the green box is taken into account to fit the labeling of metabolites A, C and E. However, the metabolite balance is global. In this way the fluxes are not overconstrained by e.g. NADPH balance: any excess NADPH can be balanced by the non-core fluxes that consume NADPH. **C)** Right lower panel illustrates External Labeling Variability Analysis (ELVA) for the exemplary network. ELVA gauges the effect of non-core reactions by considering only the core network and simulating the impact of non-core metabolite labeling through inflow metabolites (inflowD, inflowE, inflowF). The ELVA optimization problem (Eqs 9–15) finds the maximum impact that the unknown inflow metabolite labeling can have on the measured labeling pattern.

2S-^13^C MFA addresses both of these issues and provides a complete rigorous framework for estimating fluxes using ^13^C labeling data in the context of a genome scale metabolic network. The algorithm is comprised of a set of optimization problems to be applied sequentially, as shown in the outline in Fig 2. Our approach divides the metabolites and reactions in a genome-scale model into two groups to be modeled at different scales of resolution (Fig 3A), in the spirit of the multi-scale approach in engineering and physics [56]. For “core” metabolites and reactions both stoichiometry and carbon labeling are tracked. For the remaining “non-core” metabolites and reactions, only stoichiometry is tracked and their contribution to the core set labeling is ignored. Crucially, the algorithm recursively adjusts the division into core and non-core reactions according to the error in the labeling fitting. This procedure is illustrated with a small illustrative network (see Fig 3B and 3C) throughout the following subsections. Notice that, because of this core readjustment and the inclusion of other reactions needed to fully explain labeling [48], the core set needs not be the same as is usually referred to as central carbon metabolism.

#### Limiting flux to core reactions

2S-^13^C MFA starts with an initial guess for the core set and then limits the flux into core metabolism, so as to be consistent with the initial assumption that non-core metabolism does not directly contribute to core ^13^C labeling. We will refer to this assumption as the two-scale approximation or assumption. In the context of a genome-scale model (iJR904 [57] in this particular case, see Materials and Methods), this assumption amounts to limiting the upper bound of all reactions with products in core metabolism to zero, or the lowest value consistent with the observed growth rate (see Materials and Methods for the technical details).

Constraints introduced in this step replicate the same conditions as for ^13^C MFA: by limiting the flux into core reactions, the degrees of freedom for the system of core reactions are similar to the case of standard ^13^C MFA. This fact is reflected in very similar solutions for the core, as explained in the “Comparison with ^13^C MFA” section. Hence, this is not a radically new assumption, but rather the same underlying assumption used in ^13^C MFA. 2S-^13^C MFA simply explores its consequences in the context of genome-scale models. The degrees of freedom for the core system only differ from ^13^C MFA because of the availability of more reactions to flow out of the core (since these are not set to zero). Rather than having the outgoing fluxes being prescribed by the modeler, 2S-^13^C MFA allows the system to channel this outgoing flux as it best fits the available data. This can lead to unforeseen results, as in the glycolate case shown in the “Comparison with ^13^C MFA” section. Furthermore, these constraints automatically eliminate some of the biologically unrealistic solutions that have been shown to appear in fully unconstrained large-scale ^13^C MFA studies [27].

In the illustrative network (Fig 3B), the core set of reactions is initially chosen as the reactions in the green box, which are expected to appropriately explain the labeling of the measured metabolites (A, C and E, in orange circles). The first step (“Limit flux to core”) involves setting to zero the flux of the reaction flowing from metabolite F to D. However, this reaction produces a metabolite M that is needed for cell growth, so it cannot be fully set to zero. A minimal level of flux is allowed (e.g. 5% of the glucose uptake rate), as shown in the “Limiting flux to core” section in materials and methods.

#### Fitting experimental labeling data

The second step fits the experimentally determined labeling data through the Elementary Metabolite Unit (EMU) method [28, 58]. While in this study we use labeling of central metabolic intermediates, the framework could be equally well applied to free amino acids or amino acids from hydrolyzed protein. The constraints include the experimentally determined growth, uptake, and secretion rates. The data fit is set as a NonLinear Programming (NLP) optimization problem that minimizes the difference between computational and experimental labeling values and uses a combination of constraints from FBA and ^13^C MFA, as shown in Eqs 1–7 (see S1 Text). Results can be seen in S1–S3 and S4–S12 Figs. Unlike FBA, there is no growth (or other flux) maximization involved: the growth rate is constrained to its measured value. Unlike ^13^C MFA, the full genome-scale model reaction fluxes are used in the fit although, as explained below and observed in other nonlinear fits [27, 41, 42], not all fluxes are fully determined.

For the illustrative model, this step involves finding the fluxes that best fit the measured labeling of metabolites A, C and E where the labeling is calculated from only the contributions of the reactions in the green box (core) but metabolite balances are imposed for all reactions. In this way, instead of NADPH overconstraining the core fluxes by forcing the production inside the core (reaction A to F) to equal the consumption (reaction E to I), these fluxes are free to fit the experimental data while excess NADPH can be compensated by the reactions outside the core.

#### Self-consistency test through External Labeling Variability Analysis (ELVA)

The key next step of 2S-^13^C MFA then determines if the fluxes obtained from the fit are consistent with the two-scale approximation. This extra procurement sets the method apart from ^13^C MFA, which does not check the effect of ignored reactions, even for large-scale models [27] or by marking some reactions to be excluded from the isotopomer balance [37]. The impact of non-core reactions is calculated through External Labeling Variability Analysis (ELVA, see Eqs 9–15). ELVA considers only core metabolism with the impact of non-core metabolism being represented through inflow metabolites, dummy metabolites with unfixed labeling since their labeling is, by definition, unknown (see Fig 3C for an example). Essentially, using the previously obtained genome-scale flux solution as a constraint, we allow the labeling for the inflow metabolites to vary and, for each metabolite with measured labeling, use each element of the mass distribution vector (MDV) as the objective function to be maximized or minimized. By this method we obtain a confidence interval that represents the maximum possible difference in labeling that could be attributed to non-core reactions for the current solution. The reactions that contribute an unacceptable amount of uncertainty are then added to the core set and the procedure can be repeated as necessary, until a core set of reactions is found which fully justifies the two-scale approximation.

In the case of the illustrative model, the use of the ELVA would gauge the impact of non-core reactions as described in Fig 3C. The ELVA would point out that the reaction transforming metabolite F to D and M needs to be included in the core: this reaction strongly influences the labeling of metabolite D, which in turn influences the labeling of metabolite C, which is being measured. This effect would be shown in large computational error bars for metabolite C through the influence of inflow metabolite D. After the reaction taking metabolite F to D and M is added to the core, a new fit would be in order (as shown in Fig 2), and the subsequent ELVA would show zero computational error, since none of the remaining reactions flow back into the core and can impact the labeling of the measured metabolites A, C and E.

**Fig 4:**
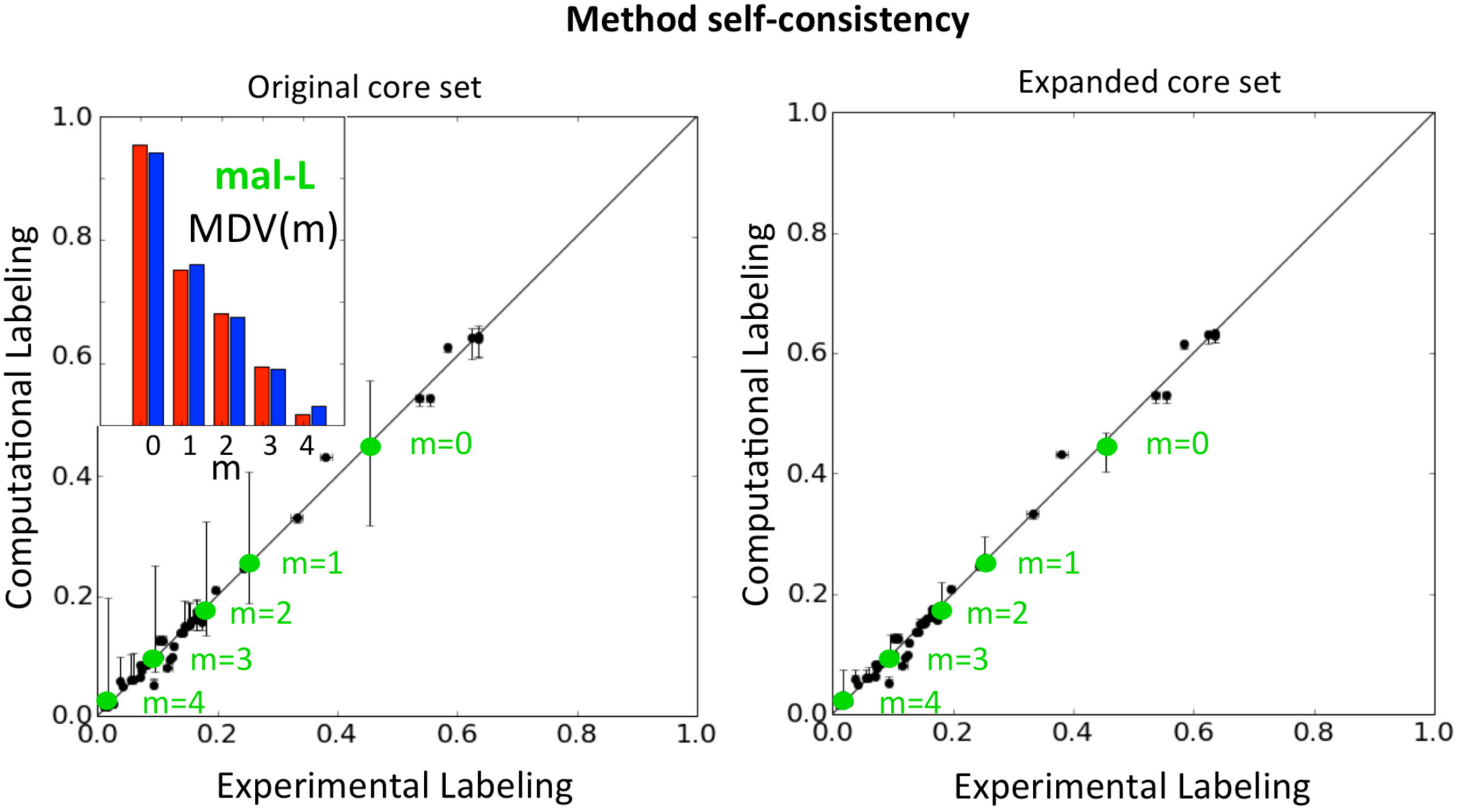
Method self-consistency. External Labeling Variability Analysis (ELVA, see methods) shows how the impact of ignored reactions diminishes by expanding the core set of reactions. Each point corresponds to an *m* value of the Mass Distribution Vector (MDV) for each of the metabolites considered. The inset provides the same information for malate in a more intuitive form (red for experimental data, blue for computational fits), see S1–S3 Figs. Horizontal error bars indicate experimental CE-TOFMS error obtained from the instrument. Vertical error bars indicate computational errors obtained from the ELVA. These computational error bars indicate the maximum effect that non-core reactions (whose contribution to the carbon labeling is being ignored) could possibly have. The initial core set (left) shows a large computational error for malate (mal-L, green dots). By expanding the core set, the computational errors collapse to levels comparable with the experimental error as can be seen in the right panel. Hence, the method is self-consistent by ensuring that the final result meets the approximation used to calculate it.

To further illustrate this procedure with experimental data, we fit the labeling data from Toya *et al* [47] for wild-type *E. coli* during mid-exponential phase growth on glucose. Using an initial set of 94 central metabolic reactions (see S3 Text), we calculated the flux profiles and ELVA confidence intervals as described above. As seen in Fig 4 (left panel), several points had unacceptably wide confidence intervals (vertical error bars), in many cases over an order of magnitude greater than the experimental uncertainty (horizontal error bars), illustrating that reactions not included in the core could significantly influence the measured labeling. All of the most pronounced effects corresponded to the MDV of the TCA cycle intermediate malate (green dots on Fig 4). These effects were found (by inspection) to be due to reactions involved in glutamate, arginine, histidine metabolism and nucleotide biosynthesis, many of which use aspartate as a nitrogen donor, releasing fumarate. Since fumarate is converted to malate in the TCA cycle, this aspartate → fumarate flux, often ignored in traditional ^13^C MFA approaches [59–62], has a potentially important impact on the labeling of malate and must be considered as part of the core set, as some other studies have done [63]. Inclusion of these pathways in the core set dramatically narrowed the confidence intervals of the ELVA analysis (right panel of Fig 4), of the same order of magnitude now as the experimental error. This implies that the revised core (with a total of 126 reactions) satisfies the two-scale approximation, meaning that peripheral reactions not included in the core set cannot significantly influence observed labeling patterns. Hence, this method is self consistent and, furthermore, points out which reactions need to be added to the core set to make the approximation valid.

#### Confidence intervals provide all fluxes compatible with experimental data

^13^C labeling information is generally insufficient to completely constrain all fluxes in the network, meaning that a space of distinct flux solutions is consistent with the observed labeling. As such, the goal of a good flux estimation algorithm should be to provide the range of flux values that are consistent with the observed data, rather than a single “best” solution. This is particularly important in the case when flux estimation is expanded to the entire genome-scale model, with the corresponding dramatic increase in the degrees of freedom. 2S-^13^C MFA addresses this directly through ^13^C Flux Variability Analysis (^13^C FVA): as shown in Eqs 16–23, each flux in the genome-scale model is maximized and minimized to find the range of fluxes producing labeling patterns within the experimental error of the measured values (and in the context of the two-scale approximation).

**Fig 5:**
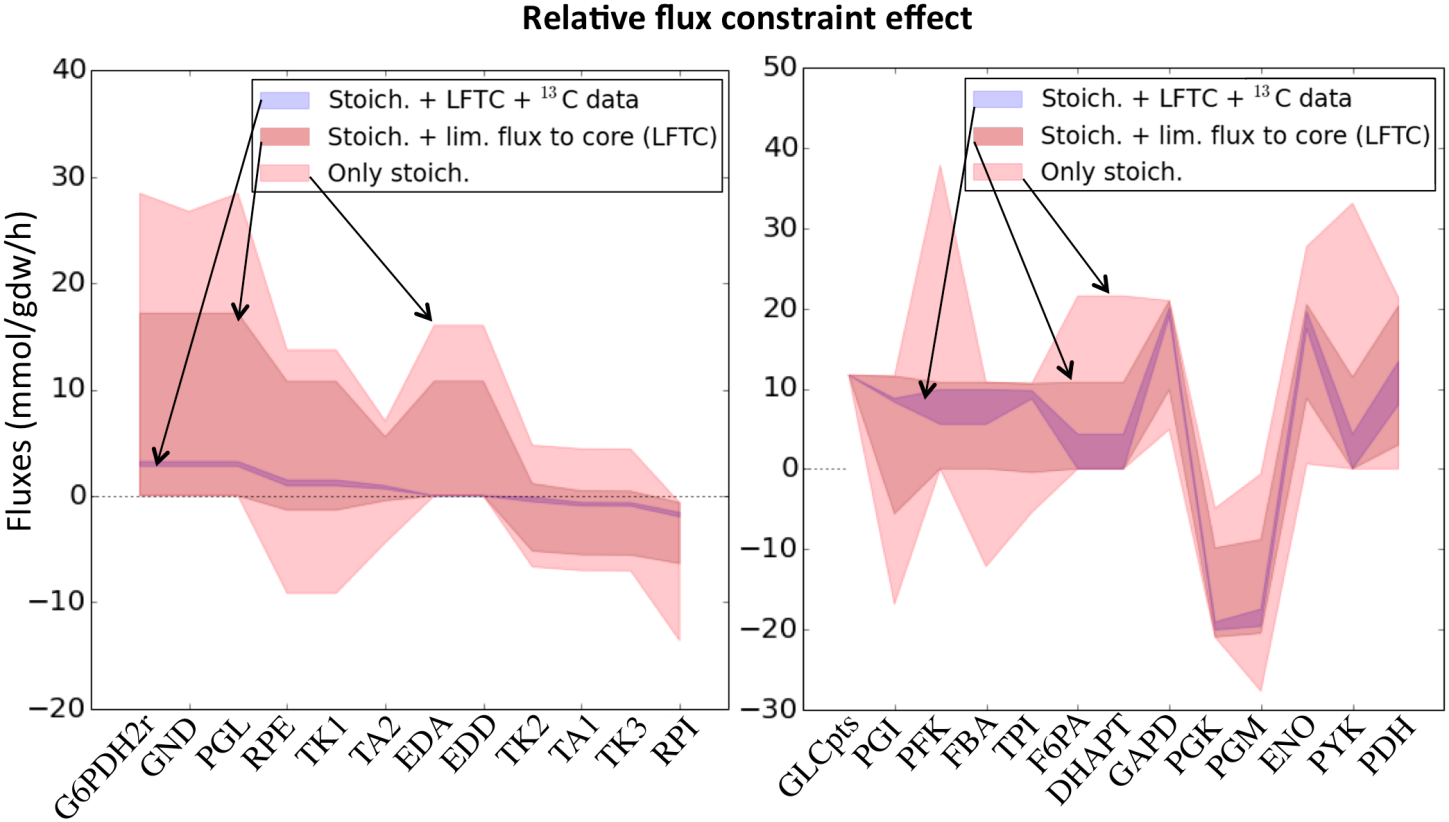
Relative effect of constraints. Confidence intervals for pentose phosphate pathway (left panel) and glycolysis (right panel) fluxes calculated using FBA constrained by measured extracellular fluxes (through Flux Variability Analysis, FVA [64], in red), for FBA with constraints derived from the two-scale approximation through the “Limit flux to core” step in Fig 3 (FVA, in grey), and for 2S-^13^C MFA derived through Eqs 16–23 are shown (blue). Constraints induced by the two-scale approximation are not strong, hence justifying the use of this approximation. However, constraints induced by the ^13^C labeling data are dominant. A similar pattern can be observed in S13 Fig.

The capability of the ^13^C labeling data to constrain fluxes is very significant, as can be seen in Fig 5: the ranges of fluxes compatible with the ^13^C labeling data and the measured extracellular flux data is much smaller than those compatible with only the measured extracellular flux data. In fact, the average flux confidence interval for 2S-^13^C MFA relative to the same interval for fluxes constrained only by extracelllular fluxes is ∼ 2% for the pentose phosphate pathway and ∼ 12% for glycolysis. Hence, 2S-^13^C MFA constrains fluxes 8–50 fold more effectively for these cases. Furthermore, we can see that the “limit flux to core” step does not introduce very strong constraints on the allowable fluxes.

**Fig 6:**
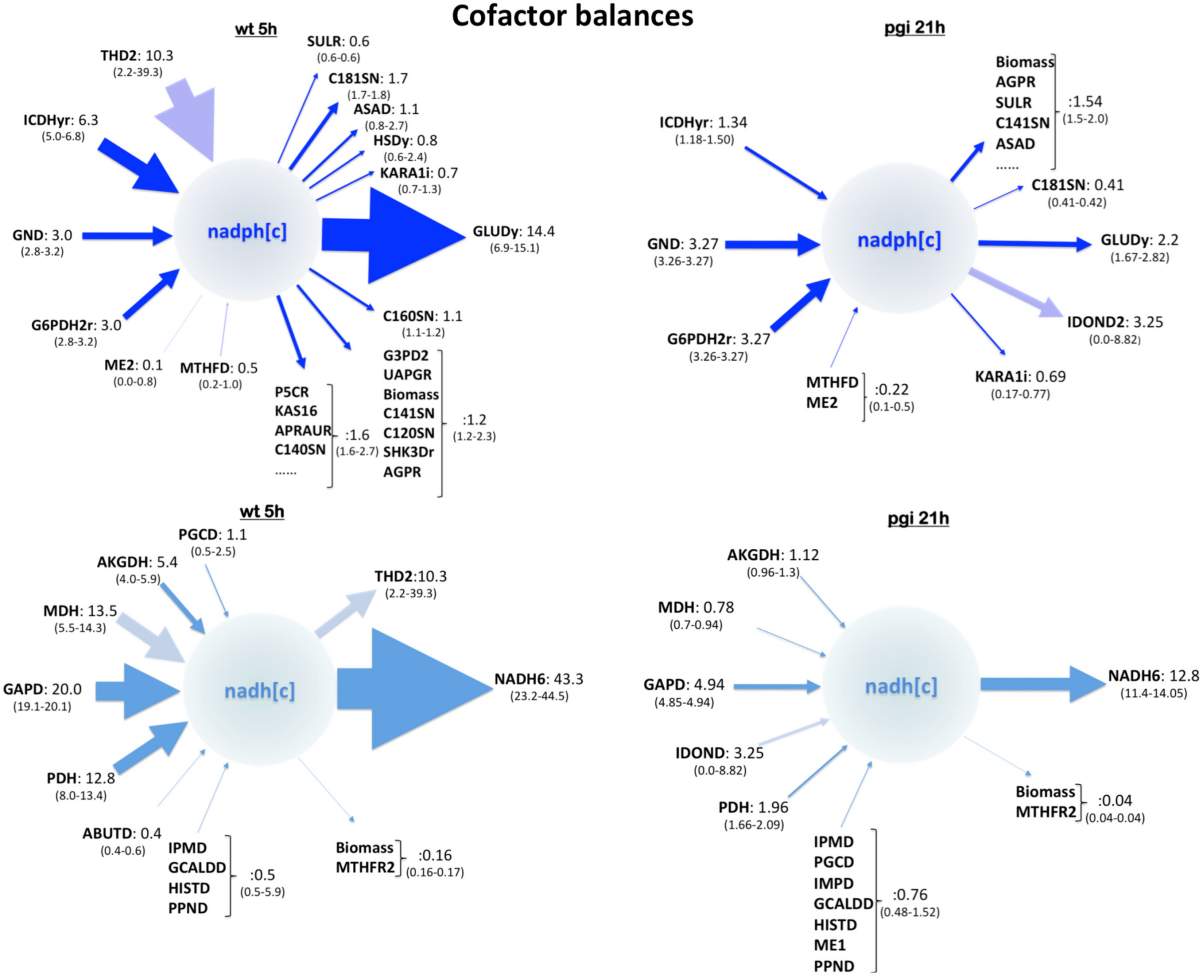
Cofactor balances. Cofactor balances show how NADPH and NADH production and consumption change after *pgi* is knocked out. Arrows pointing inwards on the left indicate fluxes that produce the indicated metabolite and fluxes pointing outwards on the right indicate fluxes that consume it (in units of mMol/gdw/hr). Reaction names are per iJR904 model. Upper panels show NADPH balances for wild type (left) and *pgi* KO (right) at 5 and 21hr (equivalent growth points due to a lower growth rate in the *pgi* KO). Lower panels show NADH balances for wild type (left) and *pgi* KO (right) at the same time points. Note that, unlike FBA, 2S-^13^C MFA can provide confidence intervals bounded by the data from ^13^C labeling experiments. These are shown below the reaction name. For some cases (e.g. GND) the experimental data can very effectively constrain the flux value, even if the reaction is not in the core set over which labeling is being tracked (e.g. C181SN). For some others (e.g. THD2), the data can only constrain the flux value in a very limited fashion. Knocking out *pgi* radically changes NADPH and NADH supply and consumption patterns.

Not all fluxes in the genome-scale model are effectively constrained by the ^13^C labeling experiment data. This phenomenon is a general characteristic of nonlinear fits of underdetermined (“sloppy”) systems, and is expected. We see that, for example, flux values for most reactions in the core set are effectively constrained (i.e., the confidence intervals are narrow, as for GAPD in lower left panel of Fig 6). These core constraints are propagated to the rest of the fluxes through stoichiometry, and some non-core fluxes are also well determined (e.g., C160SN in upper left panel in Fig 6) while others are not (e.g., THD2 in upper left panel of Fig 6). As is often the case in fitting problems, we expect the degree to which this method can constrain fluxes to depend heavily on the available data: labeling distribution for more intracellular metabolites or measurements for more extracellular fluxes will increase the flux resolution (i.e., decrease the confidence intervals).

For the exemplary model, confidence intervals would be obtained through the optimization problem described in Eqs 16–23. If metabolite labeling data for new metabolites (e.g. D) became available, that information would decrease the confidence interval of the fluxes (and the core would have to be redefined).

### Robustness with respect to data input

2S-^13^C MFA is supported by two independent data sets: ^13^C labeling experimental data and genome-scale stoichiometry. We will now show that the method is robust to errors in the ^13^C labeling experimental data and more robust than FBA to stoichiometric errors.

**Fig 7:**
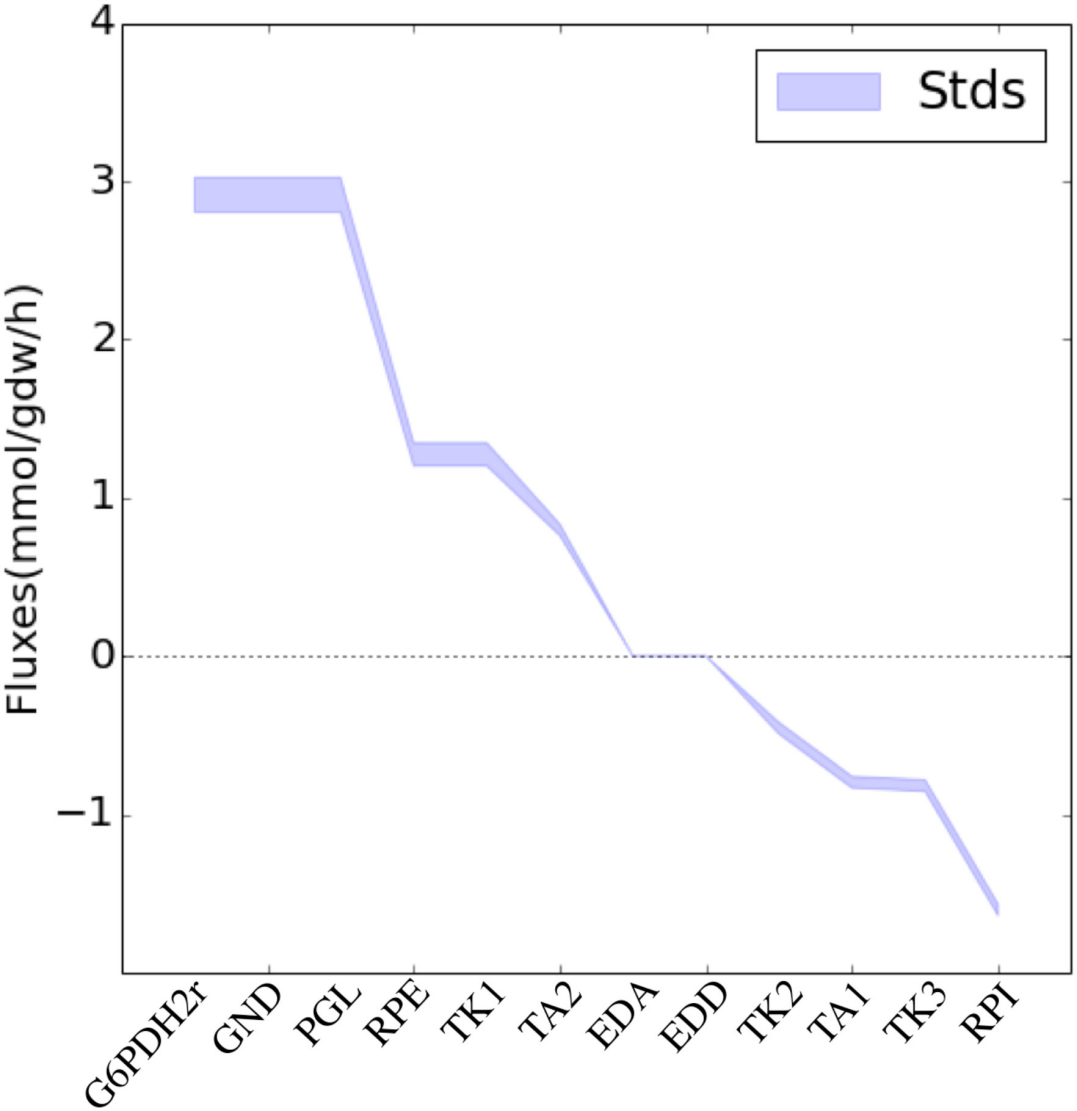
Robustness with respect to measurement error in labeling profile. 30 different new labeling data sets were generated by randomly choosing new labeling values within the experimental error (see equation 13 in S2 Text). Fluxes were calculated through 2S-^13^C MFA for these new data sets and the standard deviation is shown for the PPP. Hence, the method is robust with respect to experiment accuracy in ^13^C labeling.

Fluxes calculated through 2S-^13^C MFA are robust with respect to the experimental accuracy of the ^13^C labeling data. We show this by generating new sets of ^13^C data where the new labeling is randomly chosen within the experimental error. The calculated profiles do not change for this new labeling significantly from the initial profiles, as can be observed in Fig 7.

**Fig 8:**
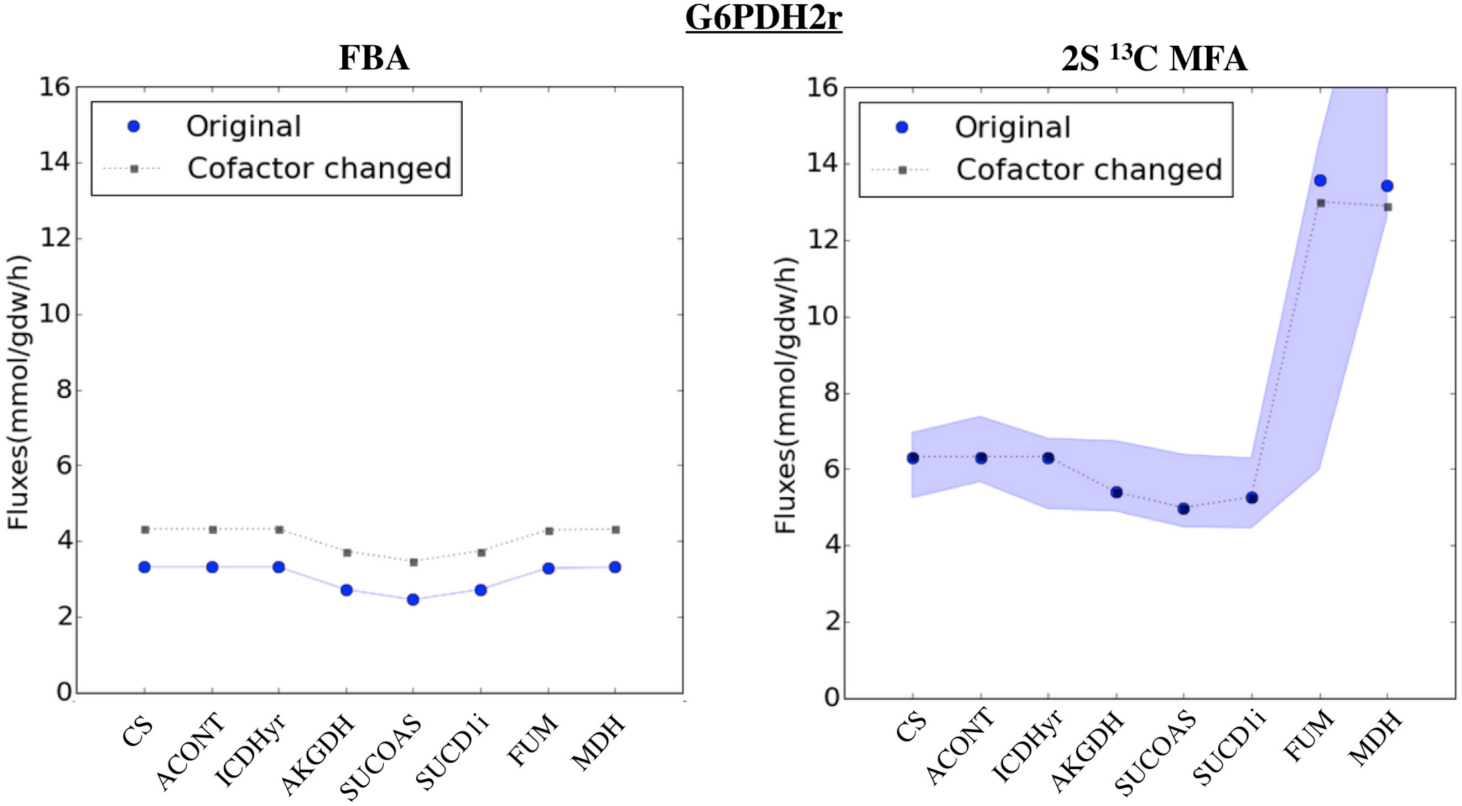
Robustness with respect to genome reconstruction errors. A reconstruction error was simulated by changing the NADPH dependence to NADH dependence for the glucose-6-phosphate dehydrogenase (with a large flux value of 2.9 mMol/gdw/hr in the initial 2S-^13^C MFA calculation). We calculated fluxes again through 2S-^13^C MFA and FBA (constrained by extracellular flux measurements), and the new fluxes are plotted for the TCA cycle. As can be observed, the change is much larger for FBA than 2S-^13^C MFA, showing that it is less robust to reconstruction errors (note that squares and circles are almost on top of each other for the 2S-^13^C MFA case). The transparency in the original flux profile for 2S-^13^C MFA indicates the confidence intervals. For the 2S-^13^C MFA case, the NADPH production shift is compensated entirely by the THD2 transhydrogenase. Since the flux value for SUCD1i is negative, the absolute value has been plotted. Similar figures for the glutamate dehydrogenase (GLUDy) and isocitrate dehydrogenase (ICDHyr) reactions are available as S16 Fig.

Cofactor ambiguities in the reconstruction of genome-scale models introduce errors in determining fluxes which are much smaller than for FBA. Reconstruction errors are still possible in genome-scale models, in spite of the care used to develop them and the continous improvement in the reconstruction for each new release [13]. We studied the resulting flux profiles after a reconstruction error was simulated (by changing NADPH to NADH dependence for a high flux reaction, G6PDH2r) and found that the change in fluxes was much less severe than for FBA (Fig 8). We found similar results for other reactions (S16 Fig). We attribute this difference to FBA relying heavily on stoichiometric information whereas 2S-^13^C MFA can additionally count on ^13^C labeling data to constrain fluxes. Furthermore, changes in the biomass composition reaction do not affect calculated fluxes significantly (S17 Fig).

### Cofactor balances

2S-^13^C MFA can produce cofactor balance information which cannot be produced using either FBA (does not provide confidence intervals bounded by ^13^C data) or ^13^C MFA (does not consider all possible cofactor-producing reactions in the cell metabolism). Discerning cofactor production and consumption can be crucial to understand cell behavior. Not only does cofactor balancing have an essential bearing on tumor cell death [65], but it can also have a significant impact on final production for bioengineered cells [33, 55, 66, 67]. Core metabolism and biosynthetic pathways are linked through cofactor balancing, so an expansion of ^13^C MFA to include genome-scale stoichiometry such as that provided by 2S-^13^C MFA is of great interest for bioengineering. While some previous ^13^C MFA efforts [27, 28, 37] did take cofactor balances into account, this has not been done for all metabolites in a genome-scale network.

This capability is illustrated (Fig 6) with the main electron carriers in metabolism, NADPH and NADH, responsible for redox balances and, ultimately, for respiration. A majority of NADPH production (47–55%, obtained by normalizing data from Fig 6) in wild type *E. coli* is produced by the pentose phosphate pathway (reactions G6PDH2r and GND: 25–27%) and the TCA cycle (reaction ICDHyr: 22–29%). Notice that, unlike FBA, 2S-^13^C MFA produces confidence intervals bounded by ^13^C data: for example, if the flux through reaction GND were to be more than 3.2 mMol/gdw/hr (30% of total NADPH creation according to upper left part of Fig 6), the computationally derived labeling for (e.g.) *m* = 0,4 for malate (mal-L), for *m* = 1 for phosphoenolpyruvate (pep) and for *m* = 1,2 for ribose 5-phosphate (r5p) would differ from the measured value by more than the CEMS measuring error (see Eq 23 and S1–S3 Figs). The transhydrogenase reaction THD2, transferring electrons from NADH to NADP, also contributes significantly (∼ 44%) but, unfortunately, the flux through this reaction is only very loosely constrained by the current data set. The main NADPH sink involves glutamate production (reaction GLUDy: 29–65%), with fatty acid biosynthesis (C181SN) in a distant second place (7%) and a collection of other reactions at equally low levels (< 12%).

NADPH consumption and production are radically altered when the gene encoding the initial enzyme in glycolysis (PGI) is knocked out (Fig 6, right upper panel). This genetic manipulation forces the rerouting of all glycolytic flux into the PPP and a lower growth rate (0.17–0.23 vs 0.83–0.9 h-1). 2S-^13^C MFA allows us to study the impact of this flux rerouting on the rest of the cell metabolism. For example, the overall NADPH demand falls three fold (vs a ∼ four fold decrease in growth rate). Furthermore, the demand is not equally met by the pentose phosphate pathway and the TCA cycle anymore: in the Δ*pgi*, the PPP enzymes G6PDH2r and GND produce most of the NADPH (81%) with a much less important role played by the TCA cycle (13–19%). Interestingly, the absolute flux through the PPP for the *pgi* KO and the wild type is very similar. NADH is generated mostly by the glyceraldehyde-3-phosphate (36–37%, GAPD), malate (10–27%, MDH), pyruvate (15–25%, PDH) and 2-Oxoglutarate dehydrogenases (7–11%, AKGDH), which provide 79–98% of all NADH. Consumption is dominated by the ubiquinone-8 NADH dehydrogenase (NADH6, 43–83%). After *pgi* is knocked out, NADH demand decreased ∼ 4 fold, with relative production by MDH undergoing the biggest drop (from 10–27% to 5–7%).

These results are consistent with previous ^13^C MFA calculations for NADPH production [68], which validates this approach. Furthermore, they also provide information on secondary metabolism and NADH balances in a systematic manner. A similar analysis can be done for acetyl-CoA, a key metabolite and common bottleneck in metabolic engineering [69, 70] or any other relevant metabolite such as ATP, AMP, Coenzyme A or FADH, which would provide detailed information on the organism’s underlying physiology.

### Extracellular metabolite prediction

The 2S-^13^C MFA results pinpoint which (non measured) metabolites are expected to be detected in the extracellular medium based on the values of exchange fluxes as constrained by the ^13^C labeling experiments (S13 Fig). Knowledge and quantification of the full range of excreted metabolites (also known as exometabolome or metabolic footprint [71]) is desirable for a full understanding of the biochemical impact of the cell on its environment. For metabolic engineering purposes, this knowledge provides important clues as to how to close the carbon balance and whether toxic compounds are being produced in the fermentation. For human metabolism, better prediction of extracellular fluxes can yield improved metabolic predictions when integrated with physiologically-based pharmokinetic models [72].

The exchange fluxes predictions typically have large confidence intervals, but these intervals are much smaller than for FBA (see S13 Fig). Metabolites expected to be detected in the medium are those whose exchange fluxes have net positive maximum and minimum values for a period of time sufficiently long so as to reach detection limits. For the *E. coli* strains considered here, urea, glycolate (glyc, S14 Fig), fumarate (fum, S15 Fig) and acetaldehyde (acald) are the non-typical metabolites expected to be present in the medium (acetate is already measured). This prediction of atypical metabolites is of particular interest in light of the recent discovery of extended overflow metabolism [73]. A full prioritized list can be obtained from the exchange flux information and used to direct mass spectrometry, NMR or vibrational spectroscopy efforts to find the missing metabolites until carbon balance is met. For future improvement of intracellular metabolic flux predictions, constraints introduced by extracellular metabolite measurements are very effective and usually easy to measure, but it is necessary to know which metabolites to look for and 2S-^13^C MFA provides precisely that type of insights.

### Comparison with ^13^C MFA

2S-^13^C MFA produces nearly the same results as ^13^C MFA for central carbon metabolism (see e.g. S4 Fig and S18 Fig). This similarity is not surprising since 2S-^13^C MFA is designed to mimic ^13^C MFA for this part of metabolism (see “Limiting flux to core reactions” section). The only difference for the current data set can be found in the TCA cycle flux, as described below. These differences arise because genome-scale models account for fluxes to biomass in a more detailed and realistic manner and because they do not rule out unexpected metabolic routes compatible with the available data.

Flux through the TCA cycle is lower in the ^13^C MFA solution (S4 Fig vs S18 Fig) because of an inaccurate account of fluxes to biomass in the ^13^C MFA model. Specifically, the large fluxes draining acetyl-CoA into biomass and to the exterior of the cell as acetate (each a third of the pyruvate dehydrogenase flux, PDH) imposed in the original publication [47] have been overestimated in this particular case. While the typical biomass function used in ^13^C MFA involves a specific stoichiometry of central carbon intermediates converted directly to biomass, this is only an approximation since some of the metabolites required for biomass growth are not represented in the minimal network model and need to be substituted by their requirements in terms of intermediates present in the minimal network (acetyl-CoA, in this case). These effects require significant effort to be accurately incorporated into a small-scale model, but they are elegantly handled by the genome-scale model. In this case a large flux of acetyl-CoA to biomass is assumed in the ^13^C MFA solution, while the 2S-^13^C MFA solution determines the requirements on core metabolism by setting the biomass flux to the measured growth rate, and automatically determining the fluxes needed to provide the metabolites present in the biomass equation through the comprehensive stoichiometry network reflected in Eq 2. Hence, genome-scale models provide an automatic and detailed accounting of biomass requirements.

The difference in TCA cycle flux extends to the glyoxylate shunt in some cases in which the isocitrate lyase (ICL) is active for the 2S-^13^C MFA (S5 and S6 Figs) solution, but not in the ^13^C MFA solution due to a limited ^13^C MFA model. In the 2S-^13^C MFA solution, the objective function (Eq 1) becomes slightly lower by diverting flux into the ICL and then shuttling it out of the core set from glyoxylate (glx) into glycolate. This route is not included in the ^13^C MFA model and activating the ICL for this model would produce a large amount of flux through the malate synthase (MLS). This MLS flux would significantly deteriorate the fit to malate labeling, so the glyoxylate shunt remains inactive for ^13^C MFA case. The shuttling of glyoxylate into glycolate is unexpected and, in fact, could be the result of a numerical artifact since the only labeling data available in the full TCA cycle is that of malate (mal-L) and small errors in the labeling measurements (or their confidence intervals) for this metabolite can lead to erroneous solutions. The glyoxylate shunt is known to be inactive under the given conditions and one could use this information and constrain its flux to zero. However, we aim to produce a general method; of use under conditions where this information may not be available (e.g. exotic feeds or bioengineered cells). Hence, we decided not to constrain the glyoxylate flux in order to show how the genome-scale model constrained by measured data can produce testable and falsifiable consequences to detect this type of errors. In this case, the shuttling of glyoxylate through glycolate results in glycolate being exported out to the medium (S14 and S15 Figs) and detectable through (e.g.) MS methods. If no glycolate is found, that extracellular flux can be set to zero and the glyoxylate shunt flux will decrease to zero, so as not to deteriorate the fit of the malate labeling pattern. Alternatively, the availability of labeling patterns for additional metabolites (fumarate, fum and glyoxylate, glx) would confirm or deny the ICL activity. In this way, 2S-^13^C MFA can fruitfully use available data to suggest and test unexpected metabolic activity; such as the surprising heme degradation in cancer cells with a non-functional TCA cycle [26].

### Using 2S-^13^C MFA to test flux prediction algorithms

**Fig 9:**
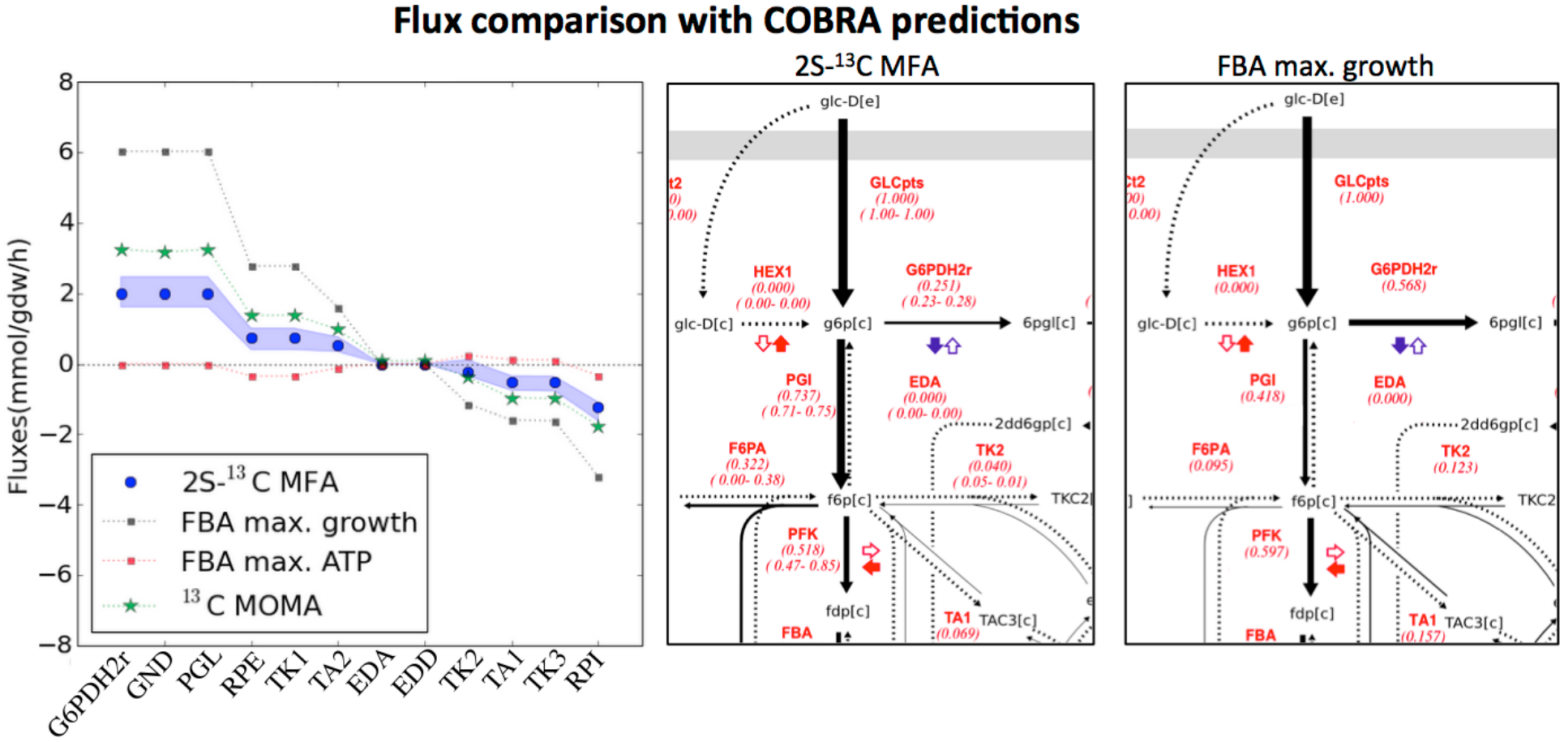
Flux comparison with COBRA predictions for *pyk* KO at 5 hours. The comparison of flux predictions through COBRA methods with fluxes measured through 2S-^13^C MFA shows how and why predictive methods fail. Left panel: Predicted fluxes for reactions in the pentose phosphate pathway (PPP) through FBA using maximum growth, FBA using maximum ATP production and ^13^C MOMA are compared with measurements through 2S-^13^C MFA (shading indicates confidence intervals). Fluxes predicted through maximum growth FBA offer a good qualitative description of fluxes but are quantitatively erroneous. ^13^C MOMA is a variation of MOMA that leverages 2S-^13^C MFA flux profiles and predicts fluxes more accurately. Center and right panels: Flux maps for two special cases (FBA and 2S-^13^C MFA). Maximum growth FBA overestimates flux into the PPP. Flux values are depicted in red: the upper value is the flux for the best fit and the lower values are the range of values compatible with data from ^13^C labeling experiments.

Our goal in developing 2S-^13^C MFA is to make a clear distinction between highly reliable constraints such as those induced by ^13^C labeling data, measured extracellular fluxes, carbon transition information and reaction stoichiometry, and reasonable hypotheses (such as growth rate optimization) that may not be universally applicable. 2S-^13^C MFA has been developed to provide a self-consistent and unbiased determination of the range of metabolic fluxes compatible with available experimental data. Unlike other COBRA methods, the fluxes obtained by 2S-^13^C MFA are backed up by the extra validation provided by the correct fit of 48 relative labeling measurements (see S1–S3 Figs). Hence, we use it here as a reference to compare various COBRA predictive methods based on different hypotheses. This procedure permits us to determine which method predicts fluxes most accurately. We compared flux profile predictions for *pyk* and *pgi* gene knock outs calculated at three different time points (5, 6 and 7 hrs for wild type and *pyk* KO, and 16, 21 and 23 hrs for *pgi* KO) using six different methods: FBA maximizing either growth and ATP production [74], Minimization of Metabolic Adjustment (MOMA [16]), Regulatory On/Off Minimization (ROOM [75]) and two new methods developed in this paper (Fig 9 and S19 and S20 Figs). These new methods are ^13^C MOMA and ^13^C ROOM, which leverage 2S-^13^C MFA flux profiles obtained for the wild type strain to improve flux predictions (see S3 Text), similar in spirit to the approach by Kuepfer *et al* [39].

Growth maximizing FBA provides predictions that are not quantitatively accurate, but seem approximately right for most cases (S19 and S20 Figs). This fact probably explains its success in metabolic engineering applications. Since FBA is typically used to make partial flux predictions by using some measured flux information for the predicted experiment, we tested this variation as well. Once fluxes for growth rate and excreted metabolites were constrained to the measured values, *pgi* predictions became extremely accurate (S20 Fig). However, when a full prediction (i.e., not using any data from the predicted experiment) was sought, FBA noticeably failed for this KO due to its inability to predict the drop in growth rate. ATP maximizing FBA fails most noticeably for the *pyk* KO, probably because the PYK reaction is involved in ATP production and its elimination significantly changes the ATP balance when ATP is to be maximized.

MOMA, ROOM, ^13^C MOMA and ^13^C ROOM flux predictions fail most blatantly for the *pgi* knockout strain because growth rates change radically and the method tries to maintain the previous flux levels (S19 Fig). Hence, we can expect these methods to offer good results only when changes expected in glucose intake and growth rates are relatively small. An improvement of these methods could be obtained if only the relative flux profiles are used for the prediction in the algorithm and the growth rate is separately obtained.

Since ^13^C MOMA and ^13^C ROOM use 2S-^13^C MFA flux profiles from the wild type strain, we expected that they would more accurately predict fluxes than would MOMA and ROOM. We find that is the case for fluxes in glycolysis and PPP, but the TCA cycle flux values are less accurate (see S19 Fig). This phenomena is most likely due to our initial flux profile not being very accurate for the TCA cycle (large confidence intervals, see S4–S6 Figs) since many fewer labeling measurements are available for TCA metabolites (only malate vs eight metabolites available for glycolysis and PPP). Labeling data for more metabolites next to branch points (e.g. fumarate, glyoxylate, isocitrate, alphaketoglutarate) would help reduce the flux confidence intervals for this area of metabolism.

These comparisons illustrate how 2S-^13^C MFA can be used to test COBRA methods, see where they fail and why, and use this information to improve them.

### Quantitative prediction of direct metabolite labeling measurements

The combination of ^13^C labeling data and genome-scale models with COBRA prediction methods allows us to make some predictions we cannot do using either ^13^C MFA or FBA. The ability to make accurate predictions of metabolism is of fundamental importance to make metabolic engineering a more predictive discipline.

**Fig 10:**
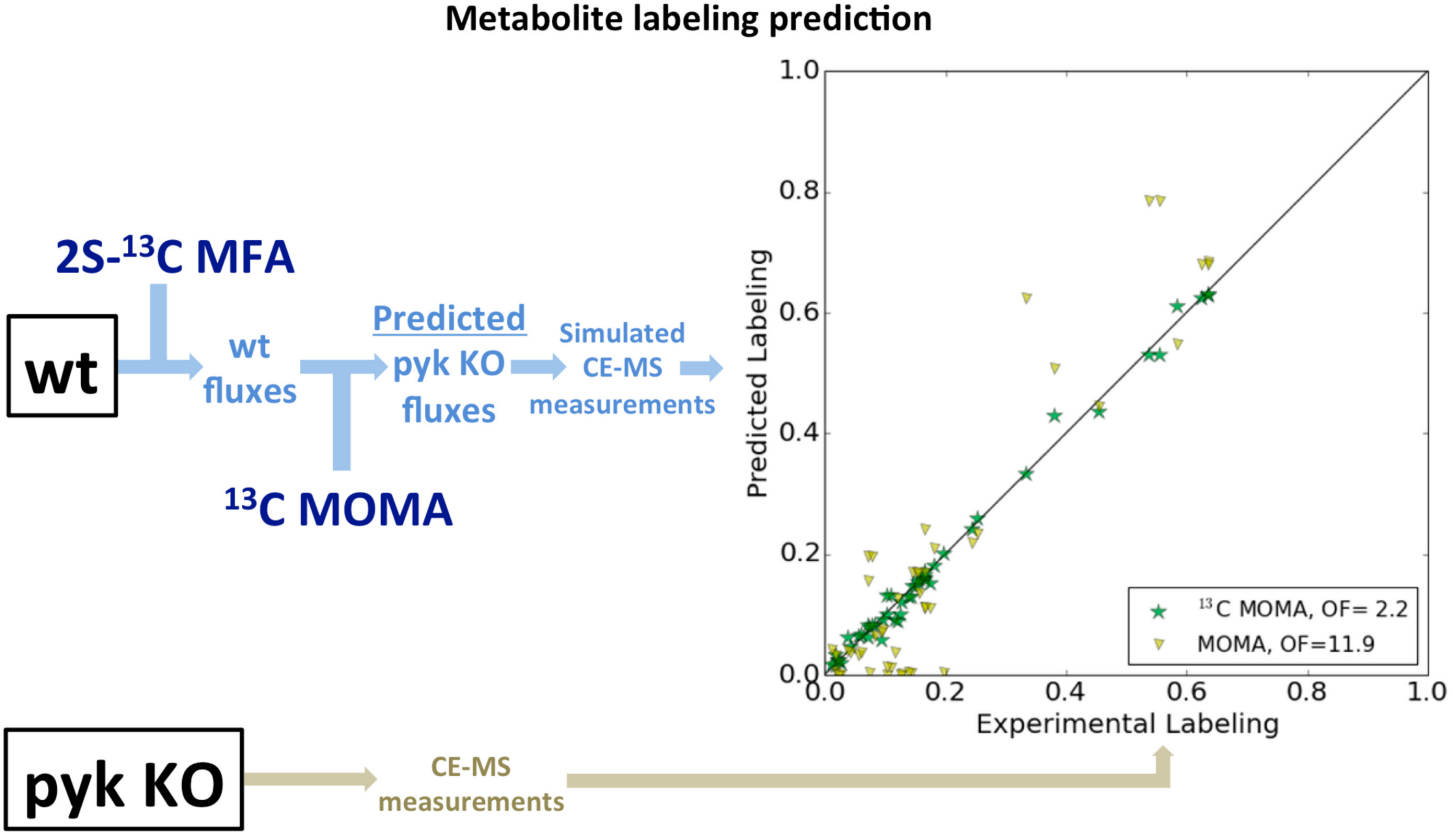
Metabolite Labeling prediction. The combination of ^13^C labeling data and genome-scale models with COBRA prediction methods produces predictions that cannot be obtained through either ^13^C MFA or FBA. The ^13^C MOMA predictions of fluxes shown in Fig 9 and S19 Fig are accurate enough that they can be used to predict the metabolite labeling (MDV, see Fig 4) for the *pyk* strain at 5 hours without using any data from the experiment on the *pyk* strain. A genome-scale model is needed to use ^13^C MOMA, and the accuracy provided by the ^13^C data is necessary to produce an accurate initial flux profile for ^13^C MOMA (see S2 Text). OF denotes the objective function: the average deviation of predicted labeling from the experimental value, measured in units of the experimental error. The prediction though standard MOMA, based on a FBA flux profile, is much less accurate (OF = 11.9) than the one obtained through ^13^C MOMA (OF = 2.2).

The ^13^C MOMA predictions of glycolysis and PPP fluxes for the *pyk* KO at the 5-hr time point surpass those of all other methods (Fig 9 and S19 Fig). In fact, the flux predictions are precise enough that we can even predict with reasonable accuracy the metabolite labeling to be expected from that strain at that time point (Fig 10). This is a prediction of directly measured data instead of a derived measurement such as flux. No information from the *pyk* strain at 5 hrs was used. We used the 2S-^13^C MFA flux calculations from the wild type strain at 5 hrs to obtain fluxes compatible with those measurements and then, through ^13^C MOMA, obtain the predicted fluxes if *pyk* were to be knocked out. The corresponding labeling patterns were then derived. Notice that this is very different from the partial predictions of labeling that ^13^C MFA routinely produces, where the labeling for the present experiment is predicted. Hence, this constitutes a full quantitative prediction of metabolite labeling from a ^13^C labeling experiment, which has not been reported before, to our knowledge.

The prediction of metabolite labeling is particularly relevant as validation because they refer to a directly measured quantity (i.e. the MDV obtained from the CE-TOFMS). Fluxes are derived quantities relying on a variety of implicit assumptions (i.e. the two-scale approximation, metabolic pseudo-steady state, no accumulation of intermediate metabolites, genome-scale model completeness and accuracy, cell homogeneity…). The real test that these assumptions are not severely violated and that the method provides reliable flux profiles is to use them to predict directly measured quantities for other experiments (in this case, labeling patterns). Moreover, this example shows that coupling ^13^C labeling data with COBRA methods opens the possibility to go beyond qualitative predictions (e.g. grow/no grow).

### Conclusion

In this manuscript, we have shown how to maximize the information obtained from ^13^C data to constrain genome-scale models, and that once core metabolism is set by ^13^C labeling data information, the rest of metabolism is generally highly constricted. As is a usual behavior in “sloppy” nonlinear fitting problems [40], some fluxes are very effectively constrained and some others only loosely. Confidence intervals obtained through 2S-^13^C MFA immediately identify these two types of fluxes and show that the use of ^13^C labeling experimental data produce much narrower confidence intervals than those produced by FBA. The method is generally applicable to any genome-scale model or feed, and can be used to expand the use of ^13^C-based flux analysis beyond the customary cases to tackle non-standard feeds, exotic organisms and systems described by large stochiometric matrices such as the human metabolic network [76], those derived from adding macromolecular synthesis [77] or microbial communities [24].

2S-^13^C MFA produces similar results as ^13^C MFA for the region where the latter is valid: central carbon metabolism. 2S-^13^C MFA, however, extrapolates the constraints induced by the ^13^C labeling data to a genome-scale model, providing fluxes not only central carbon metabolism but also for peripheral metabolism. This was illustrated firstly by a detailed description of NADPH and NADH production and consumption and, secondly, by predicting unmeasured metabolites expected in the extracellular medium.

2S-^13^C MFA does not use an evolutionary optimization principle (such as growth rate optimization) but, rather characterizes all flux profiles compatible with the experimental data. The extra validation gained by matching the measured labeling values is used to test the validity of the maximization hypothesis by comparing these results with predictions obtained through FBA and other COBRA methods. The comparison of flux profiles predicted with COBRA methods and those obtained through 2S-^13^C MFA provided not only a ranking of accuracy for predictions but also insight as to how to improve predictive methods. An improved version of MOMA using 2S-^13^C MFA profiles as a starting point is able to predict the outcome of 48 direct measurements of metabolite labeling for a *pyk* KO experiment using only data from a different experiment involving the wild type. This capability shows that using ^13^C labeling experimental data enables accurate predictions beyond qualitative cases (e.g. grow/no grow). This method represents another step in the effort to make bioengineering a more predictable endeavour.

The new method hinges on a simple assumption: flux flows from the core set to peripheral metabolism and does not flow back. This assumption is supported by the good fits obtained in general by ^13^C MFA methods thus far. There might be situations where this two-scale assumption is not applicable and these will be pinpointed by unacceptable fits to the labeling data. The core set of reactions, however, is flexible and can be enlarged as needed to provide acceptable fits to the labeling data. Hence, phenomena such as protein turnover [48, 78] or cell scavenging in stationary phase can be included by adding the appropiate reactions to the core. The availability of carbon transitions for genome-scale models [79] facilitates a systematic core enlargement.

In spite of its simplicity, FBA-based modelling has already exhibited significant success: only three elements are used in this modelling scheme (genome-scale stochiometry, measured extracellular fluxes and an optimization principle) but they have been successfully used to rationally engineer strains used for large-scale industrial production [21–23]. However, certainly not every single flux in a flux profile obtained through FBA can be trusted. 2S-^13^C MFA unites the informative constraints of ^13^C labeling experiments with genome-scale stoichiometry to improve the determination of internal metabolic fluxes and set confidence intervals based on experimental data. 2S-^13^C MFA completes and improves ^13^C MFA by enforcing a global balance of metabolites instead of balancing only a few chosen metabolites. We believe it will be a tool of extreme utility in bioengineering, at a time when a variety of different frameworks for flux prediction for genome-scale models are becoming available [80, 81]. Furthermore, we think that its widespread use to determine metabolic fluxes will affect our understanding of fundamental biological problems [82] beyond bioengineering.

## Materials and Methods

Mathematical details for the optimization problems in each of the different phases depicted in Fig 2 can be found below. The full procedure was scripted in python and uses the CONOPT solver version 3.15D to solve the nonlinear problems and CPLEX version 12.4.0 for the linear programming problems within the GAMS modeling environment.

All experimental data were obtained from Toya *et al* [47]. This study includes intracellular metabolite labeling, incoming and outgoing extracellular fluxes for glucose, acetate and growth rate for three strains of *E. coli* (wild type BW25113 and *p*gi and *p*yk knockouts) at three different time points each (5, 6 and 7 hrs for wild type and *pyk* KO, and 16, 21 and 23 hrs for *pgi KO*). Measured metabolites are 3-phospho-D-glycerate (3pg), dihydroxyacetone phosphate (dhap), D-fructose 1,6-bisphosphate (fdp), L-malate (mal-L), phosphoenolpyruvate (pep), pyruvate (pyr), alpha-D-Ribose 5-phosphate (r5p), ribulose 5-phosphate (ru5p) and Sedoheptulose 7-phosphate (s7p). Throughout the text, metabolites and reactions are named according to the iJR904 model notation [57, 83](bigg.ucsd.edu). The comparison with ^13^C MFA was done through the use of equations 4–9 in S1 Text. Equations for ROOM and MOMA can be found in S2 Text. While the iJR904 model was used in this manuscript, the method is equally applicable to iAF1260 or iJO1366. iJR904 was used because the compartimentalization in iAF1260 and iJO1366 (e.g. transport to periplasm) complicate the recursive procedure to generate a core set, without obviously improving the fit to the data for this case.

Protein turnover reactions were not included in the core set of reactions since it has been shown that protein degradation effects on intracellular metabolites are negligible once the labeling has reached steady state [84], which is the case for the current data set (see Fig 1 in [47]).

### Limiting flux to core

The two-scale approximation assumes that non-core reactions do not contribute directly to the labeling of core metabolites, since carbon precursors flow from core metabolism into peripheral metabolism and do not flow back. This is represented in terms of a genome-scale model by limiting to zero the flux of reactions flowing into core metabolism (see Algorithm 1). The first step in 2S-^13^C MFA (Fig 2) hence consists in taking each reaction that has a product in core metabolism and setting the upper bound to zero. However, it may be the case that this extra constraint makes it impossible to meet the measured growth rate (we check this by solving the corresponding FBA problem). In that case, setting the upper bound to a fraction of the glucose uptake rate is tested (first 0.05 and then 0.2 for this case). Since the labeling of core metabolism can be impacted by reversible reactions with reactants included in the core set as well, we cover this case by limiting the *lower* bound of the reaction to zero or the lowest value that permits growth. The impact of the reactions that could not be set to zero will be checked later through External Labeling Variability Analysis (ELVA, Figs 2 and 4). The input for the first step (Fig 2) is the genome-scale model with the carbon transitions for the core set integrated in it. The output consists of the genome-scale model with lower and upper bounds modified by this “Limiting flux to core” procedure. A detailed description of this first step in the diagram shown in Fig 2 can be found in the pseudo code in Algorithm 1.

**Algorithm 1. “Limiting flux to core” pseudo code**

for each reaction j flowing into core:

limits = [0,0.05,0.2]*glucose_uptake

limit = limits.next()

goOn = True

while goOn:

if reaction j has forward flux:

ub[j] = min(ub[j],limit)

else if reaction j has backward flux:

lb[j] = sign(lb[j])*min(abs(lb[j]),limit)

solve FBA problem

goOn = (FBA problem has no solution) and (limit is not the last value in limits)

limit = limits.next()

Where limits.next() obtains next value in list limits (the first one if uninitiated), and glucose_uptake is the value of the glucose uptake rate. Solve FBA problem refers to finding the solution to the problem given by equations 1–3 in S1 Text. has forward flux refers to the reaction having a possible positive flux (i.e. positive upper bound, ub) flowing into the core set and has backward flux refers to the reaction having a possible negative flux (i.e. negative lower bound, lb) flowing into the core set for reversible reactions. sign(lb[j]) is the sign of the lower bound lb[j]. ub[j] and lb[j] denote upper and lower bounds for reaction j, respectively.

### 2S-^13^C MFA data fit

The second step in 2S-^13^C MFA (Fig 2) involves fitting the measured metabolite labeling by solving the optimization problem in Eqs 1–7, where the upper (*ub*_*j*_) and lower bounds (*lb*_*j*_) have been limited by the previous step (“Limiting flux to core”). 2S-^13^C MFA is a hybrid of FBA and ^13^C MFA (see Fig 1 and S1 Text) where the stoichiometry constraint is applied to the full genome-scale network, as in the case of FBA, and the labeling constraints [58] are applied only to the core set of reactions and metabolites, as is the case for ^13^C MFA.

Unlike previous efforts [36, 39], these constraints are enforced simultaneously, instead of sequentially using the results of ^13^C MFA to constrain the FBA problem. This simultaneous approach is more rigorous than using slack coefficients (*δ* in Kuepfer *et al* [39]), does not need to invoke an optimization principle to calculate fluxes and allows for the global metabolite balance to affect core metabolism fluxes. Furthermore, it is also self-consistent whereas in the sequential approach one might find that fluxes that flow into core metabolism are active, even though they were not taken into account to do the initial ^13^C MFA fit.

In the notation of Suthers *et al* [58] (GAMS files available in S1 Code):

$$\begin{array}{ccc} \text{Minimize } & OF=\sqrt{\left(\sum_{\begin{array}{c} e\in E_{meas}\\ m\in M_{e} \end{array}}{\left(\frac{f_{em}^{exp}-f_{em}}{\Delta_{em}}\right)}^{2}/\left|M_{e}\right|\right)/\left|E_{meas}\right|} & \end{array}$$ (1)

Subject to:

$$\begin{array}{ccc} & \sum_{j}S_{ij}v_{j}=0 & \forall i\in I^{N},j\in J \end{array}$$ (2)

$$\begin{array}{ccc} & lb_{j}\leq v_{j}\leq ub_{j} & \forall j\in J \end{array}$$ (3)

$$\begin{array}{ccc} & \sum_{m\in M_{e}}f_{em}=1 & \forall e\in E_{co} \end{array}$$ (4)

$$\begin{array}{r} \Sigma_{e^{\prime }\in E_{co}}\left(\left(\Sigma_{l|EMM_{e^{\prime }->e}^{l}>0}EMM_{e^{\prime }->e}^{l}V_{l}\right)f_{e^{\prime }m}\right)+\left(\Sigma_{l|S_{il}^{*}<0}S_{il}^{*}V_{l}\right)f_{em}=0\\ \forall m\in M_{e},e\in E_{i},i\in I_{co}^{N} \end{array}$$ (5)

$$\begin{array}{ccc} & f_{em}=\sum_{w\in W_{em}}\prod_{n=1}^{|E_{e}|}f_{e_{n}m_{n}} & \forall m\in M_{e},e\in E_{co}^{c} \end{array}$$ (6)

$$\begin{array}{ccc} & v_{j}=\sum_{l\in J_{co}^{B}}map_{jl}\;V_{l} & \forall j\in J \end{array}$$ (7)

where:

$$\begin{array}{cc} \underline{Sets} & \\ \;I\equiv \{i\} & :\text{Set}\;\text{of}\;\text{all}\;\text{metabolites.}\\ \;I_{co}\subset I & :\text{Set}\;\text{of}\;\text{core}\;\text{metabolites.}\\ \;I_{co}^{N}\subset I_{co} & :\text{Set}\;\text{of}\;\text{non-exchange}\;\text{core}\;\text{metabolites.}\\ \;J & :\text{Set}\;\text{of}\;\text{fluxes.}\\ \;J_{co}\subset J & :\text{Set}\;\text{of}\;\text{core}\;\text{fluxes.}\\ \;J^{B} & :\text{Set}\;\text{of}\;\text{fluxes}\;\text{with}\;\text{backward}\;\text{and}\;\text{forward}\;\text{fluxes}\;\text{differentiated,}\\ & \;\text{e.g. PGI\_f, PGI\_b, PGL}\ldots \;\text{etc.}\\ \;J_{co}^{B}\subset J^{B} & :\text{Set}\;\text{of}\;\text{core}\;\text{fluxes}\;\text{for}\;J^{B}\;\text{.}\\ \;E=\{e\} & :\text{Elementary}\;\text{Metabolite}\;\text{Units}\;\text{(EMUs).}\\ \;E^{c}\subset E & :\text{Combined}\;\text{EMUs.}\\ \;E_{i}\subset E & :\text{EMUs}\;\text{from}\;\text{metabolite}\;i\in I\text{.}\\ \;E_{co}^{c}\subset E^{c} & :\text{Core}\;\text{combined}\;\text{EMUs.}\\ \;E_{e}\subset E & :\text{EMUs}\;\text{that}\;\text{produce}\;\text{combined}\;\text{EMU}\;e\text{.}\\ \;E_{co}\subset E & :\text{EMUs}\;\text{corresponding}\;\text{to}\;\text{core}\;\text{metabolites.}\\ \;E_{meas}\subset E & :\text{EMUs}\;\text{corresponding}\;\text{to}\;\text{measured}\;\text{EMUs.}\\ \;W_{em} & :\text{Set}\;\text{of}\;\text{every}\;\text{possible}\;\text{mass}\;\text{isotopomer}\;\text{multiplet}\;\text{of}\;E_{e}\;\text{that}\;\text{produce}\\ & \;\text{the}\;\text{mass}\;\text{isotopomer}\;m\;\text{of}\;e\text{.}\\ \;M_{e} & :m\;\text{values}\;\text{for}\;\text{MDV}\;\text{of}\;\text{emu}\;e:0,1,\cdots ,\text{\# of carbons in}\;e.\\ \underline{Parameters} & \\ \;EMM_{e^{\prime }->e}^{l} & =\frac{1}{k}\text{if}\;e^{\prime }\text{produces}\;e\;\text{through reaction}\;l\in J_{co}^{B},0\;\text{otherwise}.\text{See Suthers}\;et\;al[58].\\ \;S_{ij} & :\text{Stoichiometry}\;\text{matrix.}\\ \;S_{il}^{*} & :\text{Stoichiometry}\;\text{matrix}\;\text{with}\;\text{backward}\;\text{and}\;\text{forward}\;\text{fluxes}\;\text{differentiated.}\\ \;ub_{j},lb_{j} & :\text{Upper}\;\text{and}\;\text{lower}\;\text{bounds}\;\text{for}\;\text{reaction}\;j\text{.}\\ \;f_{em}^{exp}\in \left[0,1\right] & :\text{Experimentally}\;\text{measured}\;\text{MDV}\;\text{for}\;\text{emu}\;e\;\text{from}\;\text{metabolite}\;m\text{.}\\ \;\Delta_{em} & :\text{Measurement}\;\text{error}\;\text{for}\;f_{em}^{exp}\text{.}\\ \;map_{jl} & =1*glucupt\;\text{if}\;l\;\text{corresponds to forward flux of}\;j.\\ & =-1*glucupt\;\text{if}l\;\text{corresponds to backward flux of}\;j.\\ \underline{Variables} & \\ \;v_{j} & :\text{Flux}\;\text{value}\;\text{of}\;\text{reaction}\;j\in J\text{,}\;\text{in}\;\text{mmol/gdw/h.}\\ \;V_{l} & :\text{Flux}\;\text{value}\;\text{of}\;\text{reaction}\;l\in J_{co}^{B}\text{,}\;\text{normalized}\;\text{to}\;\text{glucose}\;\text{input}\;\text{rate.}\\ \;f_{em}\in \left[0,1\right] & :\text{Mass}\;\text{isotopomer}\;\text{fraction}\;\text{(MDV)}\;\text{for}\;\text{emu}\;e\;\text{from}\;\text{metabolite}\;m\text{.} \end{array}$$

Notice that $S_{ij}^{*}$ is not the same as *S*_*ij*_, since *J* and *J* ^*B*^ are slightly different sets of fluxes. In fact:

$$\begin{array}{cc} S_{il}^{*}=S_{ij} & \text{if}\;l\;\text{is}\;\text{the}\;\text{forward}\;\text{version}\;\text{of}j.\\ S_{il}^{*}=-S_{ij} & \;\text{if}\;l\text{is}\;\text{the}\;\text{backward}\;\text{version}\;\text{of}j. \end{array}$$ (8)

Notice that Eqs 2 and 3 are the traditional FBA contraints and that Eqs 4–6 are the ^13^C labeling constraints, but they have been limited to core metabolites and reactions. The mapping (Eq 7) converts the fluxes from the ^13^C MFA description (higher resolution) where fluxes are normalized to the glucose uptake rate (*glucupt*) into the FBA description (lower resolution). We describe the ^13^C MFA description as of higher resolution because the ^13^C labeling data can pick up differences in forward and backward fluxes (*J* ^*B*^ set), whereas the purely stochiometric approach of FBA can only constrain net fluxes (*J* set).

For all 2S-^13^C MFA calculations all input flux was supposed to be routed through GLCpts (glucose transport via PEP:pyr pts [57]). Optimization problems were run *N* = 30 times and the one with lowest objective function picked. The value of *N* was chosen so as to avoid relative minima of OF. A plot of how the OF saturated as *N* increased can be found in S21 Fig in the supplementary material.

In addition to the OF, the more typical sum-of-squares residuals (SSR) for the fits have been included in the legends of S1–S3 Figs. However, standard good-of-fitness metrics, such as those proposed by Antoniewicz *et al* [85], are not applicable to 2S-^13^C MFA. By using genome-scale models, the number of estimated free fluxes (*p*) is higher than the number of independent measurements (*n* = 48 − 9 = 39 in this case) and the *χ* ^2^(*n* − *p*) distribution of a negative number of degrees of freedom is then not properly defined. This apparent paradox arises because of the implicit assumptions in the *χ* ^2^ statistics approach to these types of fits. The null model assumes that each of the terms in the SSR are independent random variables, hence the degrees of freedom are the number of terms in the SSR [86]. Nonetheless, we know that, for ^13^C MFA, the terms in the SSR are typically not independent: the labeling pattern (MDV) of related compounds (i.e. amino acids arising from the same precursors) are very similar. Hence, Antoniewicz *et al* [85], decided to use as degrees of freedom the number of individual measurements minus the number of estimated free fluxes. This proposal seems reasonable for standard ^13^C MFA, but breaks down for genome-scale models with a much larger number of degrees of freedom.

One could choose a different null model for the *χ* ^2^ statistics (and previous approaches have shown that the standard *χ* ^2^ goodness-of-fit approach is probably too conservative, see Fig 3A in [27]) but the crux of the matter is that we believe that using p-values < 0.05 as an absolute arbitrary threshold for significant vs insignificant results is too simplistic, as do other biological researchers [87] or R.A. Fisher himself [88]. Hence, what we report here (in S1–S3 Figs) is what we believe is a better (and more intuitive) way to estimate how good a fit is: the average objective function normalized to the measurement error (Eq 1). This is the answer to the question: how different are my fits from the experimentally measured values, measured in units of the experimental error? This is in accordance in spirit with the suggestion of presenting measures of significance without arbitrary thresholds [87]. Notice that none of the objective functions (OF) in S1–S3 Figs is smaller than one, indicating that the difference between experiment and theory cannot be explained through experimental error. This may be because the experimental error for the labeling pattern was underestimated or because the model fails to explain the full labeling pattern. These results are in line with the general trend that fits to intracellular metabolites tend to be worse [89] than fits to proteogenic amino acids [49].

The discarding of the 0.05 p-value criterium does not imply that the considerable effort employed in obtaining excellent fits to data [49, 51] goes unrewarded. Under the 2S-^13^C MFA method, a bad fit to the experimental data results in a larger value for *δ*_*e*_ *m* in Eq 23 and wider confidence intervals. Hence, worse fits beget less flux resolution.

Finally, we think that the best validation of the of a flux fit is using the flux distribution to predict the results of another experiment, as we did in Figs 9 and 10.

### External Labeling Variability Analysis (ELVA)

Once a core set is chosen, ELVA establishes the maximum impact of non-core metabolism in the labeling of the measured metabolites. In order to do so, only core metabolism is considered and the impact of non-core metabolism is represented through “inflow” metabolites and reactions (see Fig 3C for an example). Inflow reactions agglomerate all non-core reactions flowing into (or out of) a particular core metabolite (see S22 Fig in supplementary material) and are assigned trivial carbon transitions (e.g. abc --> abc for a three carbon metabolite). Inflow metabolites are dummy metabolites with the same number of carbons as the involved core metabolite. ELVA constrains fluxes to the solution obtained in the “Fit data” step (Eq 10) and maximizes and minimizes the computational MDV for each *m* in each of the labeled metabolites. Since fluxes are fixed for all reactions and labeling is fixed for all metabolites except inflow metabolites, the optimization problem in Eqs 9–15 quantifies the maximum and minimum effect that this unknown labeling (since it comes from non-core metabolism) could have on the labeling pattern for the measured metabolites (*f*_*em*_∀*m*,*e* ∈ *E*_*meas*_):

$$\begin{array}{ccc} \text{Min/max}\; & f_{em} & \forall m\in M_{e},e\in E_{meas} \end{array}$$ (9)

Subject to:

$$\begin{array}{ccc} & V_{j}={\bar{V}}_{j} & j\in J_{coext}^{B} \end{array}$$ (10)

$$\begin{array}{ccc} & \sum_{j}S_{ij}^{*}V_{j}=0 & \forall i\in I_{coext}^{N},j\in J_{coext}^{B} \end{array}$$ (11)

$$\begin{array}{ccc} & lb_{j}\leq V_{j}\leq ub_{j} & \forall j\in J_{coext}^{B} \end{array}$$ (12)

$$\begin{array}{ccc} & \sum_{m\in M_{e}}f_{em}=1 & \forall e\in E \end{array}$$ (13)

$$\begin{array}{r} \sum_{e^{\prime }\in E}\left(\left(\sum_{j|EMM_{e^{\prime }->e}^{j}>0}EMM_{e^{\prime }->e}^{j}V_{j}\right)f_{e^{\prime }m}\right)+\left(\sum_{j|S_{ij}^{*}<0}S_{ij}^{*}V_{j}\right)f_{em}=0\\ \forall m\in M_{e},e\in E_{i},i\in I^{N} \end{array}$$ (14)

$$\begin{array}{ccc} & f_{em}=\sum_{w\in W_{em}}\prod_{n=1}^{|E_{e}|}f_{e_{n}m_{n}} & \forall m\in M_{e},e\in E_{coext}^{c} \end{array}$$ (15)

where symbols are as explained before, with the addition of:

$$\begin{array}{cc} \underline{Sets} & \\ \;I_{coext}^{B} & :\text{Set}\;\text{of}\;\text{extended}\;\text{metabolites.}\\ \;J_{coext}^{B} & :\text{Set}\;\text{of}\;\text{extended}\;\text{fluxes}\;\text{with}\;\text{backward}\;\text{and}\;\text{forward}\;\text{fluxes}\;\text{differentiated.}\\ \underline{Parameters} & \\ \;{\bar{V}}_{l} & :\text{solutions}\;\text{to}\;\text{the}\;\text{problem}\;\text{given}\;\text{by}\;\text{equations}\;1-7 \end{array}$$

$J_{coext}^{B}$ and *I*_*coext*_ are the set of reactions and metabolites obtained after expanding the core set to meet stoichiometry requirements (Eq 11) as is explained in supplementary S22 Fig. The input for this step is the flux profile obtained from the data fit and the output is an ELVA plot (Fig 4) used to decide whether the solution is self-consistent or not.

### ^13^C flux variability analysis

Once a self-consistent core set has been determined through the recursive procedure in Fig 2, flux ranges compatible with the experimental data are obtained through the following optimization problem:

$$\begin{array}{ccc} \text{Min/max} & V_{j} & \forall j\in J \end{array}$$ (16)

Subject to:

$$\begin{array}{ccc} & \sum_{j}S_{ij}v_{j}=0 & \forall i\in I^{N},j\in J \end{array}$$ (17)

$$\begin{array}{ccc} & lb_{j}\leq v_{j}\leq ub_{j} & \forall j\in J \end{array}$$ (18)

$$\begin{array}{ccc} & \sum_{m\in M_{e}}f_{em}=1 & \forall e\in E_{co} \end{array}$$ (19)

$$\begin{array}{r} \Sigma_{e^{\prime }\in E_{co}}\left(\left(\Sigma_{l|EMM_{e^{\prime }->e}^{l}>0}EMM_{e^{\prime }->e}^{l}V_{l}\right)f_{e^{\prime }m}\right)+\left(\Sigma_{l|S_{il}^{*}<0}S_{il}^{*}V_{l}\right)f_{em}=0\\ \forall m\in M_{e},e\in E_{i},i\in I_{co}^{N} \end{array}$$ (20)

$$\begin{array}{ccc} & f_{em}=\sum_{w\in W_{em}}\prod_{n=1}^{|E_{e}|}f_{e_{n}m_{n}} & \forall m\in M_{e},e\in E^{c} \end{array}$$ (21)

$$\begin{array}{ccc} & v_{j}=\sum_{l\in J_{co}^{B}}\;map_{jl}V_{l} & \forall j\in J \end{array}$$ (22)

$$\begin{array}{ccc} & {\left(f_{em}-f_{em}^{exp}\right)}^{2}\leq \delta_{em}^{2} & \;\forall e\in E_{meas},m\in M_{e} \end{array}$$ (23)

where symbols are as explained before, with the addition of:

$$\begin{array}{cc} \underline{Parameters} & \\ \;\delta_{em} & :\;\text{Maximum error allowed for}\;f_{em}.\\ & \;\delta_{em}=\Delta_{em}\;\text{if}\;\Delta_{em}>\epsilon_{em}\;\text{and}\;\delta_{em}=1.1*\epsilon_{em}\;\text{if}\;\Delta_{em}<=\epsilon_{em}\\ & \;\text{where}\;\epsilon_{em}=f_{em}^{fit}-f_{em}^{exp}\;\text{from the solution to equations}\;1-7. \end{array}$$

The fluxes *v*_*j*_ and *V*_*j*_ are initialized to the values obtained from solving Eqs 1–7. The results of the minimization and maximization give the flux upper and lower bound compatible with the experimental data from the ^13^C labeling experiments. This procedure is similar to the *FluxRange* procedure in Suthers *et al* [27], with the exception that we use set constraints for each measured data point (Eq 23) instead of only for the objective function. This approach guarantees that fluxes produce labeling patterns within the experimental error in a much more efficient way than a monte carlo approach. For example, consider these reactions which conform a futile cycle:

$$\begin{array}{cccc} \text{NDPK}\;1: & gdp_{c}+atp_{c} & <=> & gtp_{c}+adp_{c}\\ \text{ADK}\;1: & amp_{c}+atp_{c} & <=> & 2.0\;adp_{c}\\ \text{ADK}\;2: & gtp_{c}+amp_{c} & <=> & gdp_{c}+adp_{c} \end{array}$$

and for which the ^13^C labeling data cannot constrain the flux values in any fashion. This is shown correctly in the confidence intervals obtained with the method expressed through Eqs 16–23:

$$\begin{array}{cccc} \text{NDPK}\;1: & [-497.4 & - & 500\;\;]\\ \text{ADK}\;1: & [-497.4 & - & 500\;\;]\\ \text{ADK}\;2: & [-498.0 & - & 499.3] \end{array}$$

whereas if we perform a Monte Carlo, where labeling data is randomly chosen within the experimental error (100 instances), we do not obtain the proper range:

$$\begin{array}{cccc} \text{NDPK}\;1: & [-490.8 & - & -489.7]\\ \text{ADK}\;1: & [\;500.0 & - & \;500.0]\\ \text{ADK}\;2: & [-498.0 & - & -498.0] \end{array}$$

The input for this step is the flux profile obtained in the “Fit data” step and the outputs are the flux profile with corresponding confidence intervals, as shown in S4–S12 Figs.

## Supporting Information

S1 Text FBA and ^13^C MFA equations. (PDF)

S2 Text ^13^C MOMA and ^13^C ROOM. (PDF)

S3 Text Reactions and carbon transitions. (PDF)

S1 Code GAMS files. GAMS files, input files and expected outputs for the nine strains considered (wild type at 5, 6 and 7 hrs, pyk KO at 5, 6 and 7 hrs and pgi KO at 16, 21 and 23 hrs) for the data fits.
(GZ) Click here for additional data file.

S1 Fig Labeling data fit for wild type strain. Red denotes the MDV for experimentally measured data, blue columns are the fit. The MDV is the fraction of molecules with *m* = 1,2,3,4… ^13^ *C* incorporated atoms. Sum of square residuals (SSR, [85]) are 216.5, 282.2 and 467.7 for each strain from top to bottom.
(TIF)

S2 Fig Labeling data fit for pyk KO. Red denotes the MDV for experimentally measured data, blue columns are the fit. SSRs are 773.2, 1195.9 and 817.36 for each strain from top to bottom.
(TIF)

S3 Fig Labeling data fit for pgi KO. Red denotes the MDV for experimentally measured data, blue columns are the fit. SSRs are 7244.8, 6377.4 and 581.3 for each strain from top to bottom.
(TIF)

S4 Fig 2S-^13^C MFA flux map for wild type at 5 hours. Best fit for flux is given on top red number for each reaction and confidence interval at the bottom. Cofactors and common metabolites are indicated by small arrows. Reversible reactions are indicated by double arrows.
(TIF)

S5 Fig 2S-^13^C MFA flux map for wild type at 6 hours. (TIF)

S6 Fig 2S-^13^C MFA flux map for wild type at 7 hours. (TIF)

S7 Fig 2S-^13^C MFA flux map for pyk KO at 5 hours. (TIF)

S8 Fig 2S-^13^C MFA flux map for pyk KO at 6 hours. (TIF)

S9 Fig 2S-^13^C MFA flux map for pyk KO at 7 hours. (TIF)

S10 Fig 2S-^13^C MFA flux map for pgi KO at 16 hours. (TIF)

S11 Fig 2S-^13^C MFA flux map for pgi KO at 21 hours. (TIF)

S12 Fig 2S-^13^C MFA flux map for pgi KO at 23 hours. (TIF)

S13 Fig Extracellular metabolite prediction. Maximum (dark bar) and minimum (light bar) values of the exchange fluxes obtained by 2S-^13^C MFA show how ^13^C experimental data can effectively constrain exchange fluxes that have not been measured (in blue). For comparison, maximum and minimum values for the model constrained by extracellular flux measurements (through FVA, in red) are included as well, as are maximum and minimum values obtained through FVA for a model constrained by extracellular flux measurements along with constraints induced by the two-scale approximation (black, see “Limiting flux to core” section). Fluxes are for the wild type at 5 hrs, and exchanged metabolites are indicated in the x axis (iJR904 notation), [57, 83]). A positive exchange flux (excreted metabolite) that remains positive for long enough should produce a detectable pool of the corresponding metabolite. Acetate and glucose are used as constraints for the flux determination, hence the confidence intervals are very narrow. For this particular case, glycolate and urea are expected in the media.
(TIF)

S14 Fig Extracellular flux prediction for *pyk* KO at 6 hours. Expected metabolites in the medium include alpha-Ketoglutarate (akg) and glycolate (glyclt).
(TIF)

S15 Fig Extracellular flux prediction for *pgi* KO at 21 hours. Expected metabolites in the medium include fumarate (fum) and acetaldehyde (acald).
(TIF)

S16 Fig Version of Fig 8 for glutatamate dehydrogenase (GLUDy) and isocitrate dehydrogenase (ICDHyr) reactions. Since the flux value for SUCD1i is negative, the absolute value has been plotted.
(TIF)

S17 Fig Comparison between original flux profiles for the *pgi* KO and flux profile after changing biomass composition according to previously reported results [90, 91]Changes in flux profiles in central carbon metabolism are minimal. The ^13^C labeling data constrains fluxes strongly to a particular solution whereas changes in biomass requirements can be easily accommodated by the increased degrees of freedom found in genome-scale models.
(TIF)

S18 Fig ^13^C MFA flux map for wild type at 5 hours. (TIF)

S19 Fig Flux profile comparison of full predictions with 2S-^13^C MFA. Full prediction means that no data from the target experiment was used to constrain fluxes: all predictions were derived from data from a different experiment. 2S-^13^C MFA profiles are found by solving Eqs 1–7. Maximum growth and ATP profiles are found by solving equations 1–3 in S1 Text. MOMA, ^13^C MOMA, ROOM and ^13^C ROOM flux profiles are obtained as explained in S3 Text. Fluxes are sorted according to the following order; (PPP): G6PDH2r, GND, PGL, RPE, TK1, TA2, EDA, EDD, TK2, TA1, TK3, RPI; (GG): GAPD, ENO, PDH, TPI, PGI, FBA, PFK, F6PA, GLCS1, GLGC, FBP, G1PP, GLCP, HEX1, PPS, PYK, PGM, PGK; (CAC): FUM, MDH, ACONT, CS, ICDHyr, SUCD1i, AKGDH, CITL, FRD2, FRD3, MDH2, MDH3, SUCOAS; (OUT): EX_ac(e), BiomassEcoli, EX_glc(e). All reaction names according to iJR906 [57].
(TIF)

S20 Fig Flux profile comparison of partial predictions with 2S-^13^C MFA. Partial prediction means data from the target experiment was used to constrain fluxes, in this case the values for the growth rate, glucose intake and acetate excretion rate. 2S-^13^C MFA profiles are found by solving Eqs 1–7 in the main paper. Maximum growth and ATP profiles are found by solving equations 1–3 in S1 Text. Transparencies indicate confidence intervals for the 2S-^13^C MFA.
(TIF)

S21 Fig Average objective Function (OF) scaling with number of processes run (*N*) for wild type at 5 hours. OF plateaus at *N* ≈ 15. We chose *N* = 30 for our simulations.
(TIF)

S22 Fig Obtaining the network for External Labeling Variablity Analysis (ELVA). The purpose of ELVA is to determine if the reactions left out of the core metabolism significantly affect core metabolite labeling. In order to do so, only the core metabolism network is used and non-core metabolism is represented through inflow reactions and metabolites. Inflow reactions and metabolites are dummy reactions and metabolites that aggregate the non-core effects. In the figure, black denotes core metabolites and reactions and blue denotes noncore metabolites and reactions. Inflow reactions and metabolites are added to the core set to meet stoichiometric requirements (since core fluxes are fixed to the values obtained in the previous “Fit data” step). For example, the upper figures show how reactions ARGSL and ADSL1r are combined into ROfum leading into the dummy metabolite OUTfum, while keeping the same net flux out of fumarate. In the case of the lower figures ARGSL and ADSL1r have a net flux into the core set and are substituted by a inward flowing dummy reaction RIfum and a dummy metabolite OUTfum. The point of the ELVA is to elucidate the impact of the metabolites in the noncore set (see materials and methods). Outflowing reactions have no effect (upper panels) but inflowing reactions do (lower panels). The labeling of incoming dummy metabolites is left unconstrained since its value is not being tracked and our goal is to determine what is the maximum effect they may have on the measured metabolite labeling.
(TIF)
