# Supplementary material for: A Method to Constrain Genome-Scale Models with 13C Labeling Data
Source: PLoS Comput Biol. 2015 Sep 17;11(9):e1004363. doi: 10.1371/journal.pcbi.1004363 (PMC4574858; doi:10.1371/journal.pcbi.1004363)
Supplement: S1 Text — (PDF) [file pcbi.1004363.s001.pdf]

# Text S1: Appendix A

"A method to constrain genome-scale models with $^{13}$C labeling data"

Héctor García Martín[1,2,*], Vinay Satish Kumar[1,2], Daniel Weaver[1,2], Amit Ghosh[1,2], Victor Chubukov[1,2], Aindrila Mukhopadhyay[1,2], Adam Arkin[1,3], Jay D. Keasling[1,2,3,4]

June 17, 2015

## 1 FBA and $^{13}$C MFA description

**FBA** can be mathematically expressed as the following linear programming problem[2]:

$$\text{Maximize} \quad v_{obj} \tag{1}$$

Subject to:

$$\sum_j S_{ij} v_j = 0 \qquad \forall i \in I^N, j \in J \tag{2}$$

$$lb_j \leq v_j \leq ub_j \qquad \forall j \in J \tag{3}$$

where:

*Sets*

| | |
|---|---|
| $I^N \subset I$ | : Set of non-exchange metabolites. |
| $J = \{j\}$ | : Set of fluxes. |

*Parameters*

| | |
|---|---|
| $S_{ij}$ | : Stoichiometry matrix. |
| $ub_i, lb_i$ | : Upper and lower bounds for reaction $i$. |

*Variables*

| | |
|---|---|
| $v_i$ | : Flux value of reaction $i$, in mmol/gdw/h. |

and *obj* is the objective flux, for maximum growth rate $obj = BiomassEcoli$ and for ATP maximization $obj = ATPM$[1].

$^{13}$**C MFA**, on the other hand, is expressed as a non-linear optimization problem[4]:

$$\text{Minimize } OF = \sqrt{\sum_{e \in E_{\text{meas}}} \left( \sum_{m \in M_e} \left( \frac{f_{em}^{exp} - f_{em}}{\Delta_{em}} \right)^2 \Big/ |M_e| \right) \Big/ |E_{\text{meas}}|} \quad (4)$$

Subject to:

$$\sum_j S_{ij}^* V_j = 0 \qquad \forall i \in I^N, j \in J^B \quad (5)$$

$$LB_j \leq V_j \leq UB_j \qquad \forall j \in J^B \quad (6)$$

$$\sum_{m \in M_e} f_{em} = 1 \qquad \forall e \in E \quad (7)$$

$$\sum_{e' \in E} \left( \left( \sum_{j | EMM_{e'->e}^j > 0} EMM_{e'->e}^j V_j \right) f_{e'm} \right) + \left( \sum_{j | S_{ij}^* < 0} S_{ij}^* V_j \right) f_{em} = 0$$
$$\forall m \in M_e, e \in E_i, i \in I^N \quad (8)$$

$$f_{em} = \sum_{w \in W_{em}} \prod_{n=1}^{|E_e|} f_{e_n m_n} \qquad \forall m \in M_e, e \in E^c \quad (9)$$

where:

<u>*Sets*</u>

| | | |
|---|---|---|
| $I \equiv \{i\}$ | : | Set of all metabolites. |
| $I^N \subset I$ | : | Set of non-exchange metabolites. |
| $J^B$ | : | Set of fluxes with backward and forward fluxes differentiated, e.g. PGI_f, PGI_b, PGL .... etc. |
| $E = \{e\}$ | : | Elementary Metabolite Units (EMUs). |
| $E^c \subset E$ | : | Combined EMUs. |
| $E_i \subset E$ | : | EMUs from metabolite $i \in I$. |
| $E_e \subset E$ | : | EMUs that produce combined EMU $e$. |
| $E_{meas} \subset E$ | : | EMUs corresponding to measured EMUs. |
| $W_{em}$ | : | Set of every possible mass isotopomer multiplet of $E_e$ that produce the mass isotopomer $m$ of $e$. |
| $M_e$ | : | $m$ values for MDV of emu $e : 0, 1, \ldots, \#$ of carbons in $e$ . |

<u>*Parameters*</u>

| | | |
|---|---|---|
| $EMM_{e'->e}^j$ | : | $= \frac{1}{k}$ if $e'$ produces $e$ through reaction $j \in J^B$, 0 otherwise. See below. |
| $S_{il}^*$ | : | Stoichiometry matrix with backward and forward fluxes differentiated. |
| $UB_j, LB_j$ | : | Upper and lower bounds for reaction $j$. |
| $f_{em}^{exp} \in [0,1]$ | : | Experimentally measured MDV($m$)[3] for emu $e$. |
| $\Delta_{em}$ | : | Measurement error for $f_{em}^{exp}$. |

<u>*Variables*</u>

| | | |
|---|---|---|
| $V_l$ | : | Flux value of reaction $i \in J^B$, normalized to glucose input rate. |
| $f_{em} \in [0,1]$ | : | MDV for emu $e$ from metabolite $m \in M_e$. |

$k = 1$ or 2. It will equal 2 if scrambling is involved and two labeling patterns are produced[4] (e.g. SUCOAS reaction, see Text S3). Notice, as well, that $S_{ij}^*$ is not the same as $S_{ij}$, since $J$ and $J^B$ are slightly different sets of fluxes. In fact:

$$\begin{array}{ll} S_{il}^* = S_{ij} & \text{if } l \text{ is the forward version of } j. \\ S_{il}^* = -S_{ij} & \text{if } l \text{ is the backward version of } j. \end{array} \quad (10)$$

# References


[1] Ramprasad Ramakrishna, Jeremy S. Edwards, Andrew McCulloch, and Bernhard O. Palsson. Flux-balance analysis of mitochondrial energy metabolism: consequences of systemic stoichiometric constraints. *Am J Physiol Regulatory Integrative Comp Physiol*, 280(3):R695–704, March 2001.

[2] Daniel Segrè, Dennis Vitkup, and George M Church. Analysis of optimality in natural and perturbed metabolic networks. *Proceedings of the National Academy of Sciences of the United States of America*, 99(23):15112–7, November 2002.

[3] Patrick F Suthers, Anthony P Burgard, Madhukar S Dasika, Farnaz Nowroozi, Stephen Van Dien, Jay D Keasling, and Costas D Maranas. Metabolic flux elucidation for large-scale models using 13C labeled isotopes. *Metabolic Engineering*, 9(5-6):387–405, 2007.

[4] Patrick F Suthers, Young J Chang, and Costas D Maranas. Improved computational performance of MFA using elementary metabolite units and flux coupling. *Metabolic Engineering*, pages 1–6, 2009.
