# Supplementary material for: A Method to Constrain Genome-Scale Models with 13C Labeling Data
Source: PLoS Comput Biol. 2015 Sep 17;11(9):e1004363. doi: 10.1371/journal.pcbi.1004363 (PMC4574858; doi:10.1371/journal.pcbi.1004363)
Supplement: S2 Text — (PDF) [file pcbi.1004363.s002.pdf]

Text S2: Appendix B

"A method to constrain genome-scale models with ^{13}C labeling data"

Héctor García Martín[1,2,*], Vinay Satish Kumar[1,2], Daniel Weaver[1,2],Amit Ghosh[1,2], Victor Chubukov[1,2], Aindrila Mukhopadhyay[1,2], Adam Arkin[1,3], Jay D. Keasling[1,2,3,4]

June 17, 2015

1 ^{13}C MOMA and ^{13}C ROOM

MOMA[1] and ROOM[2] were used to provide predictions for gene KOs as displayed in Figs. 10 and S17. Both MOMA and ROOM predict flux profiles for a genetic manipulation based on an initial flux profile obtained before the manipulation takes place. Typically this initial flux profile is obtained using FBA. Those predictions are labeled as MOMA and ROOM in Fig. S17. We created new versions of MOMA and ROOM that leverage the flux profiles from 2S-^{13}C MFA, which is expected to be more accurate since it is aditionally constrained by ^{13}C labeling data. Figure 1 shows the difference between 2S-^{13}CMFA-based and FBA-based MOMA. The mathematical representation of the optimization problems is shown below.

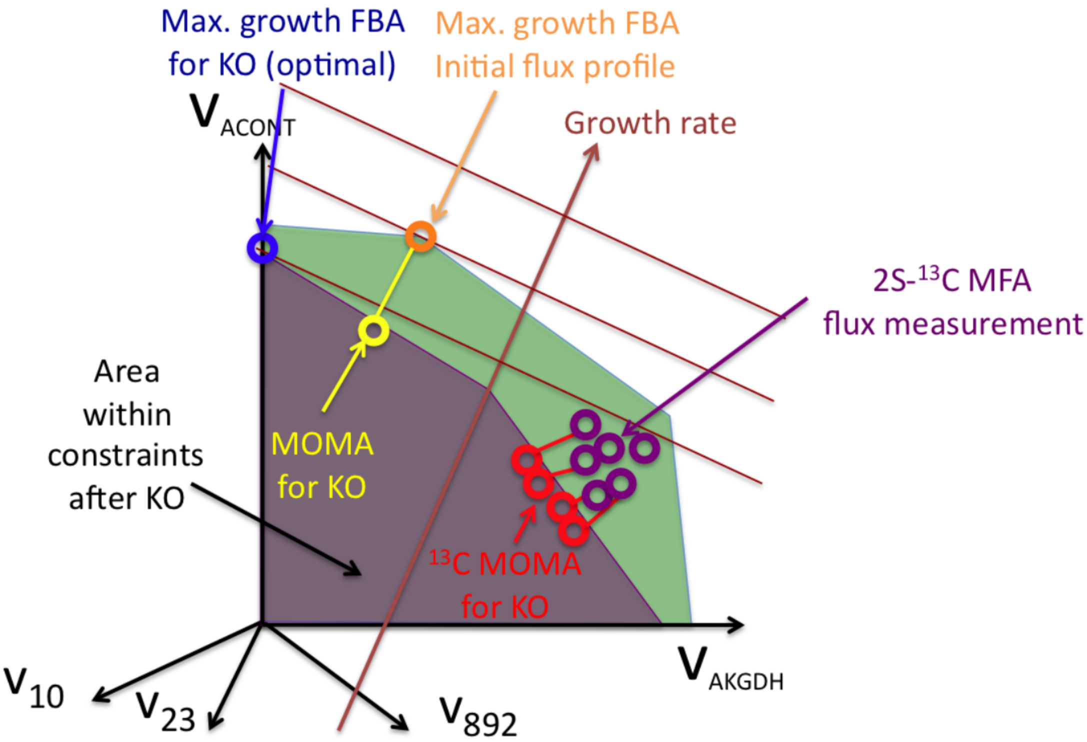

Figure 1: Comparison between standard MOMA and ^{13}C-MOMA in flux phase space[1]. The orange circle represents the FBA maximum growth result for the wild type and the original flux profile (w_j^r) for the MOMA. The magenta area represents the allowable phase space after the gene KO. The blue circle represents the FBA maximum growth prediction for the gene KO. The yellow circle is the MOMA prediction, which is the flux profile closest to the original FBA flux profile. The violet circles represent the flux measurements through 2S-^{13}C MFA. Multiple circles are presented since the ^{13}C data does not completely constrains fluxes. The red circles at the bottom represent the ^{13}C-MOMA predictions for each of the measurements.

MOMA

$$\text{Minimize} \quad \sum_j (v_j - w_j)^2 \qquad j \in J \tag{1}$$

Subject to:

$$\sum_j S_{ij} v_j = 0 \qquad \forall i \in I^N, j \in J \tag{2}$$

$$lb_j \leq v_j \leq ub_j \qquad \forall j \in J \tag{3}$$

$$v_j = 0 \qquad \forall j \in J_{KO} \tag{4}$$

where:

Sets	
$I^N \subset I$	: Set of non-exchange metabolites.
$J = \{j\}$	: Set of fluxes.
$J_{KO} \subset J$	: Set of fluxes corresponding to reactions being knocked out.
Parameters	
S_{ij}	: Stoichiometry matrix.
ub_j, lb_j	: Upper and lower bounds for reaction j.
w_j	: Initial flux value for reaction j.
Variables	
v_j	: Flux value of reaction j, in mmol/gdw/h.

ROOM

$$\text{Minimize} \quad \sum_j y_j \qquad \forall j \in J \tag{5}$$

Subject to:

$$\sum_j S_{ij} v_j = 0 \qquad \forall i \in I^N, j \in J \tag{6}$$

$$v_j = 0 \qquad \forall j \in J_{KO} \tag{7}$$

$$v_j \leq w_j^u + y_j(ub_j - w_j^u) \qquad \forall j \in J \tag{8}$$

$$v_j \geq w_j^u + y_j(lb_j - w_j^u) \qquad \forall j \in J \tag{9}$$

$$w_j^u = w_j + \delta|w_j| + \epsilon \qquad \forall j \in J \tag{10}$$

$$w_j^u = w_j - \delta|w_j| - \epsilon \qquad \forall j \in J \tag{11}$$

$$y_j \in \{0, 1\} \qquad \forall j \in J \tag{12}$$

where:

Sets	
$I^N \subset I$	: Set of non-exchange metabolites.
$J = \{j\}$	: Set of fluxes.
$J_{KO} \subset J$	: Set of fluxes corresponding to reactions being knocked out.
Parameters	
S_{ij}	: Stoichiometry matrix.
ub_j, lb_j	: Upper and lower bounds for reaction j.
w_j	: Initial flux value for reaction j.
δ	= 0.03. Relative range of tolerance, see Shlomi *et al* [2].
ϵ	= 0.002. Relative range of tolerance, see Shlomi *et al* [2].
Variables	
v_j	: Flux value of reaction j, in mmol/gdw/h.

^{13}C MOMA and ^{13}C ROOM

These methods use the same optimization problem as MOMA and ROOM (see above), but the initial profile w_j is obtained from 2S-^{13}C MFA instead of FBA. Exchange fluxes were left unchanged from the 2S-^{13}C MFA profile in the prediction. In order to obtain confidence intervals for the predictions, several initial profiles w_j^r were used, obtained by randomizing the target labeling data (MDVs). For each randomization $r = 1..R$, a new set of MDVs for each metabolite were created by setting them to a random value bounded by the experimental error:

$$f_{em}^{exp,r} = f_{em}^{exp} + \Delta_{em}(2\xi - 1) \tag{13}$$

where $\xi \in [0, 1]$ is a random number.

These randomized labeling values were used in 2S-^{13}C MFA to obtain a w_j^r and that profile was used through MOMA/ROOM to obtain a prediction v_j^r. The standard deviation of v_j^r was used as confidence interval for the ^{13}C MOMA/ROOM predictions. We used $R = 10$.

All problems were solved through GAMS, using the CONOPT solver for MOMA and CPLEX for ROOM.

References

[1] Daniel Segrè, Dennis Vitkup, and George M Church. Analysis of optimality in natural and perturbed metabolic networks. *Proceedings of the National Academy of Sciences of the United States of America*, 99(23):15112–7, November 2002.

[2] Tomer Shlomi, Omer Berkman, and Eytan Ruppin. Regulatory on/off minimization of metabolic flux changes after genetic perturbations. *Proceedings of the National Academy of Sciences of the United States of America*, 102(21):7695–700, May 2005.
