# Supplementary material for: A Method to Constrain Genome-Scale Models with 13C Labeling Data
Source: PLoS Comput Biol. 2015 Sep 17;11(9):e1004363. doi: 10.1371/journal.pcbi.1004363 (PMC4574858; doi:10.1371/journal.pcbi.1004363)
Supplement: S3 Text — (PDF) [file pcbi.1004363.s003.pdf]

Text S3: Appendix C

"A method to constrain genome-scale models with ^{13}C labeling data"

Héctor García Martín[1,2,*], Vinay Satish Kumar[1,2], Daniel Weaver[1,2],Amit Ghosh[1,2], Victor Chubukov[1,2], Aindrila Mukhopadhyay[1,2], Adam Arkin[1,3], Jay D. Keasling[1,2,3,4]

June 17, 2015

1 Reactions and carbon transitions

1.1 Core reactions for 2S-^{13}C MFA

The following list comprises the reactions included in the core set, along with their corresponding carbon transitions. Carbon transitions indicate the fate of each carbon in the reaction. For example, for reaction PDH below, the first carbon in *pyr* (a) becomes co_2 and the remaining two make up acetyl-CoA (*accoa*) Notice that the transketolase and transaldolase transitions have been substituted by their more complete version, as reported by Kleijn *et al*[3]. Carbon transitions have been obtained from Antoniewic *et al*[1] and EcoCyc[2]. Original core reactions are in black and the reactions added after the ELVA are shown in blue:

Input reactions

Reaction Name	*Reactants*	*Reversible?*	*Products*	*Carbon transitions*
GLCt2	glc-D[e]	−− >	glc-D	abcdef : abcdef
GLCpts	glc-D[e] + pep	−− >	g6p + pyr	abcdef + ABC : abcdef + ABC
EX_glc(e)	glc-D[e]	<==>		

Glycolysis/gluconeogenesis

HEX1	glc-D	−− >	g6p	abcdef : abcdef
PGI	g6p	<==>	f6p	abcdef : abcdef
PFK	f6p	−− >	fdp	abcdef : abcdef
F6PA	f6p	−− >	g3p + dha	CBAabc : abc + ABC
FBA	fdp	<==>	g3p + dhap	CBAabc : abc + ABC
TPI	dhap	<==>	g3p	abc : abc
GAPD	g3p	−− >	13dpg	abc : abc
PGK	3pg	<==>	13dpg	abc : abc
PGM	2pg	<==>	3pg	abc : abc
PYK	pep	−− >	pyr	abc : abc
PDH	pyr	−− >	co2 + accoa	abc : a + bc

Penthose Phosphate Pathway

G6PDH2r	g6p	−− >	6pgl	abcdef : abcdef
PGL	6pgl	−− >	6pgc	abcdef : abcdef
GND	6pgc	−− >	co2 + ru5p-D	abcdef : a + bcdef
RPE	ru5p-D	<==>	xu5pD	abcde : abcde
RPI	r5p	<==>	ru5p-D	abcde : abcde
TK1	xu5pD	<==>	TKC2 + g3p	abcde : ab + cde
TK2	f6p	<==>	TKC2 + e4p	abcdef : ab + cdef
TK3	s7p	<==>	TKC2 + r5p	abcdefg : ab + cdefg
TA1	f6p	<==>	TAC3 + g3p	abcdef : abc + def
TA2	s7p	<==>	TAC3 + e4p	abcdefg : abc + defg
EDD	6pgc	−− >	2ddg6p	abcdef : abcdef
EDA	2ddg6p	−− >	pyr + g3p	abcdef : abc + def

Citric Acid Cycle/TCA

CS	oaa + accoa	−− >	cit	abcd + AB : dcbBAa
ACONT	cit	−− >	icit	abcdef : abcdef
ICDHyr	icit	−− >	akg + co2	abcdef : abcde + f
AKGDH	akg	−− >	succoa + co2	abcde : bcde + a
SUCOAS	succ	<==>	succoa	bcde : (bcde;edcb)
SUCD1i	succ	−− >	fum	abcd : (abcd;dcba)
FUM	fum	−− >	mal-L	abcd : (abcd;dcba)
MDH	mal-L	<==>	oaa	abcd : abcd

Anaplerotic reactions

DHAPT	dha + pep	−− >	dhap + pyr	ABC + abc : ABC + abc
ENO	2pg	<==>	pep	abc : abc
PPC	pep + co2	−− >	oaa	abc + d : abcd
PPCK	oaa	−− >	pep + co2	abcd : abc + d
ME1	mal-L	−− >	pyr + co2	abcd : abc + d
ME2	mal-L	−− >	pyr + co2	abcd : abc + d
ICL	icit	−− >	succ + glx	ABCDEF : FCDE + AB
MALS	glx + accoa	−− >	mal-L	AB + CD : ABCD

Alanine and aspartate metabolism

ALATA_L	ala-L + akg	<==>	pyr + glu-L	abc + ABCDE : abc + ABCDE
ASPTA	akg + asp-L	<==>	glu-L + oaa	ABCDE + abcd : ABCDE + abcd

Glutamate metabolism

GLUDy	glu-L	<==>	akg	abcde : abcde
GLNS	glu-L	−− >	gln-L	abcde : abcde

Glycine and serine metabolism

PGCD	3pg	−− >	3php	abc : abc
PSERT	glu-L + 3php	−− >	akg + pser-L	ABCDE + abc : ABCDE + abc
PSP_L	pser-L	−− >	ser-L	abc : abc
GHMT2	ser-L	−− >	mlthf + gly	abc : c + ab
THRD_L	thrL	−− >	2obut	ABCD : ABCD

Threonine and lysine metabolism

ASPK	asp-L	<==>	4pasp	abcd : abcd
ASAD	aspsa	<==>	4pasp	abcd : abcd
HSDy	homL	<==>	aspsa	abcd : abcd
HSK	homL	−− >	phom	abcd : abcd
THRS	phom	−− >	thrL	abcd : abcd
THRAr	thrL	<==>	acald + gly	abcd : cd + ab
SDPTA	akg + sl26da	<==>	glu-L + sl2a6o	ABCDE + abcdefghijk : ABCDE + abcdefghijk

Valine, leucine and isoleucine metabolism

ACLS	pyr + pyr	−− >	alac-S + co2	cde + fgh : fgdhe + c
KARA1i	alac-S	−− >	23dhmb	abcde : abcde
DHAD1	23dhmb	−− >	3mob	abcde : abcde
VALTA	akg + val-L	<==>	glu-L + 3mob	ABCDE + abcde : ABCDE + abcde
ACHBS	2obut + pyr	−− >	2ahbut + co2	abcd + ABC : abBcCd + A
KARA2i	2ahbut	−− >	23dhmp	abcdef : abcdef
DHAD2	23dhmp	−− >	3mop	abcdef : abcdef
ILETA	akg + ile-L	<==>	glu-L + 3mop	ABCDE + abcdef : ABCDE + abcdef
LEUTAi	4mop + glu-L	−− >	akg + leu-L	abcdef + ABCDE : ABCDE + abcdef

Tyrosine, tryptophan and phenylalanine metabolism

DDPA	e4p + pep	−− >	2dda7p	ABCD + abc : abcABCD
DHQS	2dda7p	−− >	3dhq	abcdefg : abcdefg
DHQD	3dhq	<==>	3dhsk	abcdefg : abcdefg
SHK3Dr	3dhsk	<==>	skm	abcdefg : abcdefg
SHKK	skm	−− >	skm5p	abcdefg : abcdefg
PSCVT	skm5p + pep	−− >	3psme	abcABCD + def : abcefABCDd
CHORS	3psme	−− >	chor	abcdefghij : abcdefghij
CHORM	chor	−− >	pphn	abcdefghij : abcdefghij
PPNDH	pphn	−− >	phpyr + co2	abcdefghij : abcdefghi + j
PHETA1	akg + phe-L	<==>	glu-L + phpyr	ABCDE + abcdefghi : ABCDE + abcdefghi
PPND	pphn	−− >	34hpp + co2	abcdefghij : abcdefghi + j
TYRTA	akg + tyr-L	<==>	glu-L + 34hpp	ABCDE + abcdefghi : ABCDE + abcdefghi

Arginine and proline metabolism

GLU5K	glu-L	-- >	glu5p	abcde : abcde
G5SD	glu5p	-- >	glu5sa	abcde : abcde
G5SADs	glu5sa	-- >	1pyr5c	abcde : abcde
P5CR	1pyr5c	-- >	pro-L	abcde : abcde
ACGS	accoa + glu-L	-- >	acglu	pq + abcde : abcdepq
ACGK	acglu	-- >	acg5p	abcdepq : abcdepq
AGPR	acg5sa	<==>	acg5p	abcdepq : abcdepq
ACOTA	acorn + akg	<==>	acg5sa + glu-L	abcdepq + ghijk : abcdepq + ghijk
ACODA	acorn	-- >	ac + orn	abcdepq : pq + abcde
CBPS	gln-L + hco3	-- >	cbp + glu-L	ghijk + f : f + ghijk
OCBT	cbp + orn	<==>	citr-L	f + abcde : abcdef
ARGSS	asp-L + citr-L	-- >	argsuc	lmno + abcdef : abcdeflmno
ARGSL	argsuc	<==>	arg-L + fum	abcdeflmno : abcdef + (lmno;onml)
ARGDC	arg-L	-- >	agm + co2	abcdef : bcdef + a
AGMT	agm	-- >	ptrc + urea	bcdef : (bcde;edcb) + f
PTRCTA	ptrc + akg	-- >	4abutn + glu-L	(bcde;edcb) + ABCDE : bcde + ABCDE
ABUTD	4abutn	-- >	4abut	bcde : bcde
ABTA	4abut + akg	-- >	sucsal + glu-L	bcde + ABCDE : bcde + ABCDE
SSALx	sucsal	-- >	succ	bcde : (bcde;edcb)
SSALy	sucsal	-- >	succ	bcde : (bcde;edcb)

Purine and pyrimidine metabolism

GLUPRT	gln-L + prpp	-- >	glu-L + pram	abcde + ABCDE : abcde + ABCDE
GMPS2	gln-L	-- >	glu-L	abcde : abcde
ADSS	asp-L	-- >	dcamp	abcd : abcd (dcamp has been simplified)
ADSL1r	dcamp	-- >	fum	abcd : (abcd;dcba)
PRAGSr	gly + pram	<==>	gar	ab + ABCDE : ABCDEab
GART	for + gar	-- >	fgam	a + ABCDEFG : ABCDEFGa
PRFGS	fgam + gln-L	-- >	fpram + glu-L	ABCDEFGH + abcde : ABCDEFGH + abcde
PRAIS	fpram	-- >	air	ABCDEFGH : ABCDEFGH
AIRC2	air + hco3	-- >	5caiz	ABCDEFGH + a : ABCDEFGHa
AIRC3	5aizc	<==>	5caiz	ABCDEFGHI : ABCDEFGHI
PRASCS	5aizc + asp-L	<==>	25aics	ABCDEFGHI + abcd : ABCDEFGHIcdba
ADSL2r	25aics	-- >	aicar + fum	ABCDEFGHIcdba : ABCDEFGHI + (abcd;bcda)

Output reactions

L_LACD2	lac_L_c	<==>	pyr	abc : abc
L_LACD3	lac_L_c	<==>	pyr	abc : abc
L_LACt2r	lac_L[e]	<==>	lac-L	abc : abc
ETOHt2r	etoh[e]	<==>	etoh	ab : ab
POX	pyr	-- >	ac + co2	abc : bc + a
EX_lac_L(e)	lac-L[e]	<==>		
EX_etoh(e)	etoh[e]	<==>		

Other reactions

GLYCL	gly	<==>	co2 + mlthf	ab : a + b
ACALDi	acald	-- >	accoa	ab : ab
HCO3E	co2	<==>	hco3	f : f
CBMK	co2	-- >	cbp	a : a
PRPPS	r5p	<==>	prpp	abcde : abcde
ADHEr	accoa	-- >	etoh	ab : ab
ACACCT	accoa	-- >	ac[e]	ab : ab
PTAr	accoa	-- >	actp	ab : ab
ACKr	ac	<==>	actp	ab : ab

1.2 Reactions for ^{13}C MFA

The values of the output reactions and reactions to biomass have been inferred from the flux maps in [4].

Input reactions

Reaction Name	*Reactants*	*Reversible?*	*Products*	*Carbon transitions*
GLCpts	glc-D[e] + pep	-- >	g6p + pyr	abcdef + ABC : abcdef + ABC

Glycolysis/gluconeogenesis

PGI	g6p	<==>	f6p	abcdef : abcdef
PFK	f6p	-- >	fdp	abcdef : abcdef
FBA	fdp	<==>	g3p + dhap	CBAabc : abc + ABC
TPI	dhap	<==>	g3p	abc : abc
GAPD	g3p	-- >	13dpg	abc : abc
PGK	3pg	<==>	13dpg	abc : abc
PGM	2pg	<==>	3pg	abc : abc
PYK	pep	-- >	pyr	abc : abc
PDH	pyr	-- >	co2 + accoa	abc : a + bc

Penthose Phosphate Pathway

G6PDH2r	g6p	-- >	6pgl	abcdef : abcdef
PGL	6pgl	-- >	6pgc	abcdef : abcdef
GND	6pgc	-- >	co2 + ru5p-D	abcdef : a + bcdef
RPE	ru5p-D	<==>	xu5pD	abcde : abcde
RPI	r5p	<==>	ru5p-D	abcde : abcde
TK1	xu5pD	<==>	TKC2 + g3p	abcde : ab + cde
TK2	f6p	<==>	TKC2 + e4p	abcdef : ab + cdef
TK3	s7p	<==>	TKC2 + r5p	abcdefg : ab + cdefg
TA1	f6p	<==>	TAC3 + g3p	abcdef : abc + def
TA2	s7p	<==>	TAC3 + e4p	abcdefg : abc + defg
EDD	6pgc	-- >	2ddg6p	abcdef : abcdef
EDA	2ddg6p	-- >	pyr + g3p	abcdef : abc + def

Citric Acid Cycle/TCA

CS	oaa + accoa	-- >	cit	abcd + AB : dcbBAa
ACONT	cit	-- >	icit	abcdef : abcdef
ICDHyr	icit	-- >	akg + co2	abcdef : abcde + f
AKGDH	akg	-- >	succoa + co2	abcde : bcde + a
SUCOAS	succ	<==>	succoa	bcde : (bcde;edcb)
SUCD1i	succ	-- >	fum	abcd : (abcd;dcba)
FUM	fum	-- >	mal-L	abcd : (abcd;dcba)
MDH	mal-L	<==>	oaa	abcd : abcd

Anaplerotic reactions

ENO	2pg	<==>	pep	abc : abc
PPC	pep + co2	-- >	oaa	abc + d : abcd
PPCK	oaa	-- >	pep + co2	abcd : abc + d
ME1	mal-L	-- >	pyr + co2	abcd : abc + d
ME2	mal-L	-- >	pyr + co2	abcd : abc + d
ICL	icit	-- >	succ + glx	ABCDEF : FCDE + AB
MALS	glx + accoa	-- >	mal-L	AB + CD : ABCD

Glutamate metabolism

GLUDy	glu-L	<==>	akg	abcde : abcde

Glycine and serine metabolism

PGCD	3pg	-- >	3php	abc : abc

Tyrosine, tryptophan and phenylalanine metabolism

DDPA	e4p + pep	-- >	2dda7p	ABCD + abc : abcABCD

Output and reactions to biomass

ACACCT	accoa	-- >	ac[e]	ab : ab
G6Pbm	g6p	-- >	g6pbm	abcdef : abcdef
F6Pbm	f6p	-- >	f6pbm	abcdef : abcdef
PEPbm	pep	-- >	pepbm	abc : abc
AcCoabm	accoa	-- >	accoabm	ab : ab
OAAbm	oaa	-- >	oaabm	abcd : abcd
3PHPbm	3php	-- >	3phpbm	abc : abc
GLUbm	glu-L	-- >	glubm	abcde : abcde
R5Pbm	r5p	-- >	r5pbm	abcde : abcde
2DDA7Pbm	2dda7p	-- >	2dda7pbm	abcdefg : abcdefg
CO2bm	co2	-- >	co2bm	a : a

References

[1] Maciek R Antoniewicz, Joanne K Kelleher, and Gregory Stephanopoulos. Elementary metabolite units (EMU): a novel framework for modeling isotopic distributions. *Metabolic engineering*, 9(1):68–86, January 2007.

[2] Ingrid M Keseler, Amanda Mackie, Martin Peralta-Gil, Alberto Santos-Zavaleta, Socorro Gama-Castro, César Bonavides-Martínez, Carol Fulcher, Araceli M Huerta, Anamika Kothari, Markus Krummenacker, Mario Latendresse, Luis Muñiz Rascado, Quang Ong, Suzanne Paley, Imke Schröder, Alexander G Shearer, Pallavi Subhraveti, Mike Travers, Deepika Weerasinghe, Verena Weiss, Julio Collado-Vides, Robert P Gunsalus, Ian Paulsen, and Peter D Karp. EcoCyc: fusing model organism databases with systems biology. *Nucleic acids research*, 41(Database issue):D605–12, January 2013.

[3] Roelco J Kleijn, Wouter A Van Winden, Walter M Van Gulik, and Joseph J Heijnen. Revisiting the 13C-label distribution of the non-oxidative branch of the pentose phosphate pathway based upon kinetic and genetic evidence. *Tracing 13C in the pentose phosphate pathway*, 272(19):4970–4982, 2005.

[4] Yoshihiro Toya, Nobuyoshi Ishii, Kenji Nakahigashi, Takashi Hirasawa, Tomoyoshi Soga, Masaru Tomita, and Kazuyuki Shimizu. 13C-metabolic flux analysis for batch culture of Escherichia coli and its pyk and pgi gene knockout mutants based on mass isotopomer distribution of intracellular metabolites. *Biotechnology progress*, 26(4):975–992, 2010.
