# Supplementary figures and images for: A Method to Constrain Genome-Scale Models with 13C Labeling Data
Source: PLoS Comput Biol. 2015 Sep 17;11(9):e1004363. doi: 10.1371/journal.pcbi.1004363 (PMC4574858; doi:10.1371/journal.pcbi.1004363)

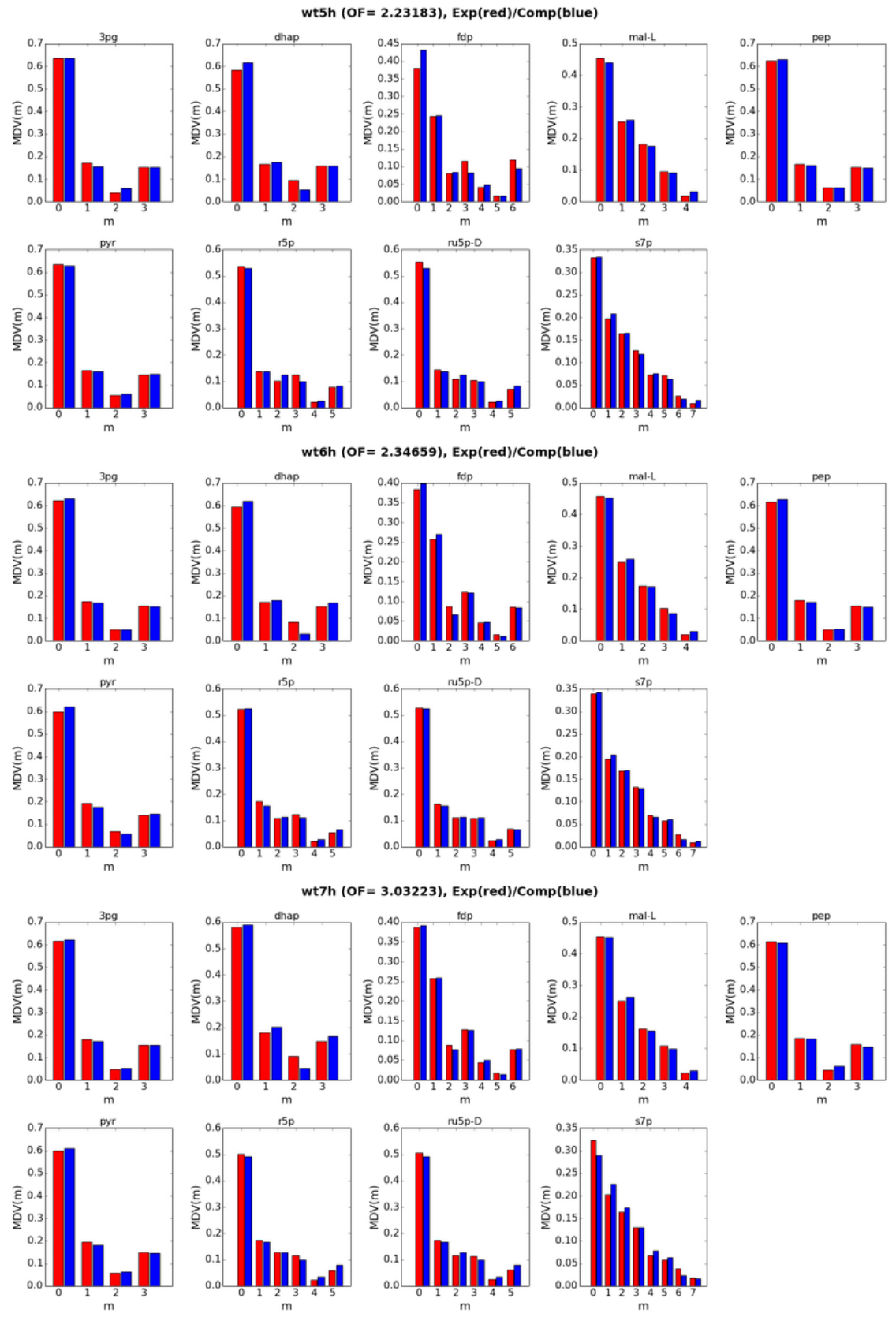

Supplement: S1 Fig — Red denotes the MDV for experimentally measured data, blue columns are the fit. The MDV is the fraction of molecules with m = 1,2,3,4… 13 C incorporated atoms. Sum of square residuals (SSR, [85]) are 216.5, 282.2 and 467.7 for each strain from top to bottom. (TIF) [file pcbi.1004363.s005.tif]

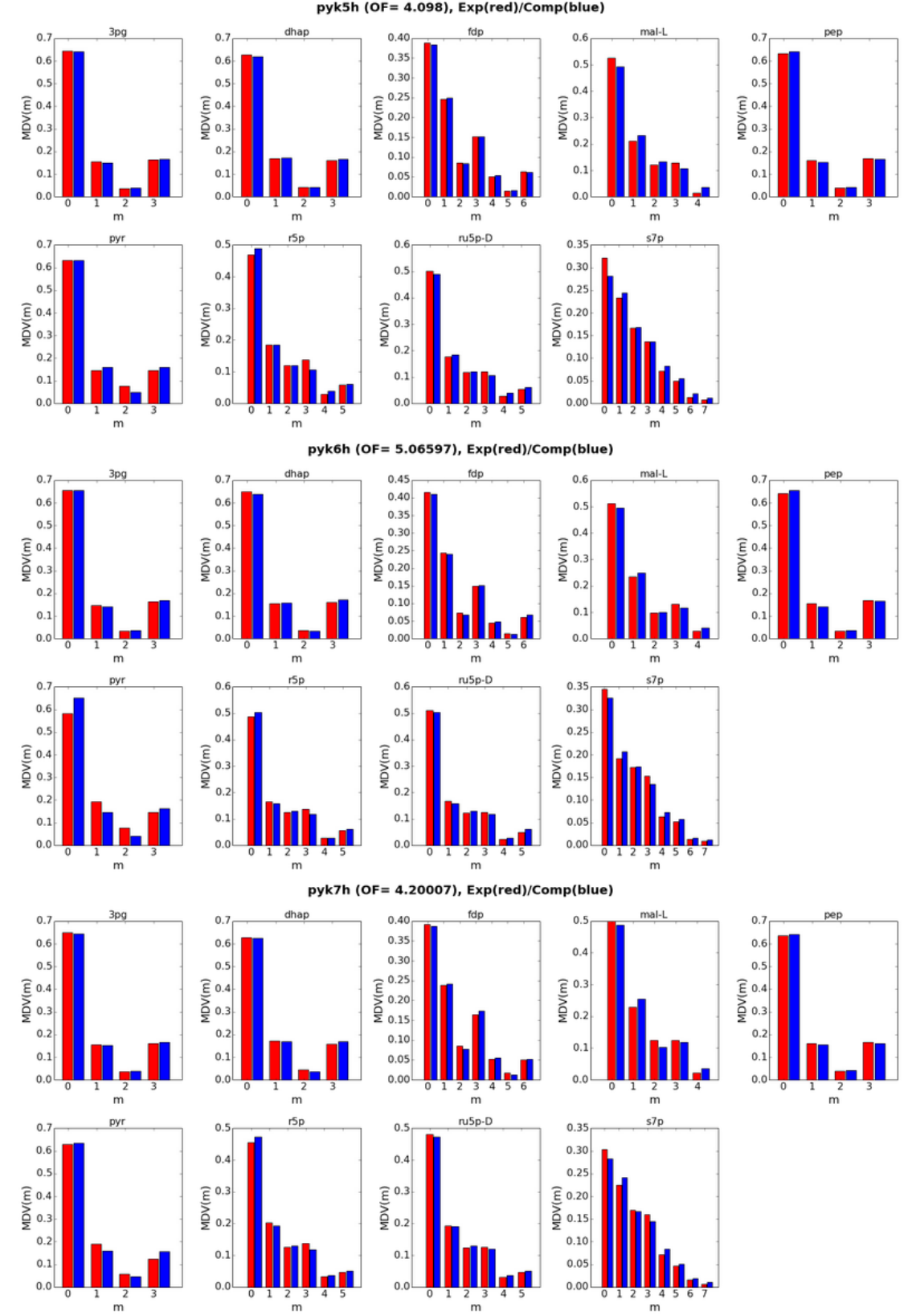

Supplement: S2 Fig — Red denotes the MDV for experimentally measured data, blue columns are the fit. SSRs are 773.2, 1195.9 and 817.36 for each strain from top to bottom. (TIF) [file pcbi.1004363.s006.tif]

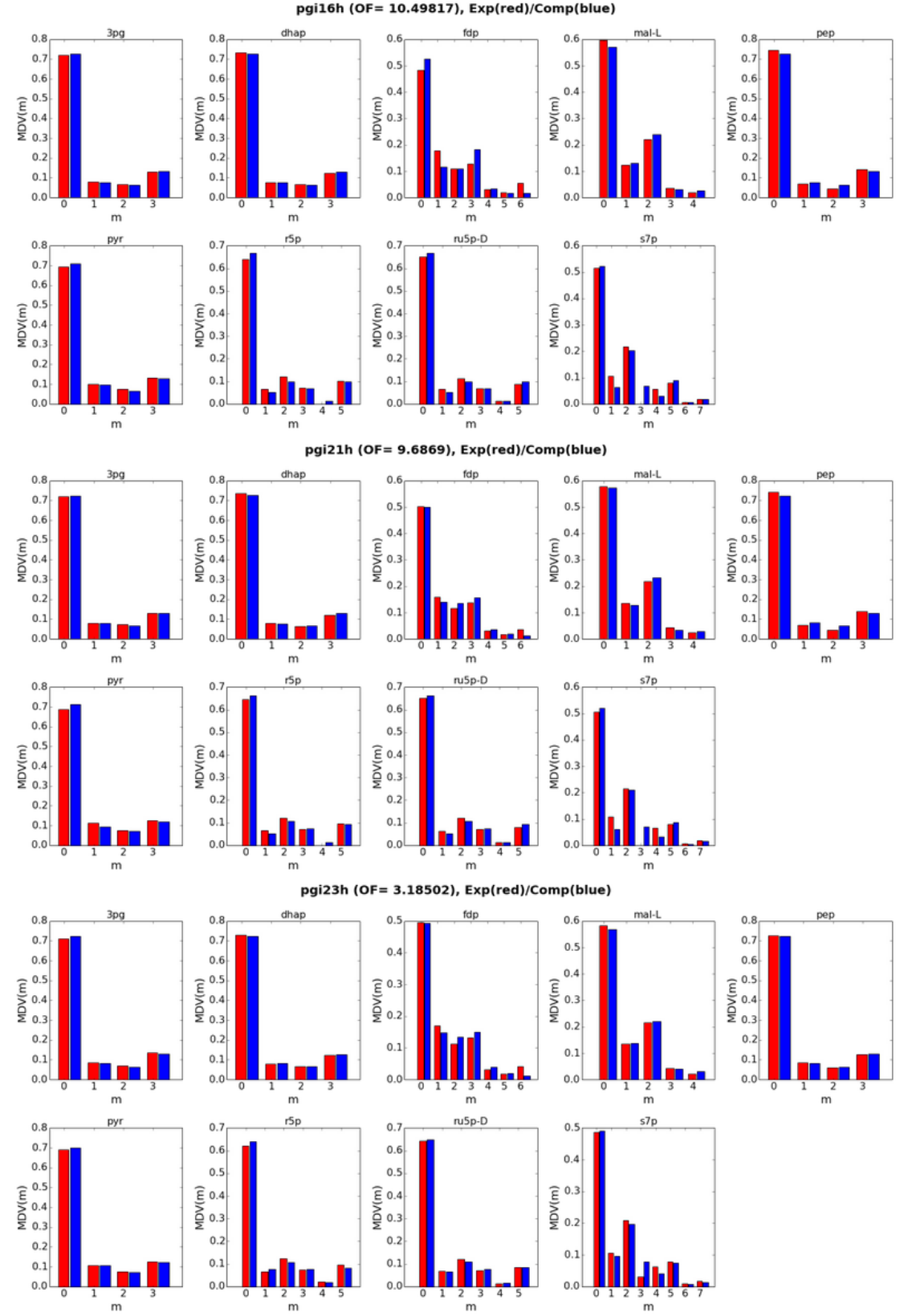

Supplement: S3 Fig — Red denotes the MDV for experimentally measured data, blue columns are the fit. SSRs are 7244.8, 6377.4 and 581.3 for each strain from top to bottom. (TIF) [file pcbi.1004363.s007.tif]

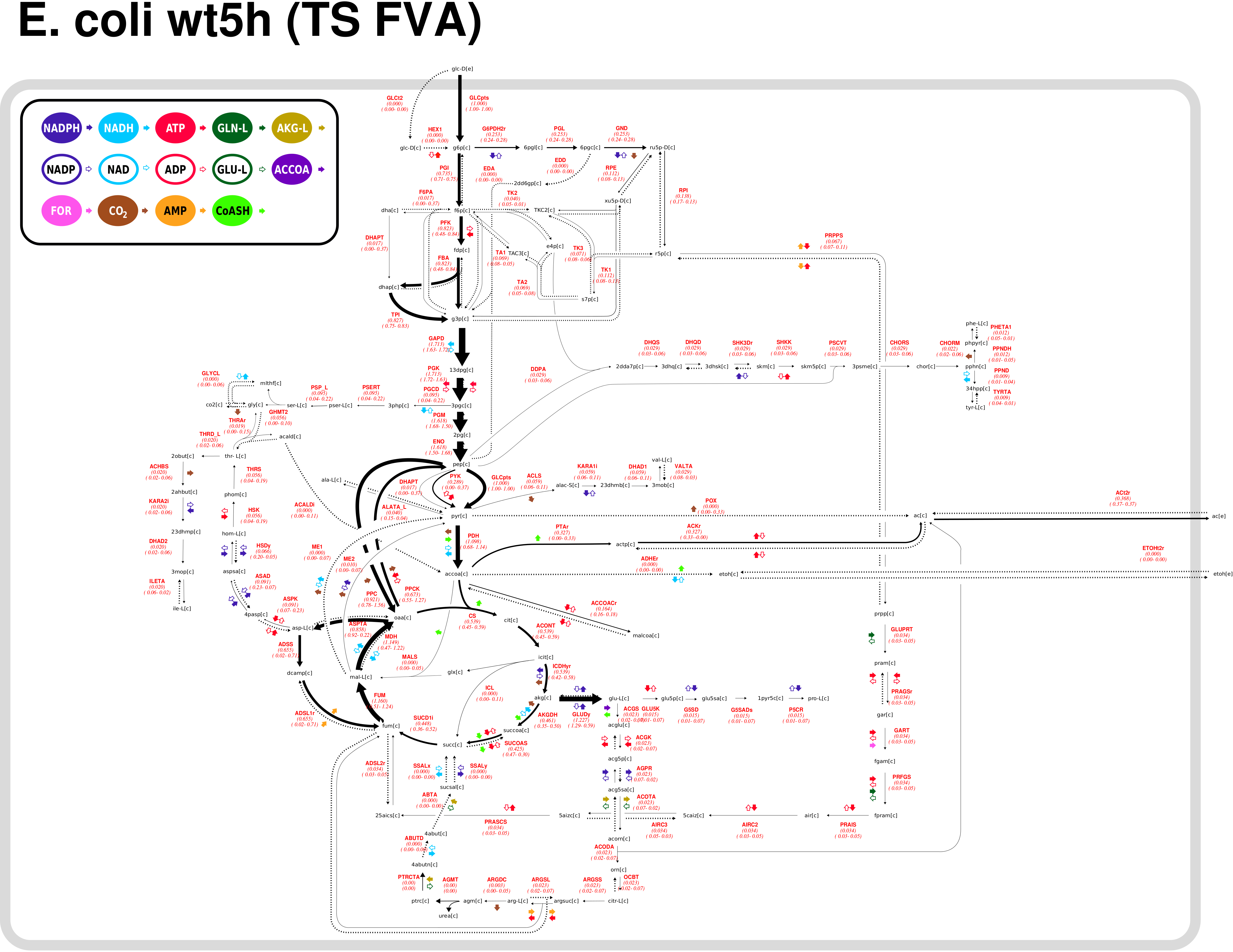

Supplement: S4 Fig — Best fit for flux is given on top red number for each reaction and confidence interval at the bottom. Cofactors and common metabolites are indicated by small arrows. Reversible reactions are indicated by double arrows. (TIF) [file pcbi.1004363.s008.tif]

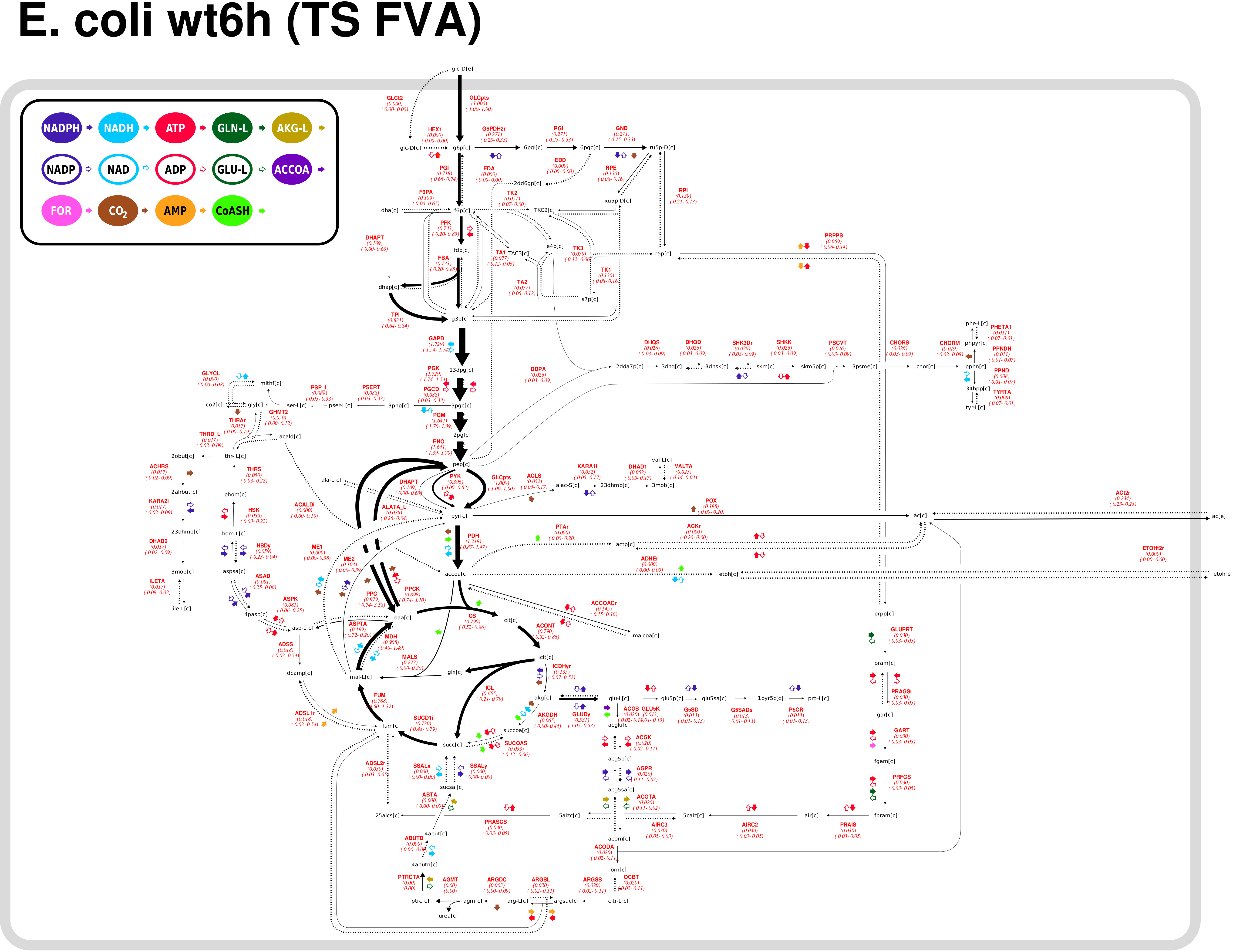

Supplement: S5 Fig — (TIF) [file pcbi.1004363.s009.tif]

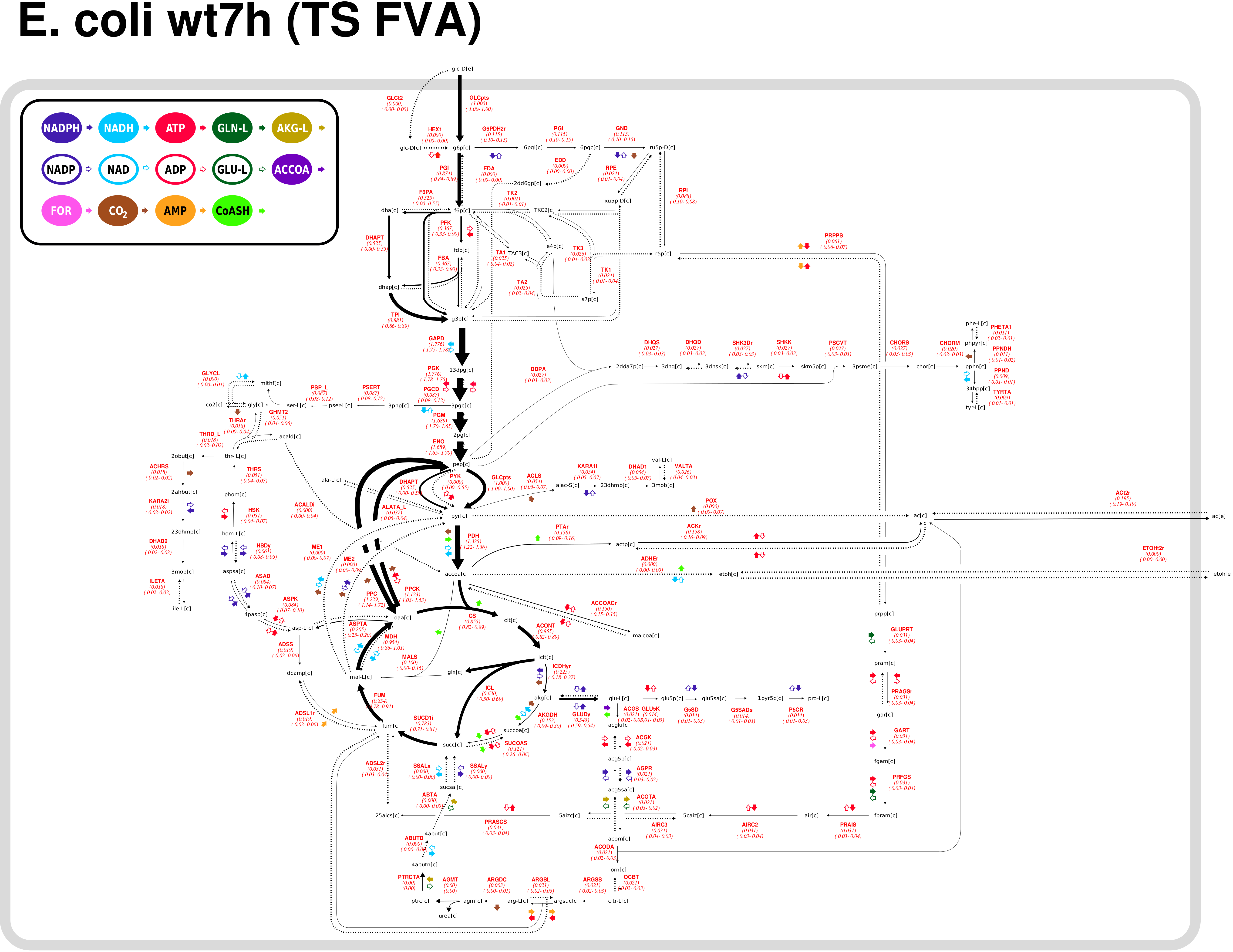

Supplement: S6 Fig — (TIF) [file pcbi.1004363.s010.tif]

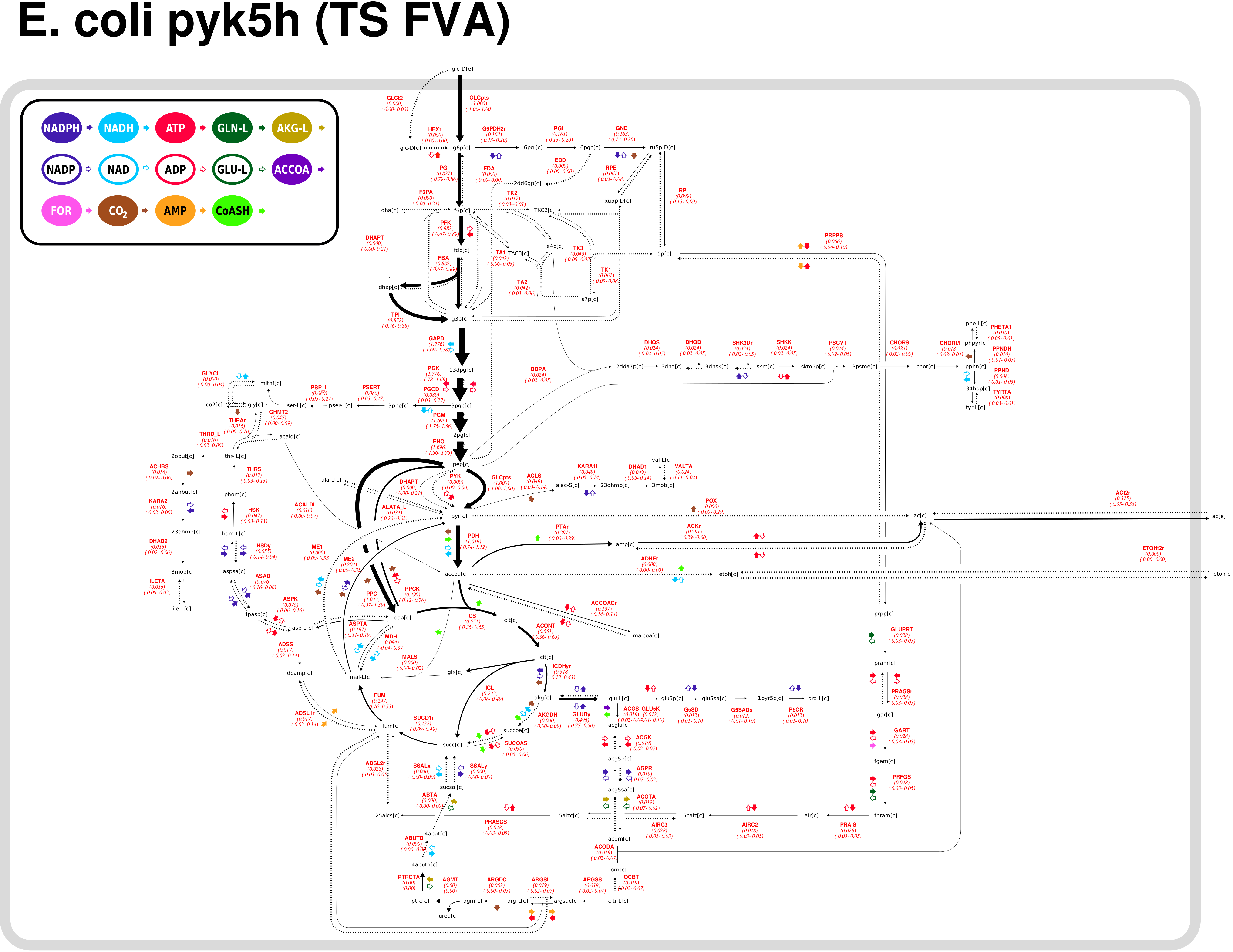

Supplement: S7 Fig — (TIF) [file pcbi.1004363.s011.tif]

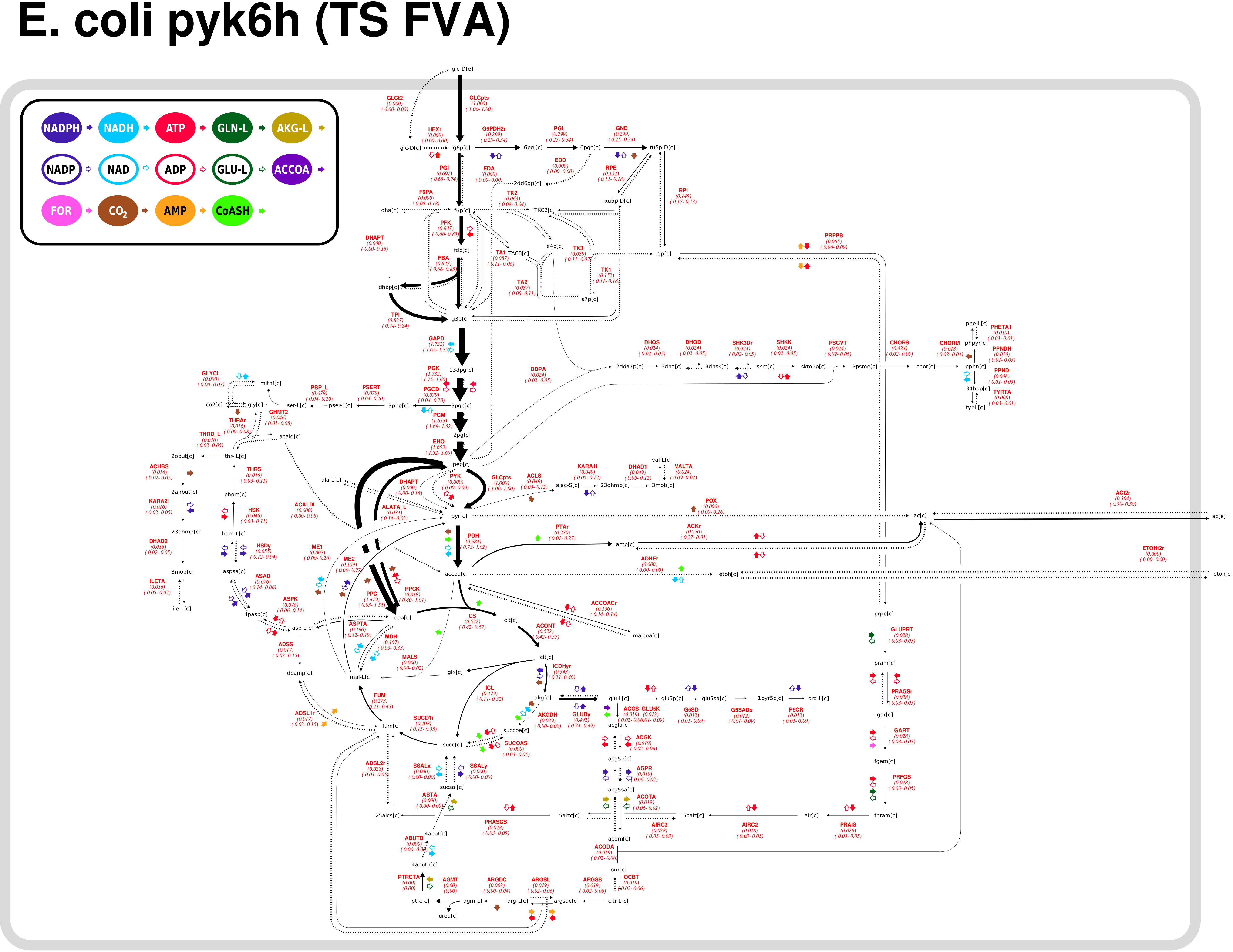

Supplement: S8 Fig — (TIF) [file pcbi.1004363.s012.tif]

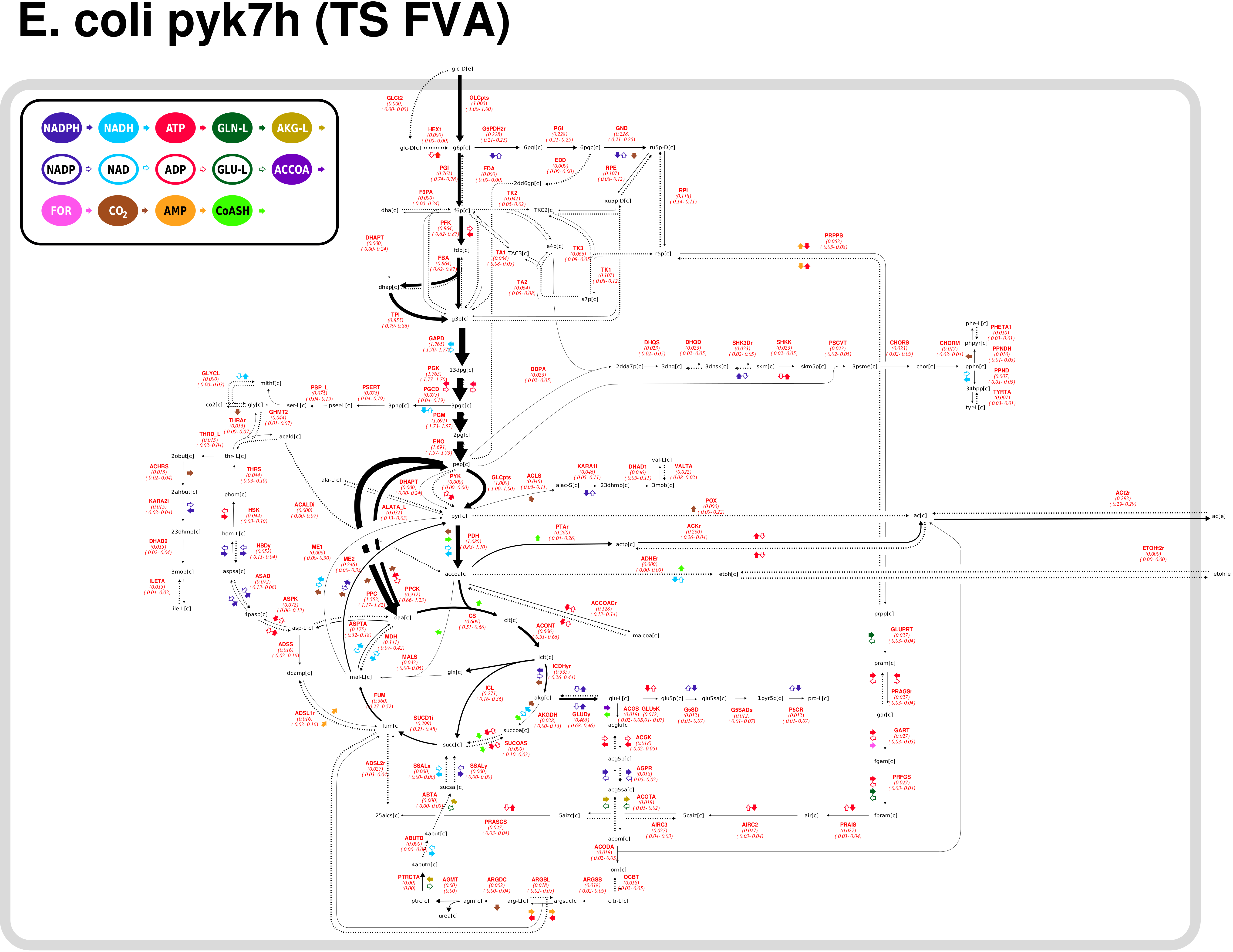

Supplement: S9 Fig — (TIF) [file pcbi.1004363.s013.tif]

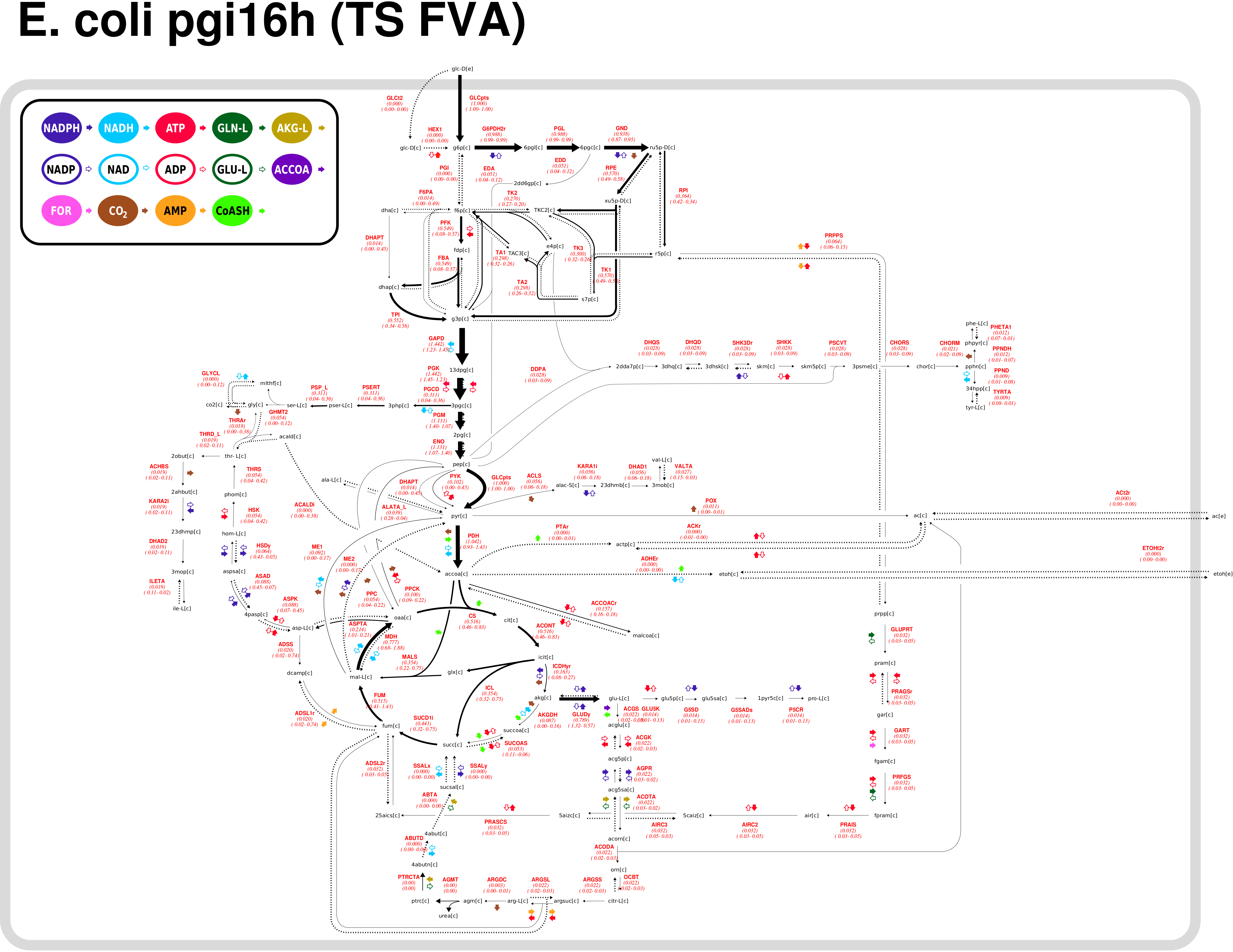

Supplement: S10 Fig — (TIF) [file pcbi.1004363.s014.tif]

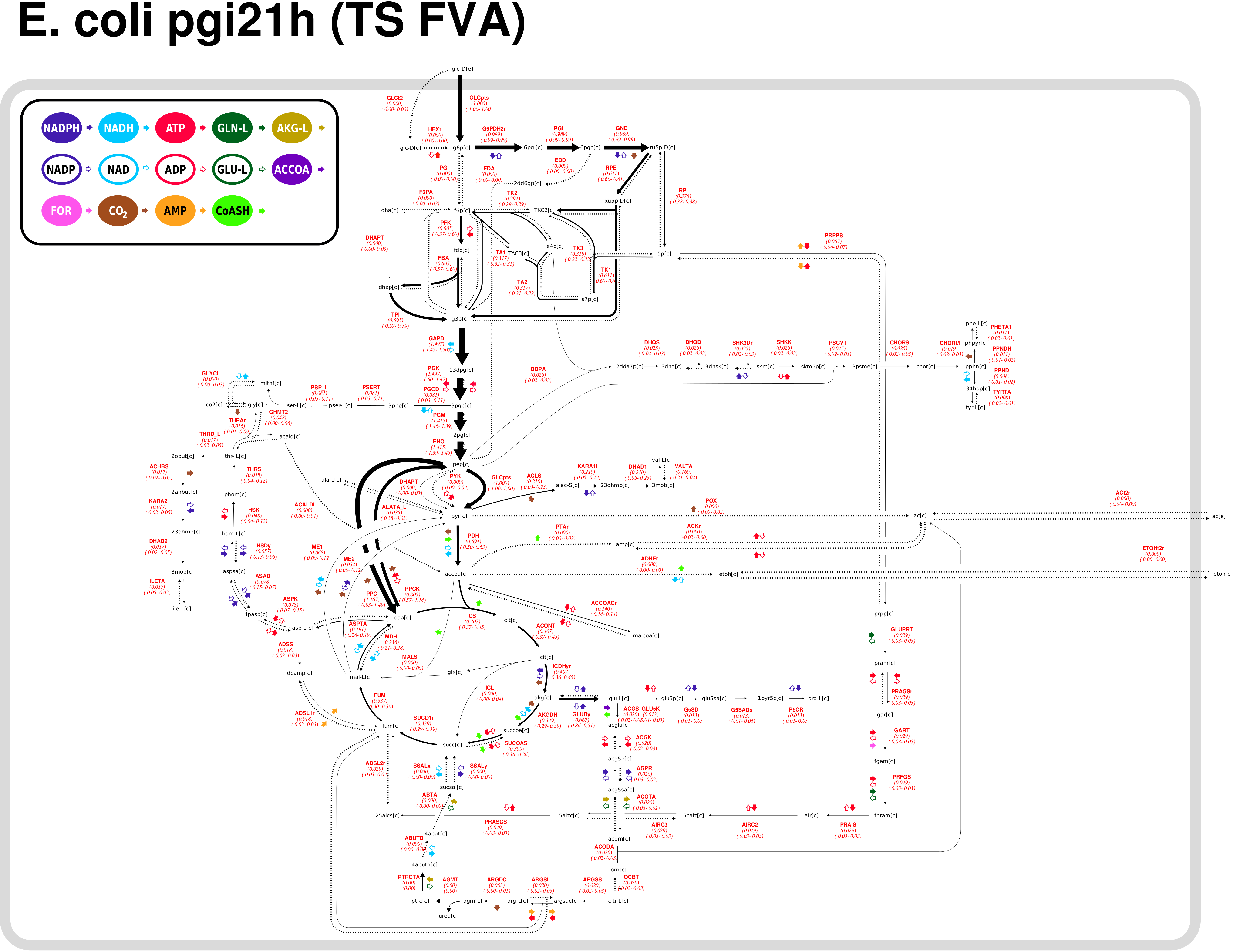

Supplement: S11 Fig — (TIF) [file pcbi.1004363.s015.tif]

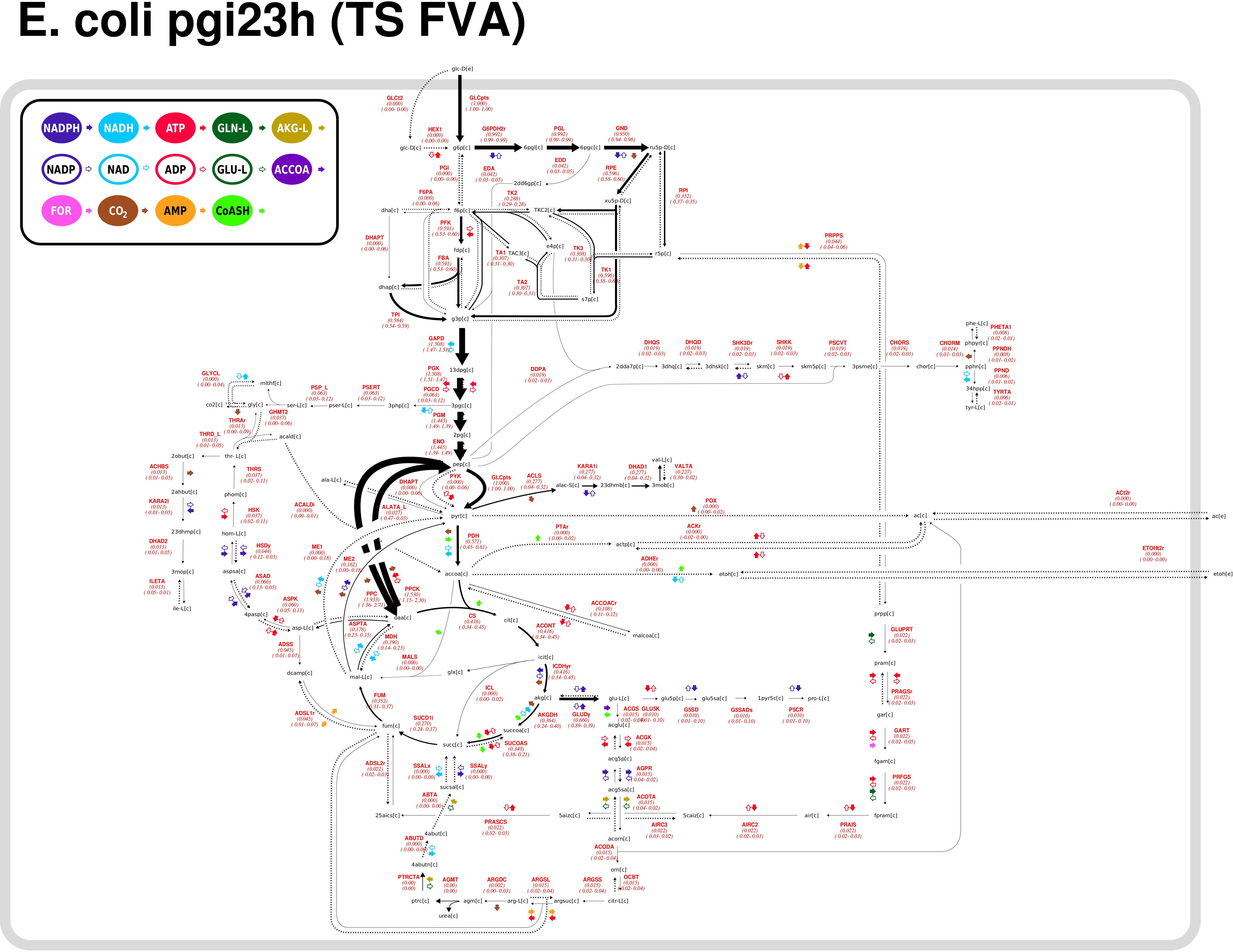

Supplement: S12 Fig — (TIF) [file pcbi.1004363.s016.tif]

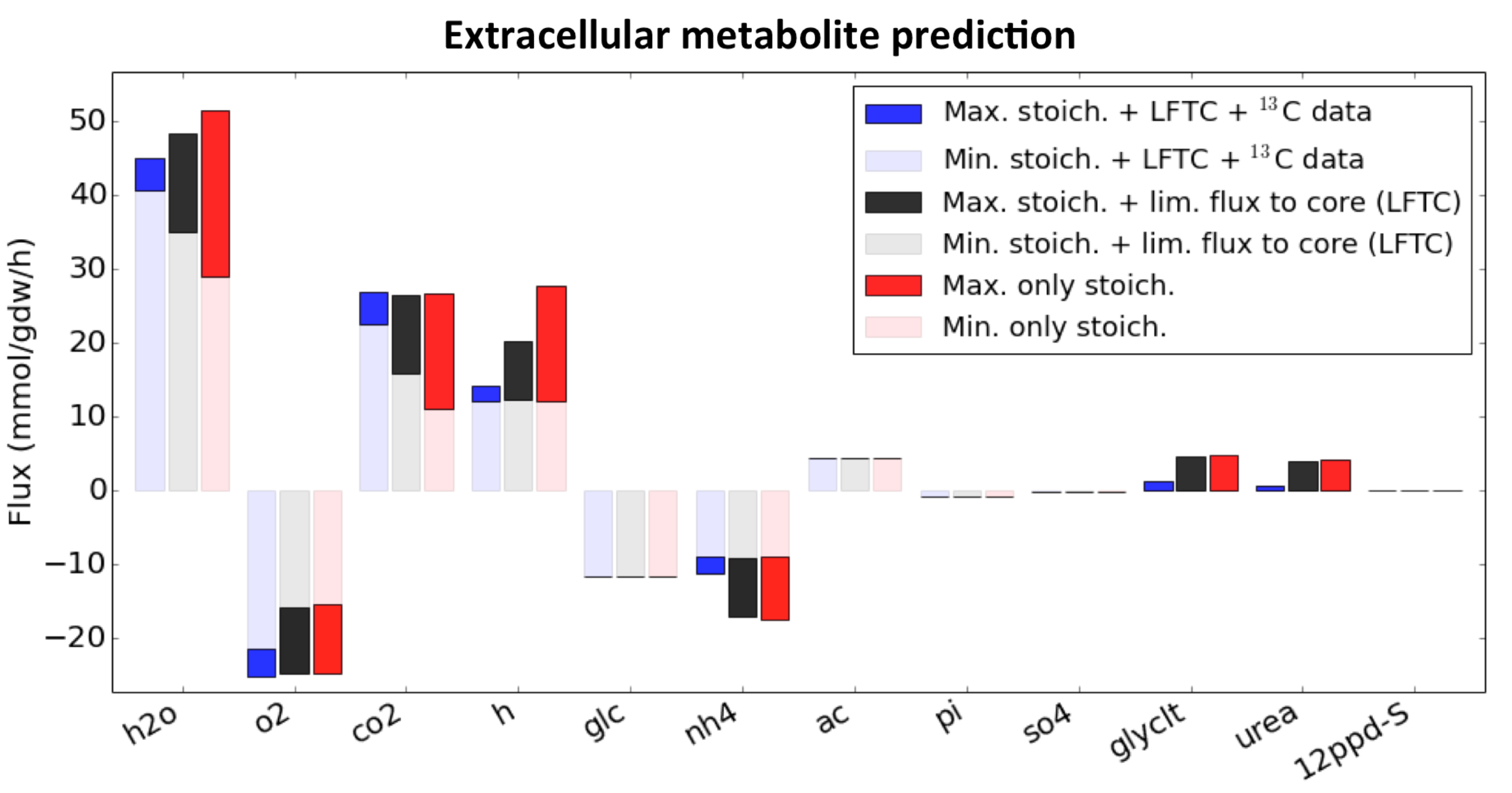

Supplement: S13 Fig — Maximum (dark bar) and minimum (light bar) values of the exchange fluxes obtained by 2S-13C MFA show how 13C experimental data can effectively constrain exchange fluxes that have not been measured (in blue). For comparison, maximum and minimum values for the model constrained by extracellular flux measurements (through FVA, in red) are included as well, as are maximum and minimum values obtained through FVA for a model constrained by extracellular flux measurements along with constraints induced by the two-scale approximation (black, see “Limiting flux to core” section). Fluxes are for the wild type at 5 hrs, and exchanged metabolites are indicated in the x axis (iJR904 notation), [57, 83]). A positive exchange flux (excreted metabolite) that remains positive for long enough should produce a detectable pool of the corresponding metabolite. Acetate and glucose are used as constraints for the flux determination, hence the confidence intervals are very narrow. For this particular case, glycolate and urea are expected in the media. (TIF) [file pcbi.1004363.s017.tif]

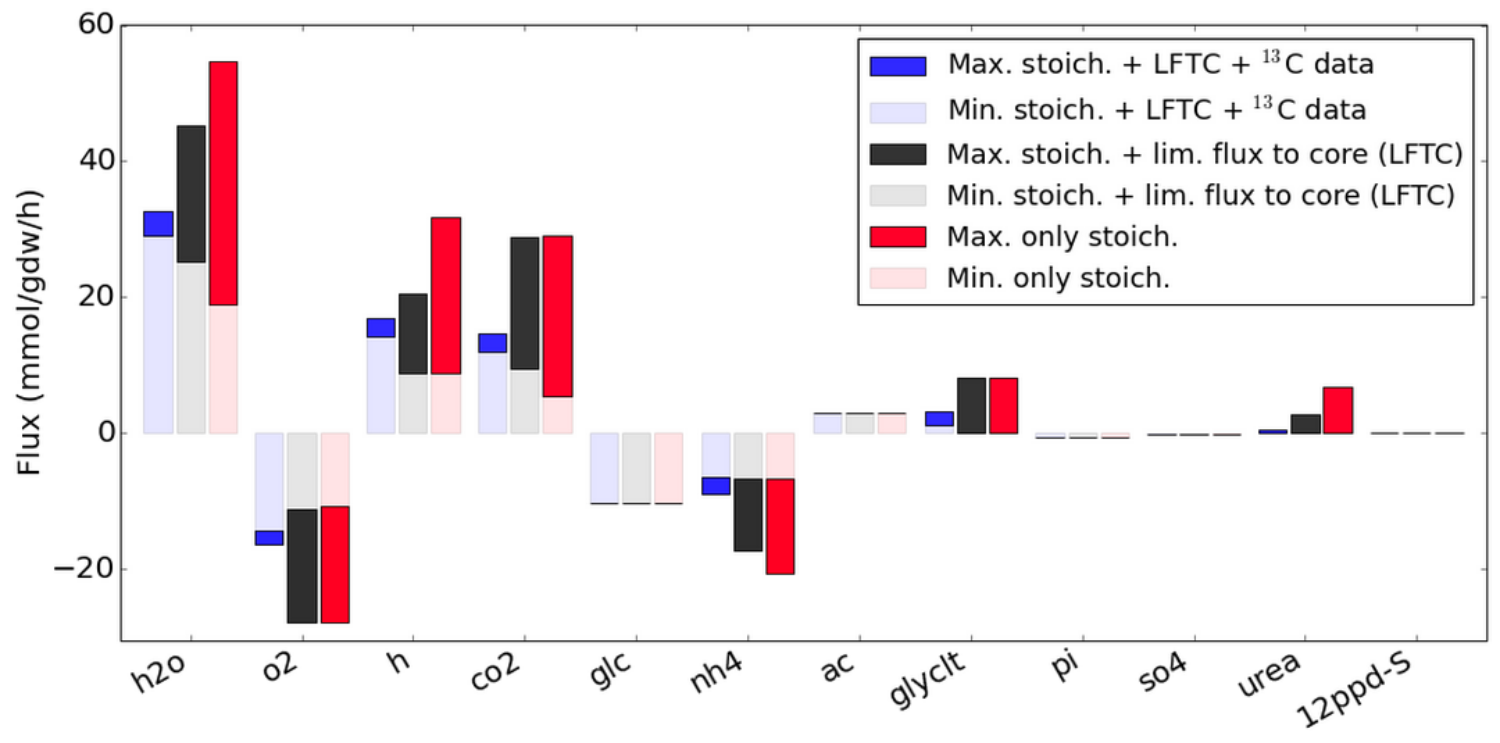

Supplement: S14 Fig — Expected metabolites in the medium include alpha-Ketoglutarate (akg) and glycolate (glyclt). (TIF) [file pcbi.1004363.s018.tif]

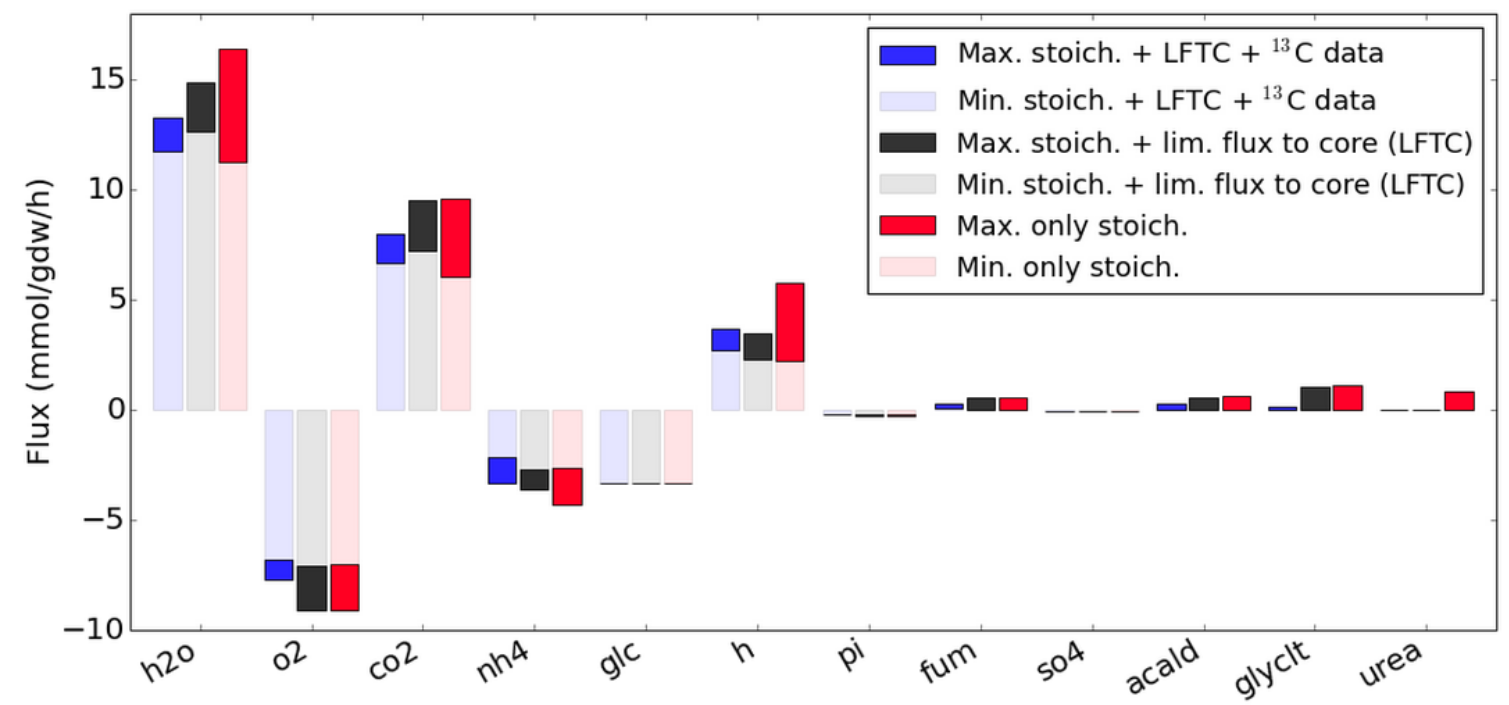

Supplement: S15 Fig — Expected metabolites in the medium include fumarate (fum) and acetaldehyde (acald). (TIF) [file pcbi.1004363.s019.tif]

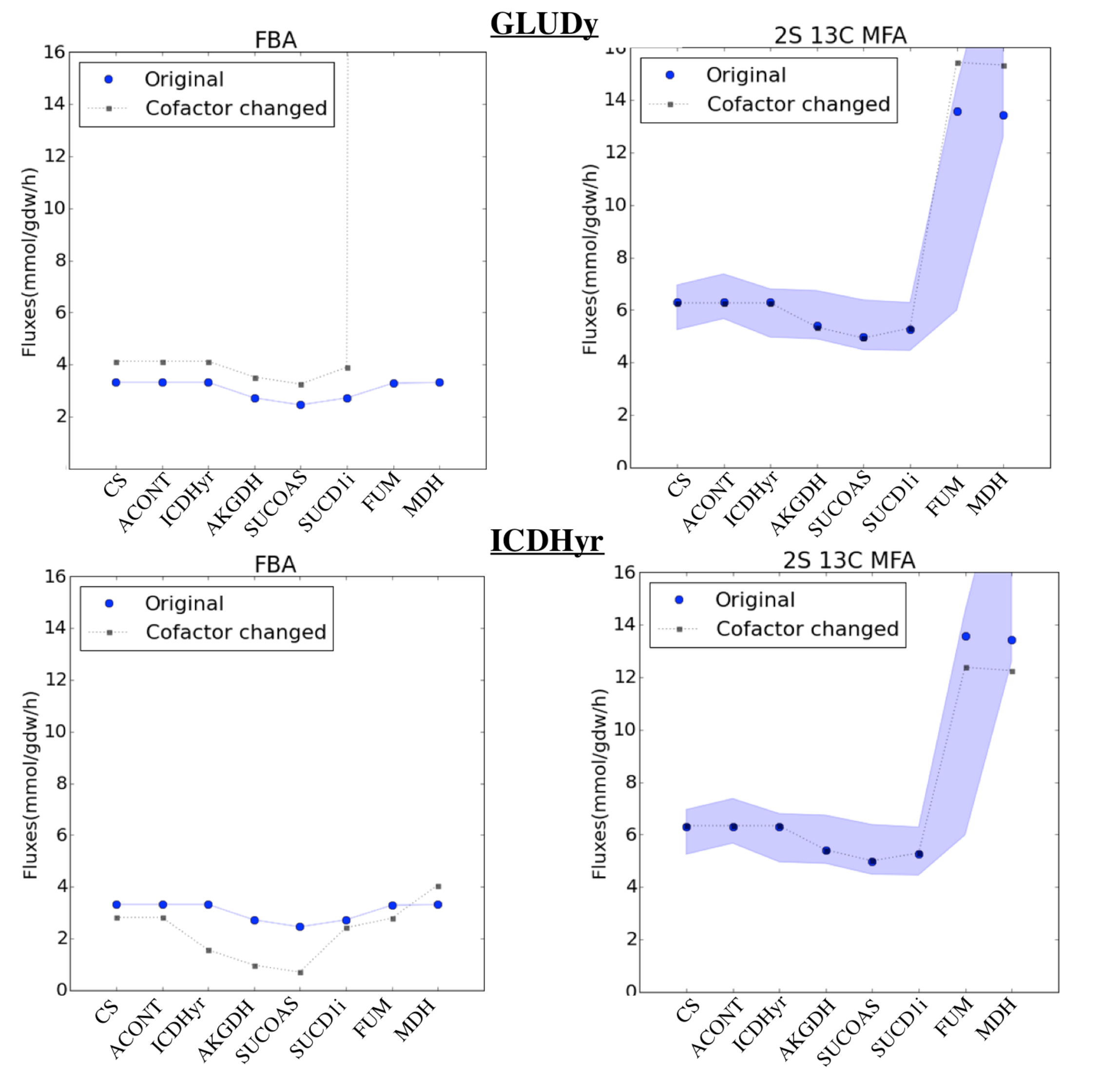

Supplement: S16 Fig — Since the flux value for SUCD1i is negative, the absolute value has been plotted. (TIF) [file pcbi.1004363.s020.tif]

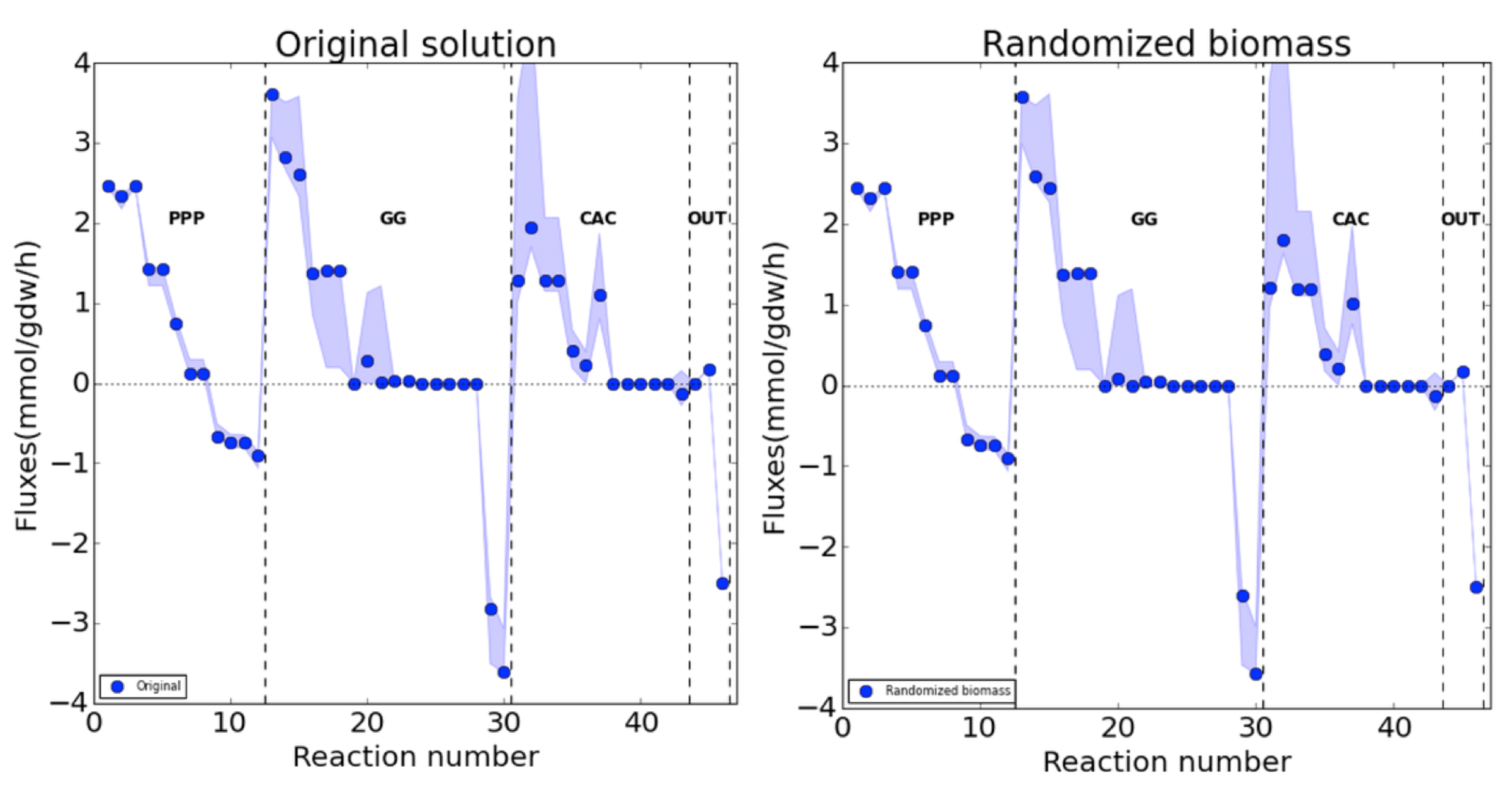

Supplement: S17 Fig — The 13C labeling data constrains fluxes strongly to a particular solution whereas changes in biomass requirements can be easily accommodated by the increased degrees of freedom found in genome-scale models. (TIF) [file pcbi.1004363.s021.tif]

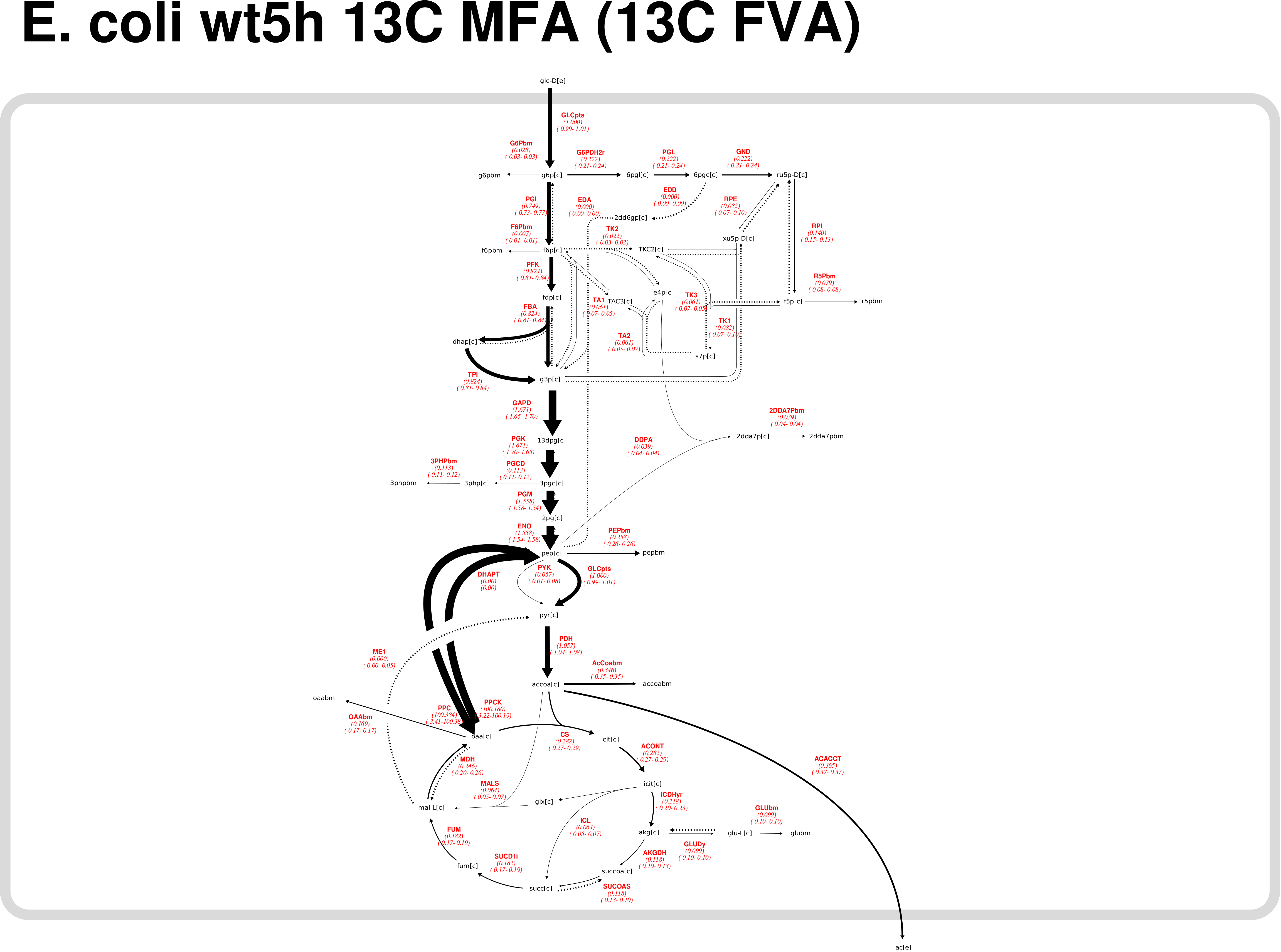

Supplement: S18 Fig — (TIF) [file pcbi.1004363.s022.tif]

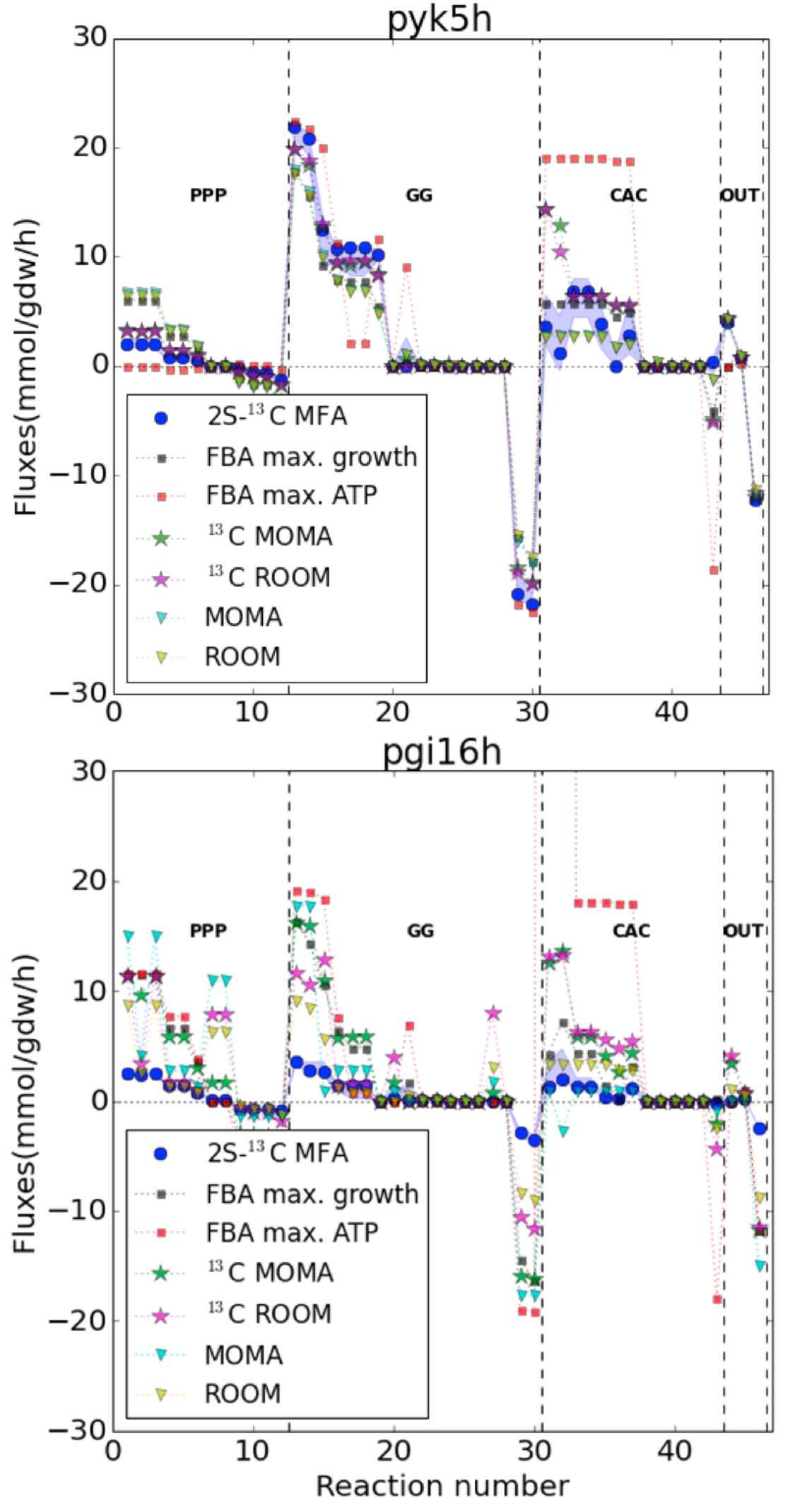

Supplement: S19 Fig — Full prediction means that no data from the target experiment was used to constrain fluxes: all predictions were derived from data from a different experiment. 2S-13C MFA profiles are found by solving Eqs 1–7. Maximum growth and ATP profiles are found by solving equations 1–3 in S1 Text. MOMA, 13C MOMA, ROOM and 13C ROOM flux profiles are obtained as explained in S3 Text. Fluxes are sorted according to the following order; (PPP): G6PDH2r, GND, PGL, RPE, TK1, TA2, EDA, EDD, TK2, TA1, TK3, RPI; (GG): GAPD, ENO, PDH, TPI, PGI, FBA, PFK, F6PA, GLCS1, GLGC, FBP, G1PP, GLCP, HEX1, PPS, PYK, PGM, PGK; (CAC): FUM, MDH, ACONT, CS, ICDHyr, SUCD1i, AKGDH, CITL, FRD2, FRD3, MDH2, MDH3, SUCOAS; (OUT): EX_ac(e), BiomassEcoli, EX_glc(e). All reaction names according to iJR906 [57]. (TIF) [file pcbi.1004363.s023.tif]

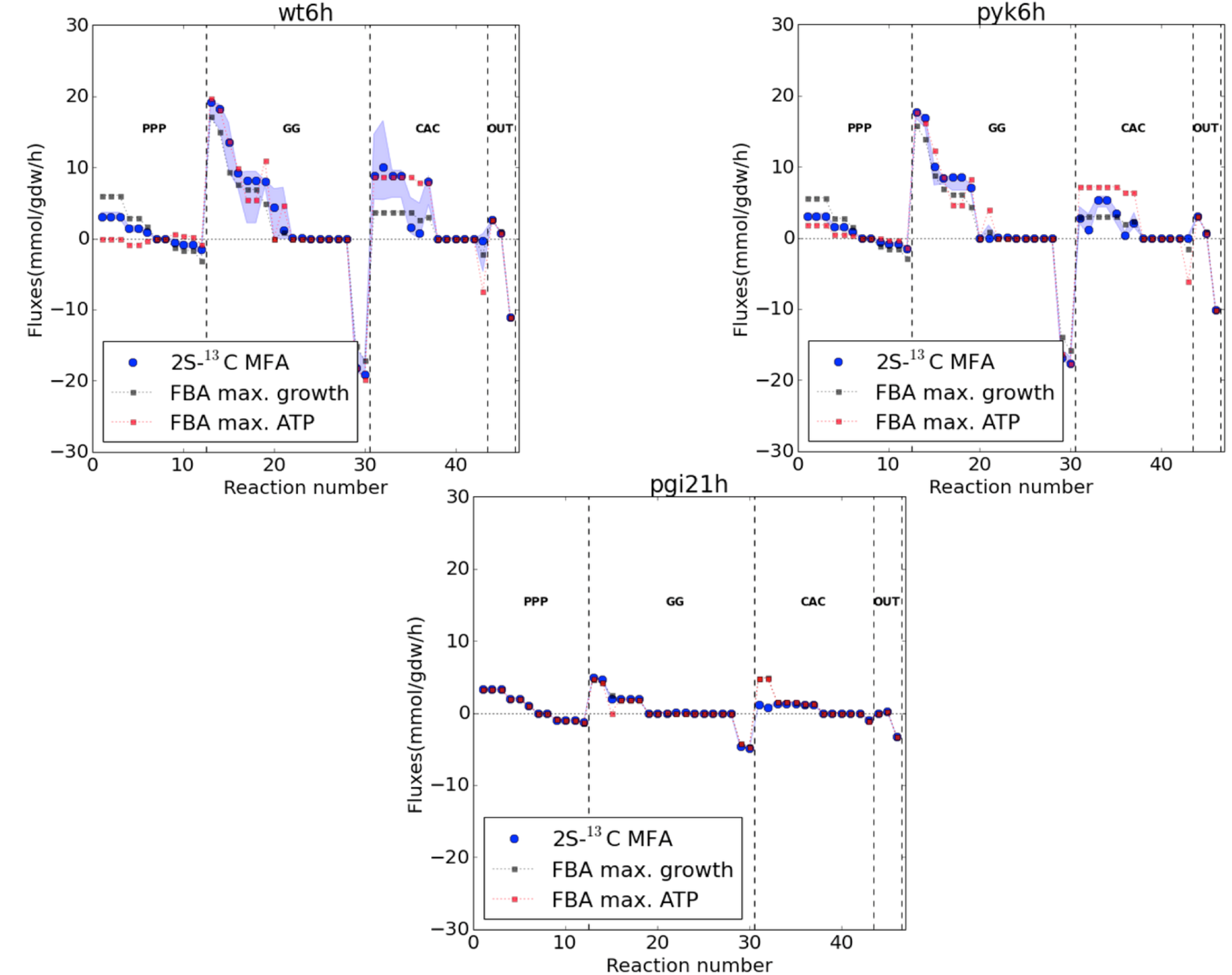

Supplement: S20 Fig — Partial prediction means data from the target experiment was used to constrain fluxes, in this case the values for the growth rate, glucose intake and acetate excretion rate. 2S-13C MFA profiles are found by solving Eqs 1–7 in the main paper. Maximum growth and ATP profiles are found by solving equations 1–3 in S1 Text. Transparencies indicate confidence intervals for the 2S-13C MFA. (TIF) [file pcbi.1004363.s024.tif]

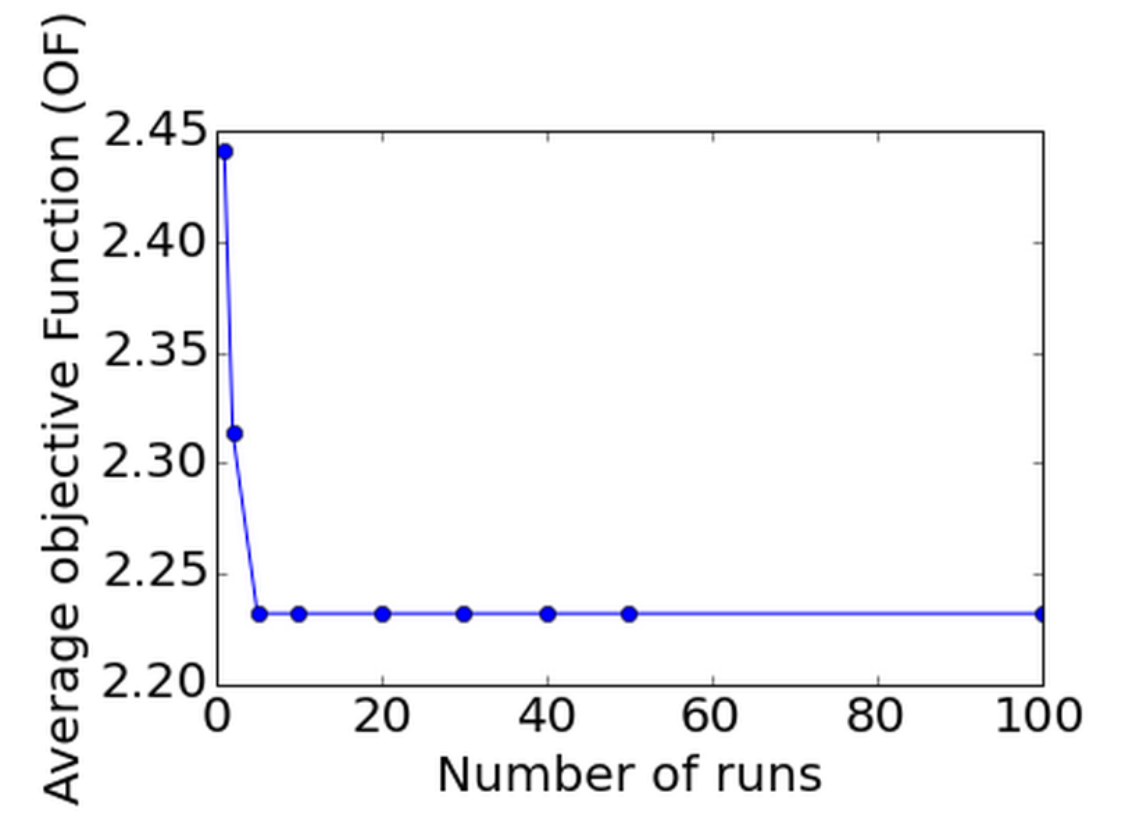

Supplement: S21 Fig — OF plateaus at N ≈ 15. We chose N = 30 for our simulations. (TIF) [file pcbi.1004363.s025.tif]

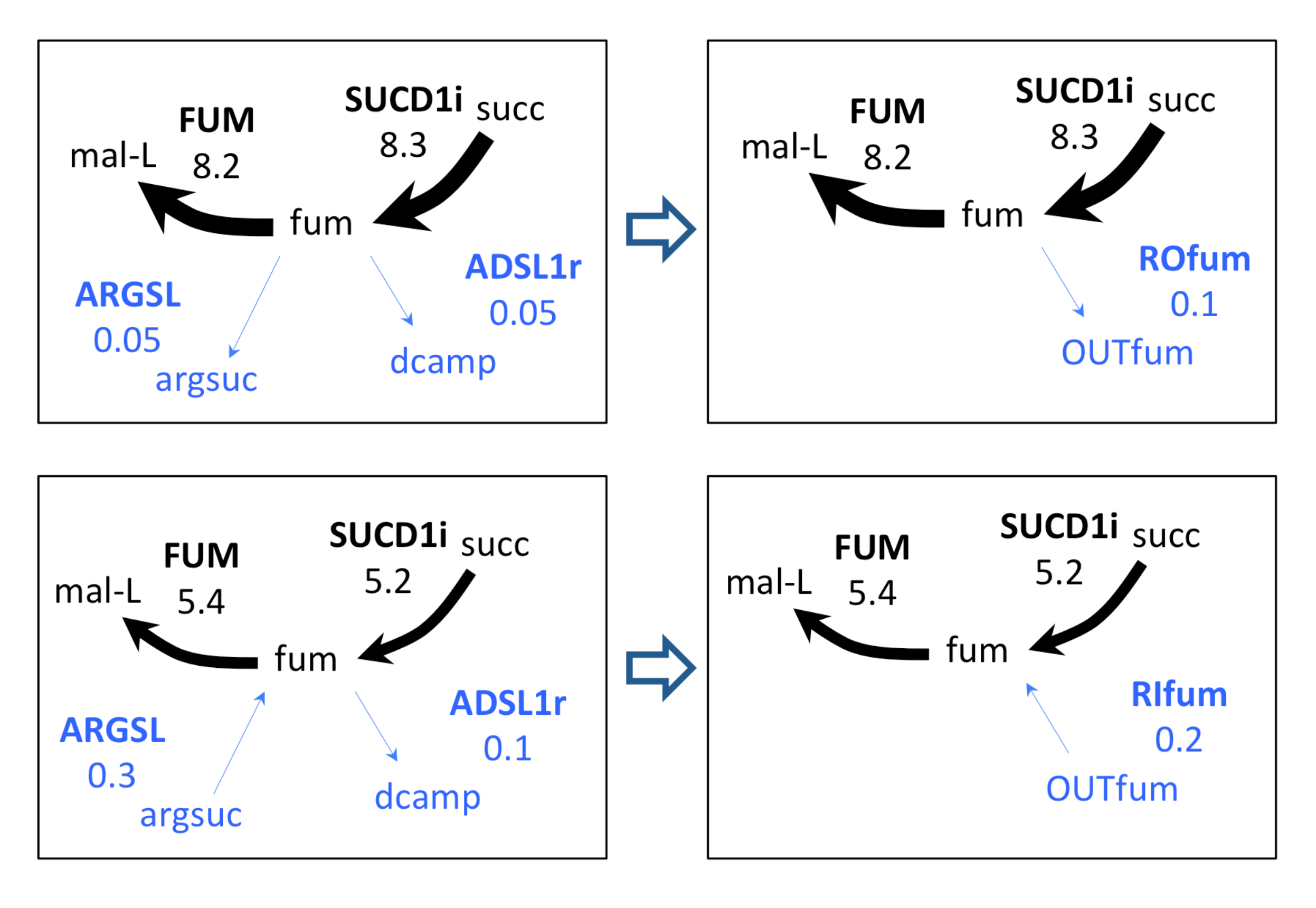

Supplement: S22 Fig — The purpose of ELVA is to determine if the reactions left out of the core metabolism significantly affect core metabolite labeling. In order to do so, only the core metabolism network is used and non-core metabolism is represented through inflow reactions and metabolites. Inflow reactions and metabolites are dummy reactions and metabolites that aggregate the non-core effects. In the figure, black denotes core metabolites and reactions and blue denotes noncore metabolites and reactions. Inflow reactions and metabolites are added to the core set to meet stoichiometric requirements (since core fluxes are fixed to the values obtained in the previous “Fit data” step). For example, the upper figures show how reactions ARGSL and ADSL1r are combined into ROfum leading into the dummy metabolite OUTfum, while keeping the same net flux out of fumarate. In the case of the lower figures ARGSL and ADSL1r have a net flux into the core set and are substituted by a inward flowing dummy reaction RIfum and a dummy metabolite OUTfum. The point of the ELVA is to elucidate the impact of the metabolites in the noncore set (see materials and methods). Outflowing reactions have no effect (upper panels) but inflowing reactions do (lower panels). The labeling of incoming dummy metabolites is left unconstrained since its value is not being tracked and our goal is to determine what is the maximum effect they may have on the measured metabolite labeling. (TIF) [file pcbi.1004363.s026.tif]
